# Lignin Selection Improves the Performance of Porous Carbon Nanofiber Electrodes in Freestanding Supercapacitors

**DOI:** 10.1021/acssuschemeng.6c00388

**Published:** 2026-06-01

**Authors:** Robert D. Hunter, Sarah Seidner, Caitlin Brooker-Davis, Milo S. P. Shaffer, Agnieszka Brandt-Talbot, Maria-Magdalena Titirici

**Affiliations:** † Department of Chemical Engineering, 4615 Imperial College London, London SW7 2AZ, U.K.; ‡ Department of Chemistry, 4615 Imperial College London, London W12 0BZ, U.K.; § School of Chemistry, University of Birmingham, Edgbaston B15 2TT, U.K.; ∥ Department of Materials, 4615 Imperial College London, South Kensington Campus, London SW7 2AZ, U.K.; ⊥ Advanced Institute for Materials Research (WPI-AIMR), Tohoku University, 2-1-1 Katahira, Aobaku, Sendai, Miyagi 980-8577, Japan

**Keywords:** electrospinning, ionosolv, Kraft, organosolv, water-in-salt electrolyte, multivariate correlations, microporosity, hydroxyl groups, viscosity, molar weight, carbon yield

## Abstract

Lignin is an attractive precursor for renewable carbon materials due to its low cost and comparatively high carbon yield. The resulting carbons may be used in energy storage devices (supercapacitors and batteries) and structural composites. Since lignin has substantial variability in its chemical structure, influenced by its botanical origin and the method of extraction, this work explores the effect of lignin type on the structure and performance of porous carbon materials, using electrospun carbon nanofibers applied as freestanding supercapacitor electrodes as an example. Precursor nanofiber mats were electrospun from aqueous NaOH solutions of six technical lignins, which originated from commercially relevant biomass types, i.e., hardwood (eucalyptus and beech), softwood (pine/spruce mix), and grass (*Miscanthus*) and three extraction methods (ethanol organosolv, Kraft, and ionosolv), with poly­(ethylene oxide) (PEO) as the spinning aid. After stabilization at 250 °C and carbonization/activation at 1000 °C, the supercapacitor performance was evaluated in symmetric two-electrode cells using an aqueous electrolyte (6 M KOH). Correlation of a large number of microstructural characteristics and lignin chemical properties showed that a high micropore (1–2 nm) volume increased gravimetric capacitance of the lignin-derived carbon nanofiber (LCNF) mats (up to 192 F/g at 0.25 A/g), while a high packing density maximized volumetric capacitance (up to 19.9 F/cm^3^ at 0.25 A/g). The packing density of the LCNF electrodes was strongly correlated to the viscosity of the lignin–PEO solution, showing potential for engineering material performance through spinning solution composition. Technical lignins with higher contents of phenylpropanoid linkages and hydroxyl groups, especially phenolic hydroxyl groups, exhibited increased microporosity and hence gravimetric capacity but also decreasing packing density and hence volumetric capacitance, indicating a trade-off situation. Finally, by changing the aqueous electrolyte to a water-in-salt electrolyte (12 mol/kg NaNO_3_), the energy density of the best-performing supercapacitor cell was increased from 8.9 to 15.5 Wh/kg, due to the higher stable operating voltage (1.8 vs 1.2 V), delivering competitive performance with typical activated carbon powder-based supercapacitors (5–15 Wh/kg) and highlighting the importance of the whole device design for boosting performance.

## Graphical abstract

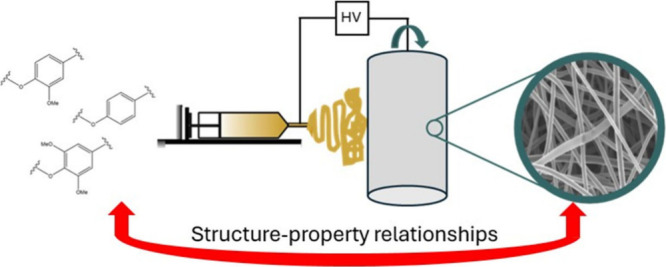

## Introduction

Electrochemical capacitors are high power devices that are used for stationary or mobile energy storage applications due to fast charging and long cycle life.^1^ Most supercapacitor devices rely on electrochemical double-layer capacitance and utilize nanoporous carbon materials as the active component in the electrode composition.^1,2^ Supercapacitors are typically employed because of their high power densities (∼10 kW/kg) rather than their energy density, which is typically in the 5–20 Wh/kg range, while battery devices have energy densities of 200–300 Wh/kg. To widen the commercial applicability of supercapacitors while keeping manufacturing cost low, it is desirable to increase the gravimetric and volumetric energy density of the electrode and the whole supercapacitor device using cost-effective raw materials and manufacturing approaches.

It is known that pore structure is important for the performance of carbon electrode materials, and templating, physical and chemical activation are used to enhance the pore structure.^3^ A maximum in the capacitance has been reported as the pore sizes in the active carbon materials decrease toward the size of desolvated electrolyte ions,^4–7^ while pore connectivity and the pore size distribution have also been found to be important for maximizing performance.^8–12^ In hierarchical porous carbon structures, the presence of mesopores has been shown to facilitate ion transport to micropores, leading to increased capacitances and rate capabilities.^13^ Other studies have, however, shown a lack of correlation between the pore size and capacitance in a range of microporous activated carbons.^14–16^ More recently, the atomic arrangement in the carbon structure was shown to affect a carbon material’s capacitance.^17,18^ For example, the capacitance of commercial activated carbons has been positively correlated with the presence of smaller graphene-like domains.^17,18^ Therefore, consideration of multiple characteristics of a carbon material is important to understand its behavior as an electrode in an electrochemical double-layer capacitor (EDLC).

The energy density (*E*) of an EDLC depends on both specific capacitance (*C*) and the working voltage (*V*) according to the relationship *E* = 1/2*CV* ^2^. Therefore, there is interest in developing novel electrolytes that offer a wide stable operating voltage while maintaining high ionic conductivity and low viscosity as well as cost-effectiveness and safety. Aqueous electrolytes such as 6 M KOH are commonly reported in the literature for their high ionic conductivity and low cost. However, aqueous electrolytes generally have a relatively narrow operating voltage window, limited by the water splitting voltage (1.23 V).^19,20^ Therefore, organic electrolytes with wider electrochemical windows (>3.0 V) such as 1 M tetraethylammonium tetrafluoroborate (TEABF_4_) in acetonitrile are used in commercial devices, despite their greater toxicity, flammability and environmental footprint. Recently, water-in-salt (WIS) electrolytes consisting of highly concentrated aqueous salt solutions have been explored as safer alternatives, offering relatively high voltage windows (up to ∼3 V).^21,22^

To fabricate an electrode for a standard supercapacitor device, a slurry consisting of the active material, a conductive additive and a binder are prepared in a solvent, which is often hazardous, for example, *N*-methyl-2-pyrrolidone, and coated onto a metal foil or grid that acts as the current collector.^23,24^ While the precursor for the electrode material can be renewable, the additional components add parasitic mass and can introduce various resistive interfaces, lowering the total gravimetric and volumetric energy density of the device while increasing the cost of the device, economically and environmentally. Consequently, there is significant interest, from both a cost and sustainability perspective, in replacing traditional powder-based supercapacitor electrode assemblies with free-standing electrodes that can act as the active material and the current collector without using polyfluorinated polymer binders or conductive additives.^25^

Electrospinning is utilized to prepare spun polymer nanofibers mats from polymer solutions, which can be carbonized “under activating conditions” to yield carbon nanofiber mats that are conductive, mechanically resilient and flexible,^25^ and hence capable of acting as freestanding electrodes. Carbon fibers can be produced from a range of precursor materials, including mixtures of two or more polymers, with examples listed in Table S1. The majority of commercial carbon fibers are derived from polyacrylonitrile (PAN), due to its good spinnability, high carbon yield (∼50%) and tendency to produce ordered (turbostratic) carbons structures, which have higher conductivity than hard carbons.^26,27^ However, PAN is a petroleum-derived polymer that generates toxic byproducts such as HCN during carbonization and requires expensive solvents such as *N*,*N*-dimethylformamide (DMF) and dimethyl sulfoxide (DMSO) during spinning.^28^ Hence, there has been interest in using renewable, biobased precursors such as lignin to produce sustainable carbon fiber-based materials.

Lignin is a promising renewable precursor for carbon fiber due to its low cost and relatively high carbon yield (∼40%). For example, recent work has demonstrated that lignin-derived carbon fibers can be produced at a lower cost than PAN carbon fiber and with a lower predicted global warming potential (GWP) of 15–25 kg CO_2_-eq, compared to 27–41 kg CO_2_-eq for PAN, with a even lower GWP if carbon credits are allocated for substitution of PAN with bioderived precursors.^29^ As one of the three major components of lignocellulosic biomass, lignin is highly abundant in nature and a low-value byproduct of cellulose production through wood pulping or fractionation.^30^ 50–70 million tonnes of lignin are extracted annually at pulp and paper facilities worldwide, with availability of a wider variety of lignins expected due to increased demand for second generation biorefining products.^31^ Lignin is a diverse group of macromolecules and generated during lignification of the plant cell wall by oxidative radical polymerization of monolignols.^32^ A variety of aliphatic linkages are formed, most commonly in the form of ether (C–O–C) and carbon–carbon bonds, with aliphatic hydroxyl groups present on the linker units. Lignin primarily consists of two aromatic subunits, syringyl (S) and guaiacyl (G), but other subunits can also be also present, such as *p*-hydroxyphenyl (H), *p*-coumaric acid (PCA), or tricin.^32,33^ The relative proportions of the subunits vary significantly between different botanical origins. Hardwood lignins contain both S and G subunits (typically more S),^34^ softwood lignins contain G subunits and grass lignins contain S and G (roughly in even proportions), H, PCA, and tricin.

In addition, the extraction method used to isolate lignin from raw lignocellulosic biomass alters the structure of lignins. The reactive extractions needed to disassemble the biomass affect chemical functionality (breaking of linkages and forming new ones), functional group content, molecular weight distribution, and impurity content. A key method for extracting lignin is the Kraft pulping process, in which sodium hydroxide and sodium sulfide are used to extract lignin from wood biomass.^31^ The cellulosic component forms a pulp, which has uses in printing, packaging, textile and hygiene applications, while the lignin is dissolved in the black liquor; it is typically not isolated but incinerated during chemical recovery. However, if desired, the lignin can be precipitated using acid.^35^ The structure of the resulting Kraft lignin is considerably different to the native lignin in the lignocellulosic feedstock. Kraft pulping produces hydrolyzed and condensed lignin that contains considerable impurities, such as carbohydrates, organic sulfur and ash (majority sodium salts). The isolated Kraft lignin market is currently small, hence there is no commodity price available; in addition, the price of isolated Kraft lignin varies with purity.^36^ Kraft lignin prices reported in the literature range from 220 to 650 €/t.^37^ During organosolv pulping, lignin is extracted using an organic solvent/water mixture, most commonly ethanol, often with the addition of an acid to catalyze the breakdown of lignin-carbohydrate linkages.^38^ The lignin is precipitated by removing the more volatile organic solvent or by increasing the water content. Organosolv lignins are typically free of organic sulfur, have low carbohydrate and inorganic impurities and a relatively narrow range of molar weights.^39,40^ They may be modified by lignin reacting with the organic solvent component; for example, ethoxylation has been observed for ethanol organosolv lignins. The recovery of the relatively costly organic solvent by fractional distillation remains a challenge for the economic viability of organosolv-based biorefineries.^41^ As a result, organosolv lignins are generally more expensive than Kraft lignins, with reported estimated costs of 1100–1250 €/t.^42^ More recently, the use of ionic liquids in the ionosolv fractionation has been explored as a potentially lower cost alternative to Kraft and organosolv pulping.^43^ Protic ionic liquid/water mixtures are used to extract lignin and hemicellulose from lignocellulosic biomass, and lignin is isolated by increasing the water content in the solvent.^44–46^ The lignin is hydrolyzed and condensed, with inorganic sulfur (residual anion) and organic nitrogen (residual cation) present and a broad molar weight distribution. The advantage of ionic liquids over the organic solvents is the low volatility, which simplifies separations and increases recovery of the more expensive, nonaqueous solvent component.^47,48^ Previous work has shown that protic ionic liquids can be produced at low cost and recycled with high recovery (>99.5%) and without affecting the fractionation performance,^49,50^ demonstrating potential for a robust, low-cost lignocellulose fractionation process. If the use of water in lignin-isolation can be optimised^51^ process models suggest that an industrial ionosolv process has economic benefits in comparison with other lignin isolation methods.^50^

There has been limited work on comparing the performance of different lignin feedstocks and extraction methods in carbon nanofiber-based supercapacitor electrodes. Comparison of electrospun carbon nanofibers derived from solvent fractionated hardwood and softwood Kraft lignins showed that the hardwood lignin resulted in a carbon nanofiber mat with a more developed pore structure, containing a mixture of nanopores and small mesopores, which was linked to better gravimetric performance (164 F/g for the hardwood and 150 F/g for the softwood at a current density of 0.1 A/g) in the freestanding supercapacitor electrode application,^52^ with the enhancements assigned to less rigid polymers in hardwood lignin and different reactivity during pyrolysis.^52^ The study highlighted the importance of considering the lignin chemical structure when optimizing performance of the resulting electrodes and devices.

The majority of studies reporting electrospun carbon nanofiber materials as supercapacitor electrodes focus on maximizing their gravimetric performance (Table S1).^53,54^ However, electrospun nanofibers generally have low packing density due to the abundance of free void space between the nanofibers themselves.^53^ Mechanical compression of electrospun ethanol organosolv lignin-derived nanofibers before the carbonization step has been explored as a means of densification.^53^ Applying an optimum pressure of 40 bar was found to reduce the void volume between the nanofibers, enhancing the volumetric capacitance and energy density of the electrodes from 20 to 130 F/cm^3^ and from 0.8 to 6 Wh/L at 0.1 A/g, respectively, without degrading their gravimetric capacitance (200 F/g at 0.1 A/g).^53^ The density and porosity of carbon electrodes have been reported to have a competitive relationship.^54^

This study explores a set of six lignins, expanding from the two Kraft lignins to include a wider variety of feedstocks and lignin extraction methods, including a grass lignin, a hardwood lignin obtained from ethanol organosolv pulping and, for the first time, three ionosolv lignins. The electrospinning used the industrially produced, nontoxic, potentially renewable polymer PEO as fiber spinning aid. Multivariate correlations were used to systematically identify properties of the lignin-derived carbon nanofibers (LCNFs) that give rise to the best electrochemical performance in terms of both gravimetric and volumetric capacitance and whether they are linked to the chemical structure of the employed technical lignins. Finally, the use of a WIS electrolyte in combination with the LCNF electrodes is demonstrated as a means of improving the gravimetric energy density of the best performing lignin derived freestanding supercapacitor device.

## Experimental Section

### Materials

Poly­(ethylene oxide) (200000 g/mol), poly­(ethylene oxide) (400000 g/mol), sodium hydroxide (ACS reagent, ≥97.0%, pellets), hydrochloric acid (37%, ACS reagent), potassium hydroxide (≥99.95%), and sodium nitrate (ReagentPlus, ≥99.0%) were all purchased from Sigma-Aldrich.

### Isolation of Organosolv Beech Lignin

Organosolv lignin extracted from beech wood was provided by the Fraunhofer Center for Chemical-Biotechnological Processes (CBP) in Jena. The beech wood was chopped into pieces and digested at 170 °C for 80 min in a 50 wt % ethanol/water mixture containing 1% H_2_SO_4_. The resulting black liquor was then diluted with H_2_O [ratio of 2:1 (v/v) water/black liquor] to precipitate the lignin with a yield of 64 wt % relative to the lignin content in the initial dry wood mass.

### Isolation of Kraft Eucalyptus Lignin

The eucalyptus black liquor was obtained from the ENCE pulp mill (Huelva, Spain) and isolated using the LignoBoost process.^55^ The lignin was fractionated as described in a previous publication.^56^

### Isolation of Kraft Pine/Spruce Lignin

A softwood (pine/spruce mixture) Kraft lignin was obtained using LignoBoost precipitation (Bäckhammar, Sweden). The lignin was dried at 80 °C under vacuum for approximately 12 h. A sequential solvent extraction was used to produce a lignin fraction suitable for electrospinning. The solvent extraction method was a simplified version of the method described by Baker and Hosseinaei.^57^ The dry lignin (1000 g) was first extracted with methanol by rapidly stirring in 10 L methanol at room temperature for 2 h. The sample was filtered and the filter cake extracted using a 70:30 (v/v) mixture of methanol and dichloromethane (1:10 solid-to-liquid ratio, room temperature, 4 h). The extract solution was filtered, and lignin isolated from the filtrate using a rotary evaporator. The isolated lignin was dried in a vacuum oven at 80 °C for 12 h. The yield for this lignin fraction was 42.5% relative to the crude LignoBoost lignin.

### Isolation of Ionosolv Eucalyptus Lignin

Ionosolv lignin was extracted using a procedure reported previously.^58,59^ A total of 30 g (dry-basis) of air-dried, ground, and sieved (180–850 μm) eucalyptus wood was added to a 200 mL pressure tube (Ace Glass, front sealing), followed by the addition of 150 g of the ionic liquid *N*,*N*-dimethylbutylammonium hydrogen sulfate, [DMBA]­[HSO_4_] (20 wt % water content). The biomass and ionic liquid were mixed with a vortex shaker, then placed in a preheated oven for 1 h at 170 °C. The resulting cellulose pulp was washed with 600 mL of absolute ethanol, shaken and left to rest for 1 h at room temperature. The mixture was separated into a cellulose-rich solid and a liquid using vacuum filtration. The washing steps were repeated three times, and each time the liquid portion was collected. The cellulose pulp was air-dried, while the ethanol was evaporated from the combined liquid with a rotary evaporator to obtain the lignin-rich liquor. The lignin was precipitated by the addition of 400 mL of deionized water, and the mixture was transferred into a 500 mL centrifuge tube, mixed using a vortex shaker and rested for 1 h before centrifugation at 3000 rpm for 50 min (Megastar 3.0, VWR, UK). The supernatant was carefully decanted, and the precipitate was washed with deionized water three times and freeze-dried for 48 h to isolate a lignin product. The yield for this lignin was 75% relative to the lignin content of the eucalyptus wood.

### Isolation of Ionosolv Spruce and Ionosolv Miscanthus Lignins

An ionic liquid water mixture was prepared by adding 20 wt % water to the ionic liquid *N*,*N*-dimethylbutylammonium methanesulfonate, [DMBA]­[MeSO_3_]. The water content was confirmed by Karl Fischer titration. A total of 30 g (dry-basis) of air-dried, ground, and sieved (180–850 μm) spruce or miscanthus was added to a pressure tube, followed by the addition of 150 g of the ionic liquid water mixture. The pressure tubes were sealed, and the content mixed with a vortex shaker. The samples were then placed into a preheated convection oven (OMH60 Heratherm Advanced Protocol Oven) at 170 °C for 1 h for spruce and 150 °C for 1 h for miscanthus. The resulting cellulose pulp was washed with 600 mL absolute ethanol, shaken and left to rest for 1 h at room temperature. The solution was mixed with a vortex shaker and centrifuged for 30 min at 3600 rpm. The supernatant was decanted, and the step repeated four times. The remaining pulp was transferred into a thimble and washed by Soxhlet extraction with refluxing ethanol (120 mL) for 16 h. The thimbles were left in a fume hood to dry at room temperature. The ethanol used for the Soxhlet extraction was combined with the previous washes and evaporated under reduced pressure at 40 °C. Lignin was precipitated by adding 4 g water per 1 g IL as an antisolvent to the remaining ionic liquid/lignin liquor. The solution was mixed using a vortex mixer and the solution left to sit for 1 h, followed by centrifuging and decanting of the supernatant. The washing step was repeated three times until the solution was clear. The Falcon tubes containing the lignin were pierced and the tube placed inside a freeze-dryer for 48 h. The yields for the spruce and miscanthus lignins were 100% and 74%, respectively, relative to the lignin content of the initial biomass.

### Preparation of Electrospun LCNF Mats

A total of 0.15 g PEO (unless otherwise stated) was dissolved in 7.8 g of aqueous NaOH (0.5 M) and stirred for 24 h. Subsequently, 1.2 g lignin was added, and the solution stirred for 24 h. The solution was loaded into a plastic syringe (10 mL) and electrospun within a Nanobox chamber (Plaslab), where the relative humidity was maintained at 25%. The syringe was discharged at 1 mL/h with a single syringe infusion pump (KDS-100-CE, KD Scientific). A voltage of 20 kV was applied using a transformer (Glassman High Voltage Inc., USA) between the syringe needle (21 Gauge, Hamilton) and a rotating drum collector. The rotating collector was placed 20 cm away from the tip of the syringe needle, and the rotation speed was maintained at 500 rpm. After spinning 6 mL of the solution, the lignin fiber mats were removed from the collector and cut into strips before being sandwiched between two pieces of carbon felt and subjected to heat treatment in an MTI 1200x tubular furnace. The fiber mats were thermally stabilized by heating under a flow of compressed air (300 mL/min) at a heating rate of 1 °C/min to 250 °C for 2 h. The stabilized fiber mats were carbonized at 1000 °C for 2 h under a nitrogen atmosphere (500 mL/min) using a heating rate of 1 °C/min. The carbonized fiber mats were submerged in 0.5 M HCl solution for 30 min, followed by rinsing with deionized water and ethanol, and air-dried for 24 h before storage in a vacuum oven at 70 °C.

### ^1^H–^13^C Heteronuclear Single-Quantum Coherence (HSQC) NMR Spectroscopy

The lignins were dissolved in dimethyl sulfoxide-*d*_6_ 99.9 atom % at a concentration of 20 mg/mL, and HSQC NMR spectra were recorded on a Bruker 800 MHz spectrometer. The resonance signal of residual (CD_3_)_2_SO at 2.5 ppm (^1^H) and 40 ppm (^13^C) served as a reference for the chemical shift. The aromatic subunit composition was estimated by integrating the G_2_ signal and S_2,6_ signal (including the signals for the condensed variants) and calculating the ratio of S to G. The G_2_ signal was selected as it has been previously shown to be the most stable correlation.^50^ The amount of the side-chain linkages and carbohydrates was estimated by signal integration of the C_α_-H_α_ correlations of the three main aliphatic linkages (β-O-4, β-β, and β-O-5) and the anomeric carbon of xylose (X_1_).^52^

### Hydroxyl Group Analysis

^31^P NMR spectroscopy was used to quantify the hydroxyl groups after derivatization of lignin with 50 μL 2-chloro-4,4,5,5-tetramethyl-1,3,2-dioxaphospholane (TMDP) using previously reported procedures.^60,61^ A total of 10 mg lignin was dissolved in 100 μL anhydrous pyridine and deuterated chloroform in a 1.6:1 (v/v) ratio and mixed with 50 μL cyclohexanol and 50 μL of a 6 mg/mL chromium acetylacetonate solution as internal standard and relaxation agent, respectively. ^31^P NMR spectra were acquired using a 500 MHz Bruker Advance spectrometer using an inverse-gated decoupling pulse sequence with a 25 s delay to avoid nuclear Overhauser effects to obtain quantitative spectra. A 90° pulse angle and 4.0 Hz line broadening was used. A minimum of 200 transients were acquired for each sample at room temperature. Peaks were assigned and integrated according to previous literature and compared to the peak integral of the internal standard.

### Molar Weight Measurements

Samples were dissolved in DMSO/LiBr (0.5%) to achieve a concentration of 10 mg/mL. Prior to gel permeation chromatography (GPC) analysis, the solutions were filtered through 0.45 μm PTFE syringe filters. All samples were dissolved and measured in duplicate. GPC analysis was performed by using an Ultimate 3000 autosampler, column oven, UV detector (all Thermo Fisher Scientific Inc., Waltham, MA, USA) equipped with a Dionex HPLC Pump Series P580 (Dionex Softron GmbH, Germering, Germany), Dawn HELEOS I MALS detectors with lasers operating at 785 nm, and an Optilab T-rEX differential refractive index detector, λ = 633 nm (all Wyatt Technology, Santa Barbara, CA, USA). The MALS detector was equipped with 18 photodiodes at different measuring angles, with narrow band-pass filters (±10 nm for the respective wavelength used, installed on every second photodiode). The separation was performed with an Agilent PolarGel M guard column (7.5 × 50 mm) and three PolarGel M columns 7.5 × 300 mm (5 μm particle size). The columns were kept at 35 °C. The GPC system was operated using DMSO as the eluent and the following conditions: 0.5 mL/min flow rate; 10 μL injection volume; 65 min acquisition time. Data evaluation used ASTRA software, version 6.1.^62^

### Thermogravimetric Analysis

Thermogravimetric analysis was performed on a PerkinElmer TGA instrument. The samples were heated in platinum pans at 10 °C/min under a flow of nitrogen gas (50 mL/min) from 25 to 100 °C for 30 min to drive off moisture, before heating to 900 °C at a rate of 10 °C/min. The char yields at 900 °C are the averages of duplicate measurements. Ash content determination was performed on a PerkinElmer TGA instrument using a method validated by Aldaeus et al.,^63^ which is a miniaturized version of the method described in ISO 1762.^64^ A total of 5 mg lignin powder was heated in platinum pans at a rate of 15 °C/min under a flow of compressed air (50 mL/min) from 25 to 105 °C for 30 min to drive off moisture, before heating to 525 °C at a rate of 15 °C/min and holding for 2 h.

### Viscosity Measurements

The viscosity of the lignin/PEO/NaOH solutions was measured using an AR 2000ex rheometer with a cone-and-plate feature (2° cone angle, 20 mm plate diameter, and 53 μm gap) and stainless-steel Peltier plate at shear rates from 1 to 1000/s at room temperature. Steady-state flow measurements were carried out to determine the effect of an increasing shear rate on the dope solutions. A preshear conditioning phase (shear rate 1/s, 1 min) was applied to remove air bubbles in the sample. Each measurement was collected at a point time of 30 s.

### Scanning Electron Microscopy (SEM)

LCNF samples were cut into small sections and fixed to an aluminum stub using carbon adhesive tape. SEM images of the LCNF samples were recorded on a JEOL JSM-6010LA microscope using secondary electron imaging (SEI) with an accelerating voltage of 20 kV. Fiber diameters were determined using ImageJ software, and average diameter values are reported with standard deviation.

### X-ray Diffraction (XRD) Measurements

LCNF samples were analyzed using powder XRD (STOE Stadi-P, Debye–Scherrer mode, Mo source, λ = 0.7093 Å). Materials were loaded into Kapton capillary tubes and sealed with vacuum grease prior to measurement. Data were collected between 2 and 80° 2θ, for 3600 s per 6° step of the PSD.

### Raman Spectroscopy

Raman spectra were collected on a Renishaw inVia micro-Raman (500–3000 cm^–1^), using a 50 mW 532 nm laser at 10% laser power. Statistical Raman data were obtained from measurements by performing a map of at least 25 points per sample. All Raman spectra were normalized by maximum intensity, baseline-subtracted, and had cosmic rays removed.

### Four-Point Probe Conductivity

The electrical conductivity was measured using a Jandel cylindrical four-point probe head with 1 mm needle spacing and 300 μm diameter. The apparatus was connected with Keysight 34410A digital multimeter and the measured resistance was interpreted using a thin infinite plane assumption.

### X-ray Photoelectron Spectroscopy (XPS)

XPS analysis was performed using a Thermo Fisher K-alpha XPS system equipped with an Al Kα monochromated X-ray source, and the acquired spectra were analyzed using the Avantage software.

### Gas Sorption Measurements

Physisorption measurements were performed using a Micromeritics TriStar II PLUS instrument at 77 and 273 K for N_2_ and CO_2_, respectively. Samples were degassed at 200 °C for 16 h under vacuum with Micromeritics’ Smart VacPrep and the Smart VacPrep software. The specific surface area was calculated from N_2_ isotherms by applying the Rouquerol correction to select an appropriate pressure range.^65^ Pore size distributions were calculated from both N_2_ and CO_2_ isotherms using a 2D nonlocal density functional theory (2D-NLDFT) kernel for carbon with a slit pore geometry.^66^

### Helium Pycnometry

The skeletal (true) density of the LCNF mats was measured using a Micromeritics AccuPyc gas pycnometry system, using helium as the displacement medium.

### Electrochemical Testing

The LCNF mats were evaluated as electrodes in symmetric supercapacitor cells. All measurements were performed in a two-electrode setup consisting of two CNF electrodes in circular shapes. The cells were assembled in a Swagelok cell and connected to a VSP-potentiostat (Biologic). The carbon electrodes were punched with a diameter of 1.2 cm and directly placed on stainless steel current collectors. 1.2 cm circles were punched from glass fiber filter paper (Whatman, grade GF/D) and used as a separator. The cells were assembled by soaking the separator in electrolyte (6 M KOH or 12 mol/kg NaNO_3_) and pressing between the two electrodes and current collectors (further details in Table S2). Prior to measurements, 500 cycles were run at 5 A/g to fully wet the electrodes with electrolyte. Cyclic voltammetry (CV) was recorded at various scan rates, galvanostatic charge–discharge (GCD) at different current densities, and electrochemical impedance spectroscopy (EIS) between 200 kHz and 10 mHz with a signal amplitude of 5 mV. The total capacitance, *C*_total_, of the symmetric cells was calculated using eq 1, in which *I* is the constant charging/discharging current and Δ*V*/Δ*t* is the gradient of the discharge slope (excluding the ohmic voltage drop).^67^

1 $$C_{\mathrm{total}}=I/\left(\Delta V/\Delta t\right)\;\left(F\right)$$

Pseudo-single electrode gravimetric capacitance values, *C*_grav_, were calculated using eq 2, where *m*_ave_ is the average mass of one electrode in the cell [*m*_ave_ = 1/2­(*m*_electrode 1_ + *m*_electrode 2_)].^67^

2 $$C_{\mathrm{grav}}=2C_{\mathrm{total}}/m_{\mathrm{ave}}\;\left(F/g\right)$$

The volumetric (*C*_vol_) and areal capacitance (*C*_areal_) of pseudo-single electrodes were calculated using eqs 3 and 4, respectively:

3 $$C_{\mathrm{vol}}=C_{\mathrm{grav}}\left(m_{\mathrm{ave}}/v_{\mathrm{ave}}\right)\;\left(F/{\mathrm{cm}}^{3}\right)$$

4 $$C_{\mathrm{areal}}=C_{\mathrm{grav}}(m_{\mathrm{ave}}/a_{\mathrm{ave}})\;\left(F/{\mathrm{cm}}^{2}\right)$$

where *v*_ave_ and *a*_ave_ are the average volume and area of one electrode in the cell, respectively. The energy density of the cell (*E*_device_) was calculated from the specific capacitance of the full cell, *C*_sp_, using eqs 5 and 6, where *m*_cell_ is the total mass of the two electrodes in the cell (working and counter electrode). The power density (*P*_device_) was then calculated using eq 7, where Δ*t*_discharge_ is the discharge time:

5 $$C_{\mathrm{sp}}=C_{\mathrm{total}}/m_{\mathrm{cell}}\;\left(F/g\right)$$

6 $$E_{\mathrm{device}}=1/2C_{\mathrm{sp}}V^{2}\;\left(\mathrm{Wh}/\mathrm{kg}\right)$$

7 $$P_{\mathrm{device}}=E_{\mathrm{device}}/\Delta t_{\mathrm{discharge}}\;\left(\mathrm{kW}/\mathrm{kg}\right)$$

## Results and Discussion

### LCNF Fabrication

Nanofibers containing the six lignins were electrospun from a 0.5 M aqueous sodium hydroxide solution with 11 wt % PEO (Table 1), which was incorporated to increase the viscosity and hence spinnability, as described in previous work.^68^ Bead-free nanofibers were generated using electrospinning from Kraft pine/spruce, ionosolv eucalyptus, ionosolv spruce, and ionosolv miscanthus lignin using the standard formulation. However, the viscosities of the dopes for the organosolv beech and Kraft eucalyptus lignins were too low to yield a stable Taylor cone and hence uniform nanofiber mat, so the composition of the spinning solution was modified. In the case of organosolv beech lignin, increasing the molar weight of the PEO from 200 to 400 kDa was sufficient to increase the viscosity, maintaining the standard lignin/PEO ratio. For the Kraft eucalyptus lignin, using the PEO with a molar weight of 400 kDa still resulted in a low dope viscosity and poor fiber-formation. Therefore, the mass of PEO was doubled from 0.15 to 0.30 g instead, giving a higher proportion of PEO in the dope solution (20 wt % of the solid content). After the adjustments, the viscosity of the spinning dope solutions ranged from 0.27 to 6.01 Pa·s at a shear rate of 10/s (Figure S1 and Table S3). The shear rate was used for monitoring due to previous work reporting that aqueous PEO solutions experienced a shear rate of 5–10/s during electrospinning from a 21G needle with 1 mL/h flow rate.^69^

**Table 1:** Composition of Spinning Dopes Used for Different Lignin Types

lignin type	mass of lignin (g)	mass of PEO (g)	*M*_w_ of PEO (kDa)	mass of 0.5 M NaOH(aq) solution (g)
OS beech (hardwood)	1.2	0.15	400	7.8
Kraft eucalyptus (hardwood)	1.2	0.30	200	7.8
Kraft pine/spruce (softwood)	1.2	0.15	200	7.8
IS eucalyptus (hardwood)	1.2	0.15	200	7.8
IS spruce (softwood)	1.2	0.15	200	7.8
IS miscanthus (grass)	1.2	0.15	200	7.8

The as-spun nanofiber mats were stabilized in air at 250 °C to convert the material from a thermoplastic to a thermoset, as previously reported, which avoids melting (fiber fusing) during carbonization.^56^ A slow heating rate of 1 °C/min was required to maintain the integrity of the nanofiber mats. Previous work reported that the temperature at which the stabilization step is carried out determines the nature of the cross-linking reactions, affecting development of porosity.^52^ However, in this work only 250 °C was used for simplicity. The stabilized mats were then carbonized in a nitrogen atmosphere for 2 h. During the carbonization, the sodium hydroxide introduced by the dope solvent acts as a chemical activation agent, which is needed to introduce porosity into the lignin nanofibers. During carbonization, the hydroxide ions are converted into carbonate ions, and the sodium carbonate crystals remain on the surface of the fibers,^68^ which are removed by washing with 0.5 M HCl, yielding the nanofiber mats.

Initially, a series of organosolv beech-derived LNF mats were carbonized at temperatures of 700, 1000, and 1300 °C to determine the optimum carbonization temperature to use for the study. The LCNFs carbonized at 1000 °C showed the most consistent capacitive performance across a wide range of current densities, as well as good capacitance retention (Figure S2), so was chosen as the carbonization temperature to use across the sample set.

As expected, the LCNF mats consisted of randomly aligned fibers with cylindrical filaments (Figure 1), with the average diameters varying substantially between samples. Average nanofiber diameters for the LCNFs generated at 1000 °C were similar to those observed in previous studies of LCNFs,^43^ and ranged from 251 ± 47 nm for the Kraft eucalyptus-derived CNFs to 686 ± 92 nm for the ionosolv miscanthus-derived CNFs (Table S4).

**Figure 1:**
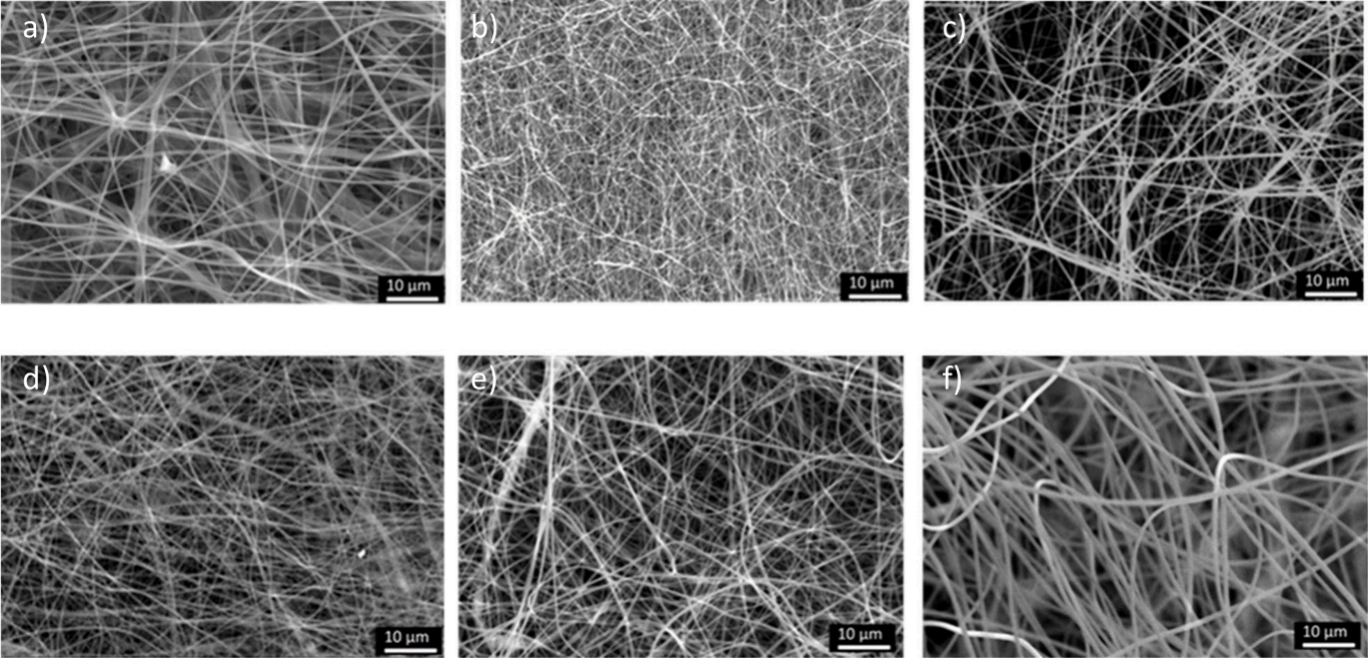
SEM images of (a) organosolv beech, (b) Kraft eucalyptus, (c) Kraft pine/spruce, (d) ionosolv eucalyptus, (e) ionosolv spruce, and (f) ionosolv miscanthus lignin-derived CNFs after carbonization at 1000 °C.

XPS analysis of the LCNFs generated at 1000 °C showed a high carbon content of 90–95%, which was expected for nanofibers after pyrolysis, with oxygen contents between 3 and 9% (Figure S3 and Table S5). A small amount of sulfur (0.5–1%) was retained in the LCNFs derived from Kraft and ionosolv lignins because of the sulfur-containing compounds used in the extraction (Na_2_S in the case of Kraft lignin and [DMBA]­[HSO_4_] or [DMBA]­[MeSO_3_] in the case of ionosolv lignins).

### Carbon Microstructure

XRD patterns of the six LCNF samples displayed broad diffraction peaks at 2θ values of ∼10°, 20°, and 35°, corresponding to the (002), (10), and (11) reflections of nongraphitic carbon structures, indicating a high level of structural disorder (Figure 2a), as expected for lignin polymer blends carbonized at 1000 °C.^70^ This is reflected in the relatively large interlayer spacings calculated from the XRD data through fitting (0.366–0.408 nm, Table S6), which is higher than that of turbostratic carbon (0.344 nm) or graphite (0.335 nm).

**Figure 2:**
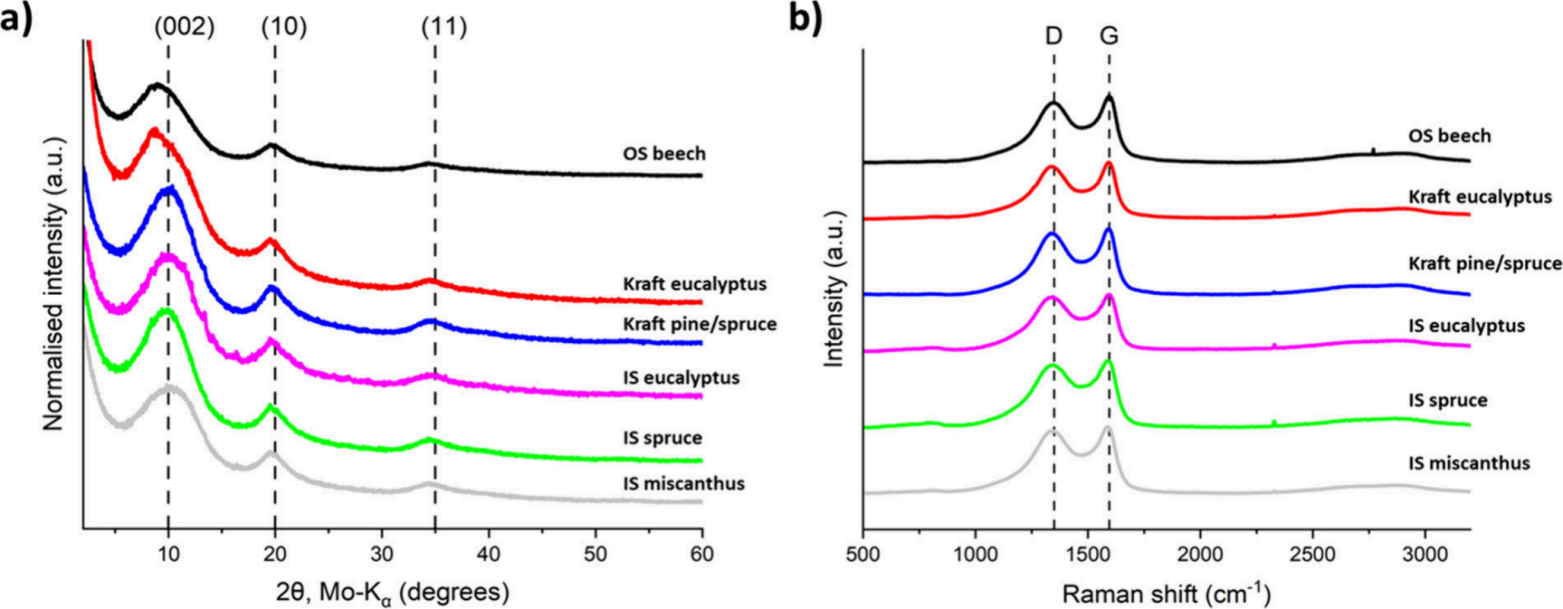
(a) XRD patterns and (b) averaged Raman spectra of LCNFs carbonized at 1000 °C.

The degree of structural ordering of the LCNFs was also assessed using Raman spectroscopy (Figure 2b), in which all LCNFs exhibited broad peaks at approximately 1340 and 1595 cm^–1^ in the first-order spectra, corresponding to the D and G bands, respectively. The G band arises from in-plane bond-stretching motion of sp^2^ hybridized carbon atoms and is present in all carbon materials containing sp^2^ sites, while the D band arises from graphene edge defects, indicating disorder within the carbon structure. A significant D band in the Raman spectra was consistent with the disordered structure of the carbons observed through XRD. Alongside the first-order spectra, low intensity second-order bands were observed at approximately 2500–3000 cm^–1^, characteristic of a carbon structure with limited long-range ordering. The intensity ratio of the D and G bands is commonly used as a quantitative indicator of the degree of structural ordering within a carbon material. Across the series of LCNFs, the peak intensity ratio (*I*_D_/*I*_G_) ranged from 1.21 to 1.46, indicative of a disordered carbon structure consisting of nanocrystalline graphitic domains.

The first-order Raman spectra were deconvoluted into four peaks using a model employing three Lorentzian and one Gaussian peak shape, based on a protocol validated by Sadezky et al. (Figure S4).^71^ Along with the D and G bands which were described by Lorentzian peak shapes, two additional peaks were included in the deconvolution and ascribed to the D3 and D4 bands. The D3 band at ∼1500 cm^–1^ is attributed to amorphous carbon^72^ and fitted using a Gaussian peak shape, whereas the D4 band at ∼1200 cm^–1^ is attributed to polyene structures and fitted with a Lorentzian line shape.^71^ The area ratios (*A*_D_/*A*_G_) ranged from 3.32 to 4.68, also indicative of nanocrystalline graphitic domains (Table S6).^73^ The XRD and Raman data suggest that different sources and extraction processes did not have a clear impact on the carbon structure after carbonization at 1000 °C.

### Porosity and Density of LCNFs

Porosity characteristics of the LCNFs (Figure 3a) were assessed over a wide range (0–10 nm) of pore sizes using a combination of N_2_ and CO_2_ sorption measurements. N_2_ sorption isotherms of the LCNFs (Figure S5b) most closely resembled a type I shaped isotherm, exhibiting a distinct region for micropore filling at low relative pressures, indicating a primarily microporous structure for all LCNF mats. However, the surface areas and pore volumes varied significantly between lignin types. The Kraft eucalyptus LCNF displayed the highest micropore volumes and surface areas in the series, contributing to a high BET surface area of 1721 m^2^/g (Table 2). On the other hand, the organosolv beech LCNF had a considerably lower BET surface area of 464 m^2^/g. The variation between lignin types was less pronounced in CO_2_ sorption measurements (Figure S5a), which are used to probe pore sizes that are inaccessible in N_2_ sorption measurements (below 1 nm). All lignin types displayed a high pore volume for pores with widths below 1 nm, indicating that the key difference between samples was in the larger micropore range (pore widths of 1–2 nm).

**Figure 3:**
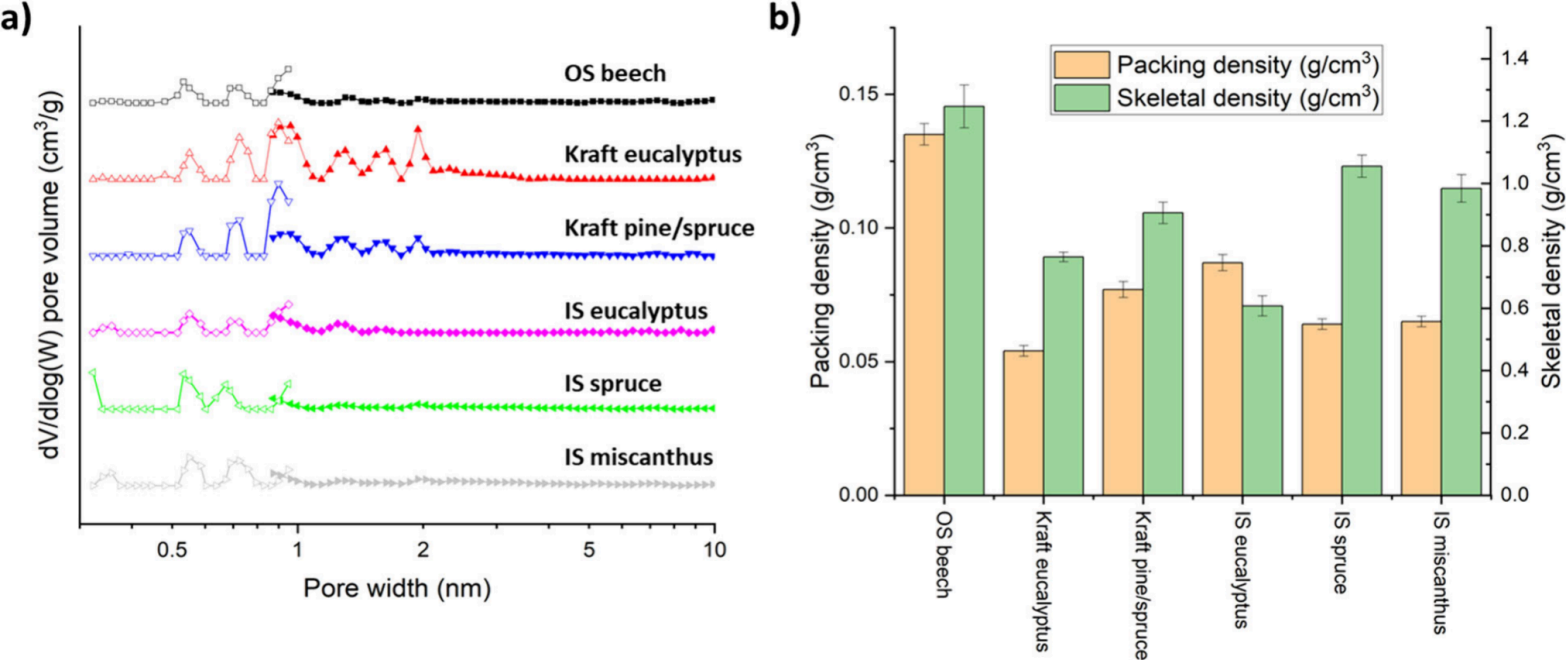
(a) Pore size distributions of LCNFs carbonized at 1000 °C. Unfilled symbols are derived from CO_2_ sorption and filled symbols from N_2_ sorption measurements. (b) Electronic conductivity values of LCNFs carbonized at 1000 °C.

**Table 2:** Summary of Values Calculated from N_2_ Sorption Isotherms at 77 K and CO_2_ Sorption Isotherms at 273 K

lignin type	total pore volume, N_2_ (cm^3^/g)	BET surface area, N_2_ (m^2^/g)	micropore volume, N_2_, *t*-plot (cm^3^/g)	DFT pore volume, N_2_ (cm^3^/g)	DFT surface area, N_2_ (m^2^/g)	DFT pore volume, CO_2_ (cm^3^/g)	DFT surface area, CO_2_ (m^2^/g)
OS beech	0.30	464	0.11	0.28	207	0.21	605
Kraft eucalyptus	0.86	1721	0.30	0.80	1057	0.38	1008
Kraft pine/spruce	0.47	861	0.17	0.43	518	0.39	1012
IS eucalyptus	0.33	704	0.23	0.31	228	0.18	523
IS spruce	0.44	905	0.25	0.42	179	0.26	973
IS miscanthus	0.52	985	0.24	0.49	245	0.23	772

The porosity of the LCNF mats was also assessed by calculating the density of the materials, using two different metrics. The packing (or bulk) density, which includes the volume of voids, was calculated using the mass and volume of a circular disk cut from the LCNF mats via eq 8:

8 $$\rho_{\mathrm{packing}}=m/tA\;\left(g/{\mathrm{cm}}^{3}\right)$$

in which *m* is the mass of the disk, *t* is the thickness, and *A* is the surface area. Also, the skeletal (or true) density was measured by helium pycnometry, which excludes the void volume between fibers and any accessible pores from the density. As expected for nanocarbon fiber networks, the packing densities were much lower (0.054–0.135 g/cm^3^) than the skeletal densities (∼0.61–1.25 g/cm^3^) (Figure 3b and Table S7). The volume packing fraction, derived by dividing the packing density by the skeletal density, ranged from 0.06 to 0.14 (Figure S7 and Table S7). A low volume packing fraction indicates that a large proportion of the volume of the LCNF mats (up to 94%) is occupied by free space, which is a general limitation of nondensified nanofiber electrodes.

### Conductivity

The electrical conductivity of the LCNFs was 7–19 S/cm (Figure 4 and Table S8), with an effect of the lignin origin apparent. The hardwood lignins (organosolv beech, Kraft eucalyptus and ionosolv eucalyptus) produced LCNFs with higher conductivities of 16–19 S/cm compared to the softwood and grass lignins (Kraft pine/spruce, ionosolv spruce, and ionosolv miscanthus), which displayed values of 7–10 S/cm.

**Figure 4:**
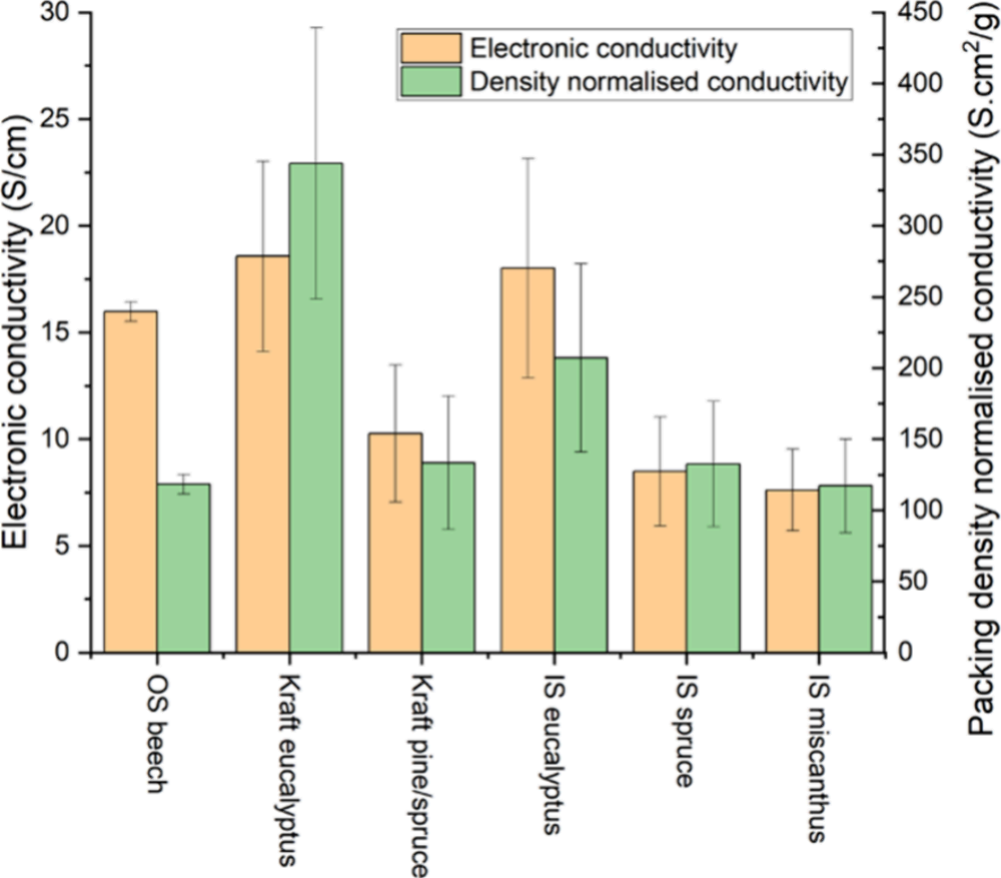
Electronic conductivity and packing density normalized electronic conductivity values of LCNFs after carbonization at 1000 °C, as measured by 4-point probe conductivity.

To account for the variations in density between the different samples and assess the intrinsic conductivity of the materials, the conductivity was also normalized by the packing density. After normalization, the eucalyptus LCNFs (Kraft and ionosolv eucalyptus) yielded 2–3 higher normalized conductivity values (344 and 207 S·cm^2^/g) compared to the rest of the samples (117–133 S·cm^2^/g). Since the organosolv beech LCNF mat only displayed a packing density normalized conductivity of 118 S·cm^2^/g, it is possible that the plant species and extraction method are more important for conductivity than the lignin type. The strength of correlation with capacitance is discussed later.

### Electrochemical Testing

Circular LCNF disks were used as electrodes in symmetric two-electrode Swagelok cells with 6 M KOH­(aq) as the electrolyte, with the CV showing a near rectangular shape, which is indicative of electric double layer charge storage. This shape was apparent at low scan rates of 5 mV/s (Figure 5a) and mostly retained at high scan rates of 500 mV/s (Figure 5b). At low scan rates (e.g., 5 mV/s), an increase in nominal capacitance was observed at around 1.2 V, indicating some electrolyte decomposition, which decreased upon increasing the scan rate. GCD traces also showed characteristic triangular profiles at both low (0.5 A/g) and high (100 A/g) current densities, with low IR drop, consistent with EDLC behavior (Figure 5d,e). EIS spectroscopy showed a cell resistance below 1 Ω for all LCNF electrodes, indicating good electrical contact between the freestanding electrodes and the current collectors (Figure 5c). All LCNFs showed good capacitance retention up to 10000 cycles, ranging from 82.8% for Kraft pine/spruce to 95.5% for organosolv beech (Figure 5f) as well as Coulombic efficiencies greater than 98% (Figure S8).

**Figure 5:**
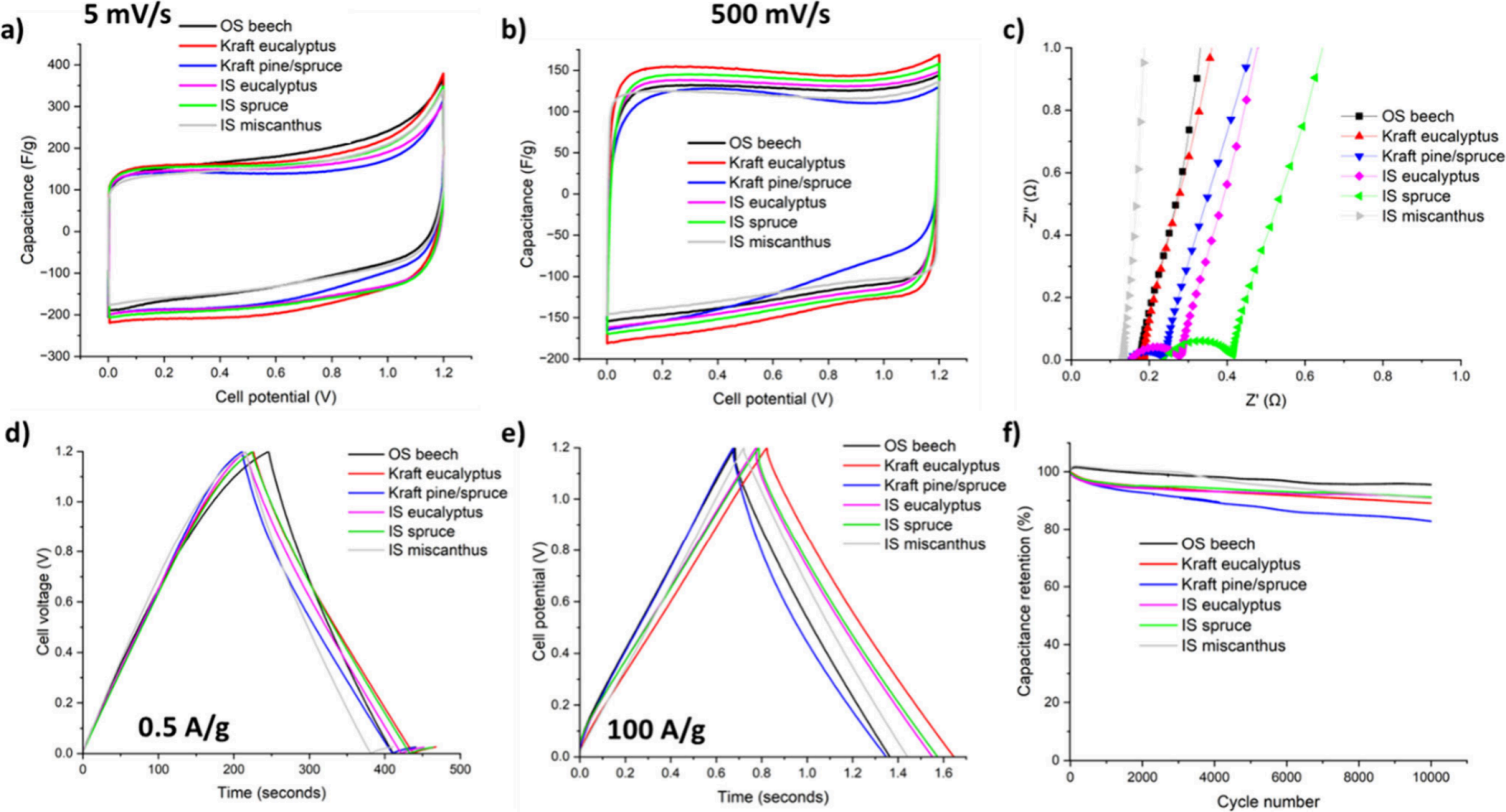
Electrochemical performance of the LCNFs in a two-electrode cell. CV curves at (a) 5 and (b) 500 mV/s. (c) Nyquist plot from the low-frequency region of the EIS spectra. GCD curves at current densities of (d) 0.5 and (e) 100 A/g. (f) Capacitance retention over 10,000 GCD cycles at 10 A/g.

Comparing the capacitive performance of the different lignins, the Kraft eucalyptus-derived LCNF displayed the highest gravimetric performance across the full range of current densities, with a maximum gravimetric capacitance of 192 F/g at 0.25 A/g (Figure 6a). It also maintained the highest capacitance of all the LCNFs at high current densities, with a capacitance of 134 F/g measured at 200 A/g. Gravimetric Ragone plots calculated from the GCD traces showed a maximum energy density of 8.9 Wh/kg and a power density of 52.7 kW/kg for the Kraft eucalyptus LCNF (Figure 6b). Commercial activated carbon powder-based supercapacitors typically offer energy densities of 5 to 15 Wh/kg and power densities of 1–100 kW/kg, so these results are competitive with the incumbent technology in terms of gravimetric performance.^74^

**Figure 6:**
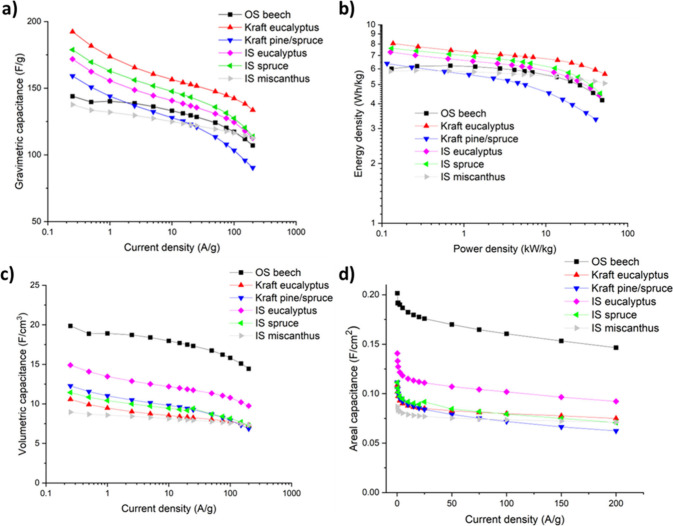
(a) Gravimetric capacitance as a function of current density, (b) gravimetric Ragone plot, (c) volumetric capacitance, and (d) areal capacitance as a function of the current density, calculated from GCD traces.

For practical application, the volumetric and areal capacitance are also crucial performance indicators. The volumetric performance is important in applications where the available space for energy storage devices is limited, such as in miniaturized devices or wearable electronics, because the void space with the electrode needs to be filled with electrolyte.^75^. The areal performance is important for minimizing parasitic mass within the cell assembly.^76^ The organosolv beech LCNF, displayed the highest volumetric and areal capacitance across all current densities tested with a maximum volumetric capacitance of 19.9 F/cm^3^ (Figure 6c) and maximum areal capacitance of 0.20 F/cm^2^ at 0.25 A/g (Figure 6d). Volumetric Ragone plots show a volumetric energy density of 0.81 Wh/L at low current density (Figure S9). This value is lower than most commercial and lab-scale porous carbons synthesized from bioresources (50–100 F/cm^3^, 1–3 Wh/L), so improvements need to be made such as densification.^53^

It is also noted that the electrochemical testing was carried out primarily to identify differences in carbon structure generated by the different lignin types; further testing in a pouch cell configuration would be required to assess the LCNF electrode’s ability as truly freestanding electrodes.

### Key Correlations between the Microstructure and Electrochemical Performance

Given the many characteristics surveyed, Pearson correlation coefficients were used to identify the morphological characteristics of the LCNFs that had the biggest influence on the gravimetric and volumetric capacitance of the LCNFs (Figure 7). Coefficients greater than +0.7 or smaller than −0.7 indicate a strong (positive or negative) linear correlation between the variables. A low and high current density (0.5 and 100 A/g) were chosen to assess the maximum capacitive performance of the LCNFs.

**Figure 7:**
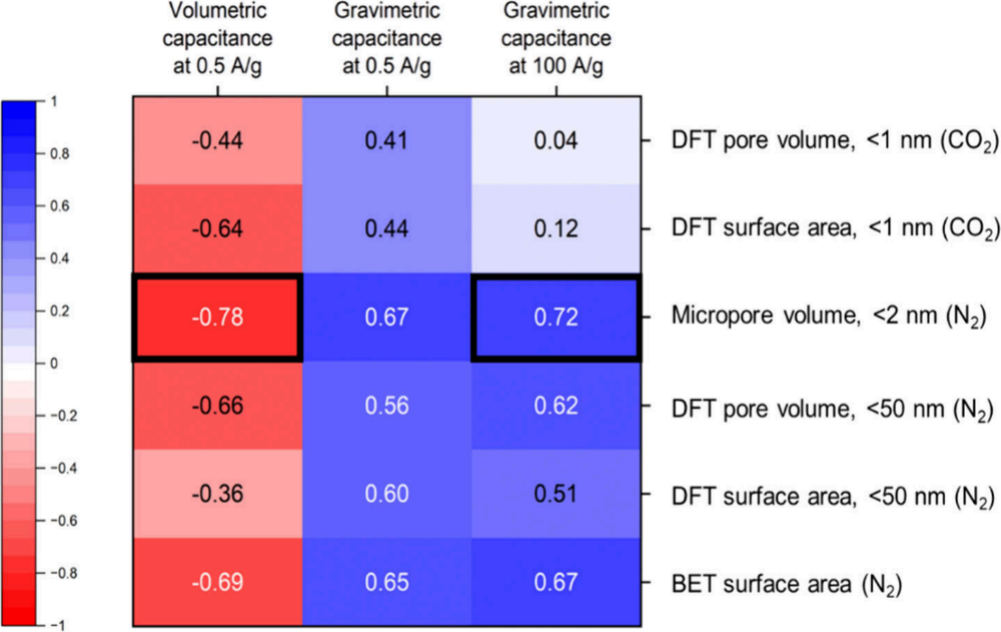
Heatmap indicating Pearson correlations between porosity characteristics of LCNF electrodes generated from different lignins and the gravimetric and volumetric capacitances at a current density of 0.5 A/g, and the gravimetric capacitance at 100 A/g. A Pearson coefficient ≥ 0.7 indicates a strong positive linear correlation, and a coefficient ≤ −0.7 indicates a strongly negative linear correlation. Strong correlations are indicated with a bold outline.

In this series of LCNFs, the porosity metrics had a strong impact on the gravimetric capacitance at both current densities, as commonly reported for carbon-based supercapacitors.^8–12^ Of the porosity metrics measured by nitrogen sorption measurements, the strongest correlation was observed with the micropore volume (in pores of <2 nm), particularly at the higher current density. Interestingly, only weaker correlations were observed for the DFT pore volume and surface area in ultramicropores (<1 nm) measured by CO_2_ sorption, likely due to limited ion accessibility. Hence, the presence of ultramicropores alone is not sufficient to give rise to high capacitance values. A large amount of micropores (pore widths between 1 and 2 nm) is crucial for facilitating access of the smaller pores during electrical double layer formation.^13,77^

A strong correlation between the densities of the LCNFs with the volumetric capacitance was observed, as expected (Figure 8). The impact of packing density on volumetric capacitance was higher, with a Pearson correlation coefficient of 0.95 compared to 0.58 for the correlation with skeletal density. Therefore, reducing the free space between the nanofibers, and increasing the volume packing fraction should have a greater impact on the volumetric performance of LCNF electrodes than the density of the fibers themselves. In addition, electronic conductivity normalized by packing density appeared to have a strong influence on the gravimetric capacitance, yielding correlation coefficients of 0.78 and 0.80 for current densities of 0.5 and 100 A/g, respectively.

**Figure 8:**
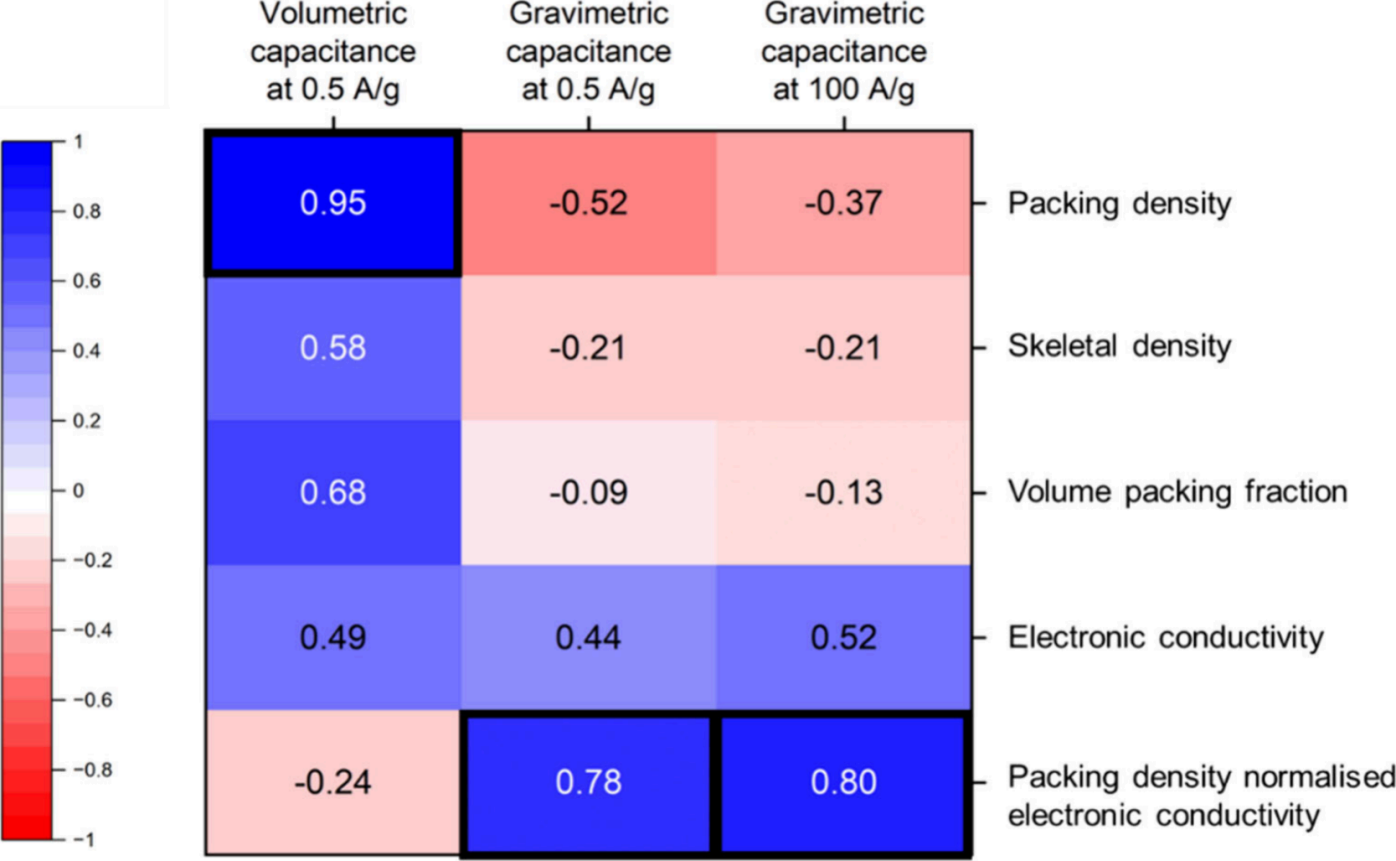
Heatmap indicating Pearson correlations between densities and electronic conductivities of the LCNFs and characteristics of the lignin precursor. A Pearson correlation coefficient ≥ 0.7 indicates a strong positive correlation, and a coefficient ≤ −0.7 indicates a strongly negative correlation. Strong correlations are indicated with a bold outline.

The carbon nanostructure characteristics derived from both XRD and Raman spectroscopy had a relatively limited influence on the capacitance (Figure S10). However, a correlation coefficient of −0.62 was observed between the interplanar spacing d_002_ and gravimetric capacitance at high current density, potentially suggesting that a more ordered carbon structure with narrower interlayer spacing may be beneficial at fast charging rates, potentially due to a higher electronic conductivity; however, the d_002_ value range was limited for this sample set. Therefore, the differences in porosity and density outweighed the relatively small differences in carbon nanostructure.

### Lignin Chemistry and Key Correlations

To determine if chemical characteristics of the lignins influenced the properties of the LCNF mats that were important for electrochemical performance, various aspects of the lignin structure (substructure composition, carbon–oxygen ratio, average molar weight, and molar weight dispersity) was quantified, and multivariate analysis was again used to identify correlations (Figure S11).

### Lignin Substructure Content

^1^H–^13^C HSQC NMR spectroscopy was used to identify structural differences between the lignins semiquantitatively where possible; the chemical structures of the identified motifs are shown in Figure S12. The aromatic region of the spectra showed the expected signals for the different lignin types. Signals corresponding to S and G subunits were detected in the hardwood lignins (Figures S13, S14, and S16 and Table S9), only G units were detected in the softwood lignins (Figures S15 and S17), while signals related to S, G, PCA, and “H” subunits were present in the spectrum for ionosolv miscanthus lignin, consistent with the structure of a technical grass lignin (Figure S18 and Table S9). All lignins showed signs of condensation (non-native substitutions of H on the benzene rings). The Kraft lignin spectra also contained peaks for carbohydrates, specifically xylose, with the Eucalyptus Kraft lignin having the strongest signals. Volume integration of these peaks relative to the combined aromatic peak volume suggests that carbohydrates are only present in a small proportion (2.7 and 1.1 per 100 aromatic units for Kraft eucalyptus and Kraft pine/spruce, respectively, Table S9), so they were not considered to be key contributors to the differences observed in carbon nanofiber structure and hence electrode performance.

It was previously reported that the nature of the aliphatic side chains is important for the development of porosity during carbonization.^52^ The linkages that can be clearly detected within HSQC NMR spectra are β-O-4′ ether (“C–O–C linkage”), resinol (β-β′) and phenylcoumaran (β-5) structures, which contain unhydrolyzable C–C bonds in addition to ether bonds (Figures S13–S18 and Table S10). Quantification using volume integration showed that lignins with a higher relative content of the C–O–C linkage yielded carbon materials with greater DFT pore volumes and surface areas (Figure S11). However, the correlation between the proportion of C–O–C linkages with the micropore volume and hence gravimetric capacitance only yielded a relatively weak Pearson correlation coefficient of 0.32 (Figure S19), suggesting that the porosity resulting from the breaking of ether linkages occurs across a range of length scales and is not localized to the micropore range.

### Hydroxyl Group Content

The total number and type of the hydroxyl groups in the lignin structure have been previously related to porosity of the LCNFs,^52^ hence the hydroxyl group content was assessed using quantitative ^31^P NMR spectroscopy. A range of typical hydroxyl environments were identified, including aliphatic and carboxylic hydroxyl groups, as well as phenolic hydroxyl groups which belong to S, G, or H (Figures S20–S25). The total hydroxyl content of the lignins varied between 4.16 and 6.70 mmol/g, with the highest hydroxyl content found in the ionosolv miscanthus and Kraft eucalyptus lignins, with total hydroxyl contents of 6.70 and 6.00 mmol/g, respectively (Table S11). The distribution of hydroxyl functional groups also varied between the lignin types. Comparing the two Kraft lignins, for example, the Kraft pine/spruce lignin sample contained fewer phenolic hydroxyl groups (Tables S11 and S12).

The correlation map suggests that the total number of hydroxyl groups had a strong positive correlation with the porosity metrics, particularly the micropore volumes, of the LCNFs (Figure 9a, S11, and S26a). A stronger correlation between the phenolic hydroxyl groups with the micropore volume (Figure 9b and S27a) was observed than with aliphatic OH group content (Figure S11). Similarly strong correlations were observed for the total hydroxyl and phenolic hydroxyl group contents and the gravimetric capacitance (Figures 9a, S26b, 9b, and S27b). Although beneficial for the generation of microporosity, a higher hydroxyl group content had a strongly negative correlation with the viscosity and hence packing density of the final LCNFs (Figures 9c, S11, and S28), affecting the volumetric performance of the resulting free-standing electrode. The strong correlation between dope viscosity and packing density of the fiber material (Figures 9d, S11, and S29) suggests that volumetric performance can be increased through optimization of the spinning solution viscosity.

**Figure 9:**
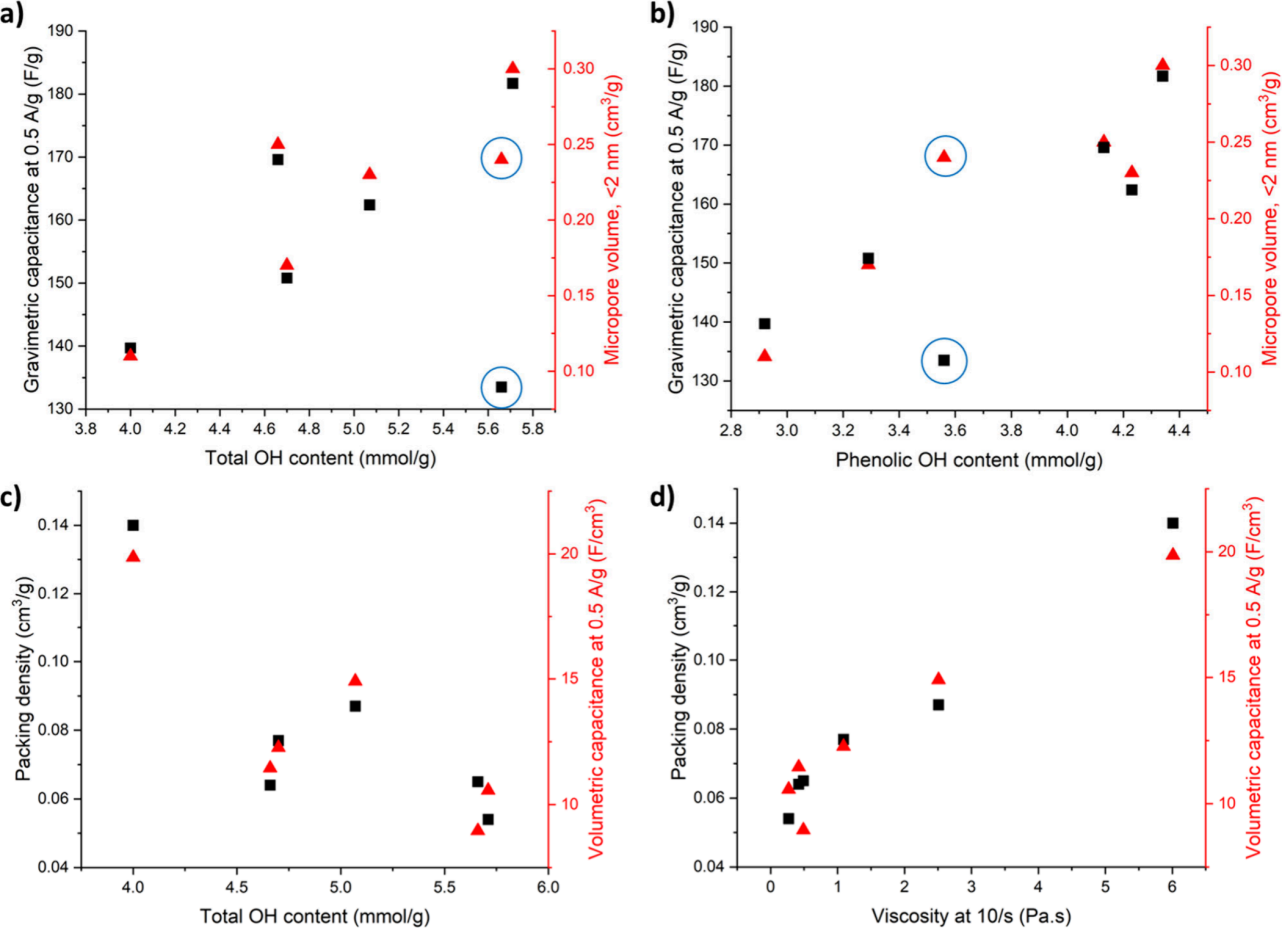
Plots of (a) total hydroxyl content and (b) phenolic hydroxyl content of lignins (measured by ^31^P NMR spectroscopy) versus gravimetric capacitance of single LCNF electrodes at 0.5 A/g and micropore volume of LCNFs. Data points for ionosolv miscanthus LCNF are circled in blue as outliers. Plots of (c) the total hydroxyl content of lignins and (d) the viscosity of the resulting spinning dope (solution composition in Table 1) versus the packing density of subsequent LCNF electrodes and the volumetric capacitance of single LCNF electrodes at 0.5 A/g. Pseudo-single electrode capacitances were measured.

It should be noted that the ionosolv miscanthus LCNFs performed more poorly than was expected from its hydroxyl group or micropore content (Figure 9). The ionosolv miscanthus LCNF displayed the lowest electronic conductivity within the sample set, both measured and normalized by packing density (Figure 4 and Table S8), which may limit its electrochemical performance.

### Molar Weight and Ash Content

The molar weight distributions of the different lignins were assessed using GPC (Figure S30 and Table S13). The two softwood lignins displayed the highest number-average molecular weights (Figure S30 and Table S13), contributing to a strongly negative correlation with the S-to-G ratio (Figure S11); the softwood lignins also had higher carbon-to-oxygen ratios (Table S14), suggesting a more condensed lignin structure. There was generally a weak correlation between molar weight and microstructural characteristics of the LNCFs in this study (Figure S11). The lignins used in this study had a low ash content (1–3%) as measured by TGA in air (Table S14). As the ash contents of the lignins were similar and generally low, they were not used in the correlation map.

### Carbon Fiber Electrode Yield

TGA of the powdered lignins in nitrogen was used to estimate the carbon yield (Figure S31), with the assumption that the LCNFs would follow the same trend. The ionosolv lignins had the highest char yields when comparing different extraction methods (≥40%), and the eucalyptus lignins had the highest char yield when comparing different biomass feedstock types. The correlation map indicates that the presence of ether linkages in the lignin, measured by TGA reduced the overall char yield (Figure S11 and Table S15) which is an important factor for the cost of producing the nanofiber electrodes.^29^

### Engineering of Spinning Dope Composition

It was noted that packing density and hence volumetric capacitance was strongly correlated with solution viscosity (Figure S32a,b). Hence, it was attempted to optimize microstructural characteristics and hence capacitance, by preparing an additional electrode with organosolv beech lignin, which had displayed the highest volumetric capacitance and highest spinning dope viscosity. A reduced solid content of 12% (lignin + PEO) compared to 15% (Table S16) was chosen as the optimization parameter.

Reducing the solid loading to 12% resulted in a decrease in dope viscosity from 6.01 Pa·s to 0.16 Pa·s, as expected (Table S16 and Figure S32). The lower viscosity solution was spinnable and generated carbon nanofibers with a smaller average fiber diameter (422 nm compared to 613 nm) after carbonization at 1000 °C (Figure S33). In addition, the pore volume and surface areas significantly increased across a range of pore widths (Figure S34 and Table S17), which resulted in an increased gravimetric capacitance; the increased pore volume may be ascribed to the increased ratio of activating agent (NaOH) to lignin/PEO. The additional porosity also impacted the density characteristics of the LCNFs, with a reduction in packing density from 0.135 to 0.102 g/cm^3^ and the skeletal density from 1.25 to 0.71 g/cm^3^ (Table S18); since the skeletal density decreased more, the volume packing fraction increased slightly from 0.11 to 0.14.

In the supercapacitor application, the lower loading organosolv LCNF showed similar pseudorectangular shapes in the CV tests, indicating primarily EDLC behavior and high Coulombic efficiencies (Figures S35 and S36). An increase in the gravimetric capacitance using the lower solid loading was observed (Figure S37a), but it was associated with a decrease in the volumetric and areal capacitance (Figure S37b,c) due to the reduced packing density. The sample prepared with 12% solid loading was added to master plots of LCNF micropore volumes versus gravimetric capacitance and packing densities versus volumetric capacitance at 0.5 A/g (Figure 10). While the reduction in packing density correlated well with the volumetric capacitance, the gravimetric capacitance was lower than would be expected when simply considering the micropore volume of the LCNF. The reason for this is unclear.

**Figure 10:**
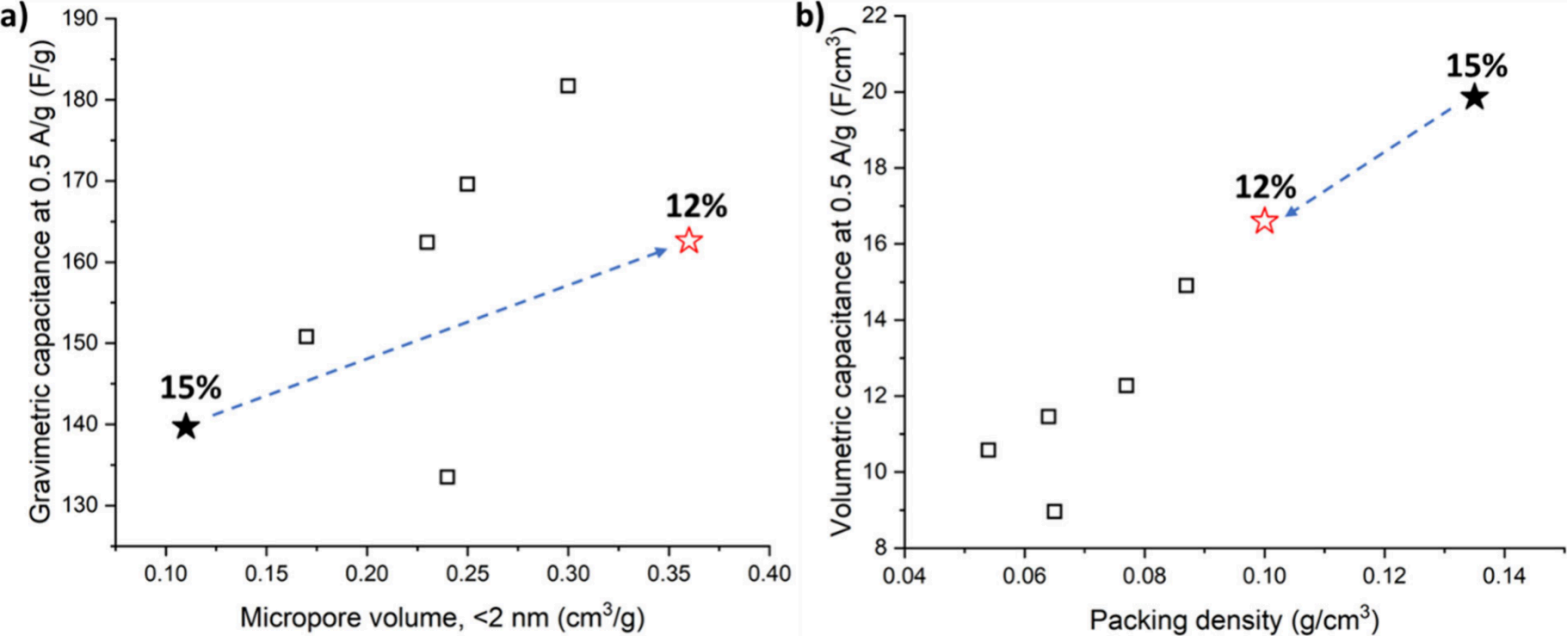
Plots of (a) micropore volume versus gravimetric capacitance at 0.5 A/g and (b) packing density versus volumetric capacitance at 0.5 A/g. Black/filled stars correspond to organosolv beech LCNF prepared using solution with 15% solid loading and red/unfilled stars correspond to organosolv beech LCNF prepared using solution with 12% solid loading. Pseudo-single electrode capacitances were measured.

The results highlight the potential of boosting activation as a means of increasing gravimetric performance, albeit at the expense of volumetric performance in this case. Considering the volumetric performance, maximizing the solid loading in the spinning solution appears to be a straightforward means of improving the packing density of the resulting LCNF electrodes. However, the maximum achievable improvement in volumetric performance before the viscosity is excessively high, and the solution is no longer electrospinnable, is likely to be relatively limited. Hence, optimization of carbon fiber mats needs to be coupled with engineering approaches such as increased fiber alignment or mechanical pressing to practically reach commercially relevant volumetric performances.

### Electrolyte Engineering

As discussed in the Introduction, a means of boosting the energy density of a supercapacitor cell is through selection of the electrolyte to widen the operational voltage window of the device. An example of a low-cost WIS electrolyte that has been reported in the literature is 12 mol/kg NaNO_3_.^78^ To explore the potential benefit of using a WIS electrolyte in combination with LCNF electrodes, a symmetric supercapacitor cell was constructed using the Kraft eucalyptus LCNF, as it displayed the highest gravimetric performance in 6 M KOH. Using 12 mol/kg NaNO_3_, the operating voltage window could indeed be increased from 1.2 to 1.8 V while maintaining a near rectangular-shaped cyclic voltammogram (Figure 11a,b), although again with some electrolyte decomposition close to the maximum potential at lower scan rates. Triangular GCD curves were also observed with low IR drop, indicating the expected EDLC behavior (Figure 11d,e).

**Figure 11:**
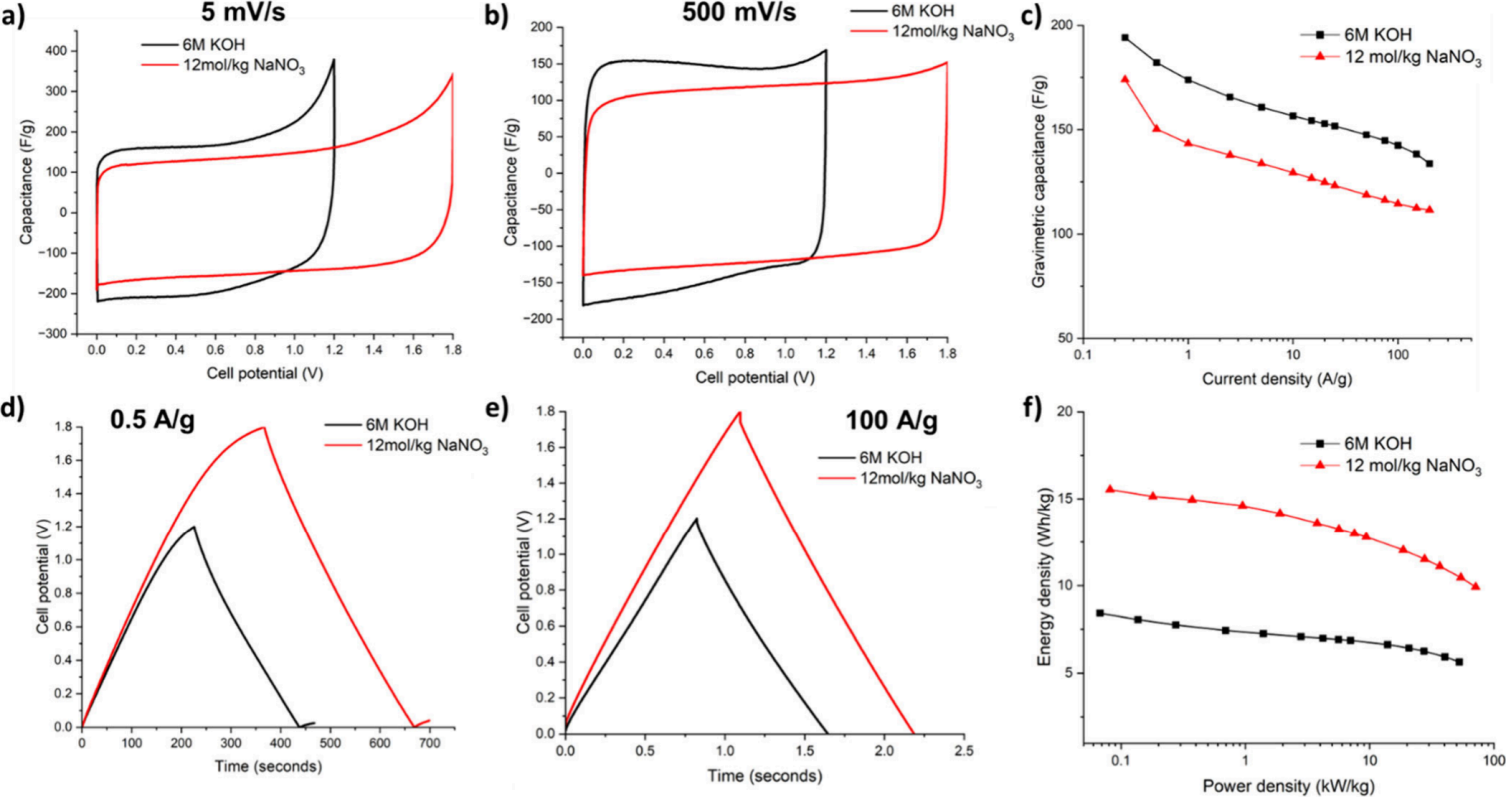
Electrochemical performance of Kraft eucalyptus LCNF in 6 M KOH and 12 mol/kg NaNO_3_ electrolytes. CV curves at (a) 5 and (b) 500 mV/s. (c) Gravimetric capacitance as a function of the current density. GCD curves at current densities of (d) 0.5 and (e) 100 A/g. f) Ragone plots calculated from GCD traces.

Interestingly, using the WIS electrolyte resulted in a drop in gravimetric capacitance, potentially due to its lower ionic conductivity or reduced accessibility to the small pores. A maximum pseudo-single capacitance of 174 F/g at 0.25 A/g was achieved for 12 mol/kg NaNO_3_ compared to 192 F/g for 6 M KOH (Figure 11c). However, the wider voltage window resulted in a significant increase in energy density from 8.9 Wh/kg with 6 M KOH to 15.5 Wh/kg (Figure 11f). While a high Coulombic efficiency of above 98% was maintained over 10000 cycles (Figure S39b), the capacitance retention was lower in the case of 12 mol/kg NaNO_3_, with only 54% retention compared to 89% for 6 M KOH (Figure S39a). EIS spectra also showed an increase in the charge-transfer resistance after 10000 cycles when the WIS electrolyte was used (Figure S40). Previous works have achieved capacitance retentions of 90% with this electrolyte after 9000 cycles using commercial YP-50F activated carbon^78^ and 90.3% over 10000 cycles for a coal-derived porous carbon,^79^ both at a current density of 5 A/g. Therefore, improving long-term performance requires a fundamental understanding of decomposition pathways and the interactions between LCNFs and the 12 mol/kg NaNO_3_ electrolyte, followed by optimization.

### Qualitative Sustainability Evaluation

Finally, the overall sustainability of the proposed use of LCNFs as freestanding supercapacitor electrodes is discussed qualitatively. There is currently a lack of studies in the literature in which technoeconomic and life-cycle assessment is used to quantitatively compare the merits of freestanding electrode architectures with those of conventional powder-based electrodes. However, existing data on electrospun fiber production (limited to lab-scale processes) show sensitivities to electricity consumption during electrospinning (related to the size of the applied voltage) and membrane yield.^80,81^ The choice of feedstock for activated carbon preparation, as well as the carbonization procedure can significantly influence the GWP of the fabrication process.^82,83^ In addition, the chemical activation method has a substantial impact, with steam activation having generally a lower environmental impact than activation with alkali metal hydroxide solutions;^82^ in this context, recycling of the chemical activation agent appears important. A renewable feedstock (coconut husk) has shown reduced impact in some midpoint categories compared to a coal-based electrode material but not others.^83^ It has also been shown that fluorinated binders contribute significantly to environmental impact, suggesting that binder free architectures are desirable.^83^ However, differences in systems boundaries and choice of functional unit (e.g., active material mass, electrode mass, capacitance or stored energy) make comparisons across studies challenging.^82^ Therefore, consistent assessment of the environmental impacts of lignin electrode materials, considering device architecture and electrolyte selection, is needed to lay out clearly the benefits (or limitations) of porous LCNFs in supercapacitor applications.

## Conclusions

In this study, a series of electrospun LCNFs were fabricated from six lignin feedstocks covering a range of botanical origins and extraction methods, representing the largest sample set to date, including three ionosolv lignins for the first time. The chemical structure of the lignin feedstock influenced the microporosity and carbon fiber yield, with the relative abundance of different aliphatic linkages and the hydroxyl content playing a key role in porosity development, which in turn was the domintant influence on gravimetric capacitance. Kraft eucalyptus LCNFs achieved the highest gravimetric performance 192 F/g at 0.25 A/g) attributed to a high proportion of β-O-4′ ether linkages and phenolic hydroxyl groups in the precursor lignin.

For the first time, correlations between spinning dope and lignin characteristics with volumetric capacitance were established. In particular, spinning dope viscosity was found to strongly influence LCNF packing density and hence volumetric electrochemical performance. The organosolv beech dope solution exhibited the highest viscosity and produced LCNFs with the highest volumetric capacitance (19.9 F/cm^3^ at 0.25 A/g), despite relatively poorly developed microporosity. Overall, a trade-off was observed between the micropore volume and packing density and thus between gravimetric and volumetric capacitance across this sample set.

Carbon structure characteristics were only weakly correlated with performance, possibly due to the limited differences observed in the disordered carbons obtained in this study, although a moderate influence of the interlayer spacing on gravimetric performance was observed at higher current densities. Related to this, low conductivity may explain the unexpected underperformance of the ionosolv Miscanthus lignin derived LNCFs. Overall, the use of the cost-effective ionosolv lignins yielded promising results, particularly in terms of carbon yield, and their use in this and similar applications should be explored further.

When using 6 M KOH as the electrolyte (8.9 Wh/kg), the highest gravimetric energy density achieved in this study is comparable with commercial activated carbon-based electrodes (5–15 Wh/kg). However, the maximum volumetric energy density (0.81 Wh/L) was considerably lower than commercial and lab-scale porous carbons derived from bioresources (1–3 Wh/L), primarily due to the low packing fraction of around 0.1 or 10% in the LCNFs. The use of a WIS electrolyte to widen the operational voltage window of supercapacitor devices increased the gravimetric energy density from 8.9 Wh/kg to 15.5 Wh/kg. However, further work is required to understand the charge storage mechanisms in WIS electrolytes and their interactions with the LCNFs to improve long-term cyclability.

Overall, the results suggest that lignin selection enables reasonable gravimetric performance in LCNF electrodes but may not be sufficient to achieve competitive volumetric performance, even when dope viscosity is maximized. Therefore, standard electrospun carbon nanofiber preparation procedures are likely to require additional means of densification, such as mechanical pressing.

Although this work was carried out on the widest range of lignin types reported to date, including additional lignin sourcesand a broader range of stabilization and carbonization temperatures would improve statistical robustness and help validate the observed correlations. Investigating lignins extracted from the same biomass source using the same extraction method but processed under different extraction severities would isolate variations arising from the feedstock. Uncertainty remains regarding the role of the fiber spinning aid PEO, particularly with respect to variations in solid content and molar weight. Further electrochemical testing in pouch cell configurations would enable a more realistic assessment of LCNFs as freestanding electrodes. Finally, future work should incorporate life-cycle assessment and technoeconomic analysis to evaluate the sustainability and cost of LCNF-based supercapacitor devices.

## Supplementary Material



## Notes

Comparison with literature results, details of electrodes, viscosity measurements, electrochemical measurements, XPS spectra, Raman spectra, CO_2_ and N_2_ sorption isotherms, density values, Pearson correlation plots, electronic conductivity values, HSQC NMR spectra, ^31^P NMR spectra, GPC data, TGA curves, and SEM images (PDF)
