# Supplementary material for: Lignin Selection Improves the Performance of Porous Carbon Nanofiber Electrodes in Freestanding Supercapacitors
Source: ACS Sustain Chem Eng. 2026 Jun 1;14(23):10394–410. doi: 10.1021/acssuschemeng.6c00388 (PMC13273807; doi:10.1021/acssuschemeng.6c00388)
Supplement: Supplementary file 1 [file sc6c00388_si_001.pdf]

Electronic Supporting Information

Lignin Selection Improves the Performance of Porous Carbon Nanofiber Electrodes in Freestanding Supercapacitors

Robert D. Hunter,[a,b] Sarah Seidner,[a] Caitlin Brooker-Davis,[c] Milo S. P. Shaffer,[b,d] Agnieszka Brandt-Talbot[b*] and Maria-Magdalena Titirici[a,e*]

[a]Department of Chemical Engineering, Imperial College London, London, SW7 2AZ, UK
[b]Department of Chemistry, Imperial College London, London, W12 0BZ, UK
[c]School of Chemistry, University of Birmingham, Edgbaston, B15 2TT, UK
[d]Department of Materials, Imperial College London, South Kensington Campus, London SW7 2AZ, UK
[e]Advanced Institute for Materials Research (WPI-AIMR), Tohoku University, 2-1-1 Katahira, Aobaku, Sendai, Miyagi, 980-8577, Japan

*Corresponding authors:
Agnieszka Brandt-Talbot: a.brandt-talbot@imperial.ac.uk
Magda Titirici: m.titirici@imperial.ac.uk

Number of pages: 29
Number of Figures: 40 (Figures S1-S40)
Number of Tables: 18 (Tables S1-S18)

Table S1: Reported electrospun carbon nanofibre materials tested as supercapacitors

Materials	Solvent	Thermal treatment	Electrolyte / no. electrodes	Gravimetric capacitance (single electrode) (F/g)	Volumetric (F/cm^3) or areal (F/cm^2) capacitance	Ref.
Coal/PAN/β-cyclodextrin/PMMA	DMF	280 °C (air) / 800 °C (N_2)	6 M KOH / 3	262 at 1 A/g	n.d	1
PAN/PMMA	DMF	800 °C (N_2) / mix with melamine, 800 °C (N_2) / mix with pyrrole + $FeCl_3.6H_2O$, 80 °C	6 M KOH / 3	342.13 at 1 A/g	n.d.	2
Bituminous coal/PAN	DMF	800 °C (N_2)	6 M KOH / 3	191.2 at 1 A/g	n.d.	3
PAN/PVB/PVP/$SnCl_2.2H_2O$	DMF	280 °C (air) / 800 °C (N_2)	6 M KOH / 3	342.05 at 0.5 A/g	n.d.	4
PEI/PAN	DMF	250 °C (air) / 800 °C (N_2)	6 M KOH / 3<br><br>6 M KOH / 2	200 at 1 A/g<br><br>160.4 at 0.5 A/g	n.d.	5
Coal/PAN/$Zn(ac)_2.2H_2O$	DMF	280 °C (air) / 950 °C (N_2)	6 M KOH / 3	252 at 1 A/g	n.d.	6
PAN	DMF	250 °C (air) / soak in KOH, 800 °C (N_2)	6 M KOH / 3	461.2 at 0.5 A/g	n.d.	7
PAN/PMMA	DMF	280 °C (air) / 1050 °C (N_2), introduce NH_3	6 M KOH / 3	208 at 1 A/g	n.d.	8
PAN/PVP	DMF	280 °C (air) / 800 °C (N_2), introduce NH_3	6 M KOH / 3	197 at 0.2 A/g	n.d.	9
PAN/PMMA	DMF	280 °C (air) / 800 °C (N_2)	6 M KOH / 3	192 at 0.5 A/g	n.d.	10
PAN/PVP	DMF	300 °C (air) / 850 °C (N_2)	6 M KOH / 3<br><br>6 M KOH / 2	198 at 1 A/g<br><br>148 at 1 A/g	n.d.	11
PAN/PMMA	DMF	270 °C (air) / 1100 °C (N_2) / soak in KOH, 1000 °C (N_2)	6 M KOH / 2	191 (from CV at 5 mV/s)	n.d.	12
PAN/H_3PO_4	DMF	280 °C (air) / 800 °C (N_2)	6 M KOH / 2	156 at 0.5 A/g	n.d.	13
PAN/PVP/poly(styrene-co-acrylonitrile)	DMF	250 °C (air) / 800 °C (N_2)	2 M KOH / 3	152 at 1 A/g	158 F/cm^3 at 1 A/g (material pressed at 10 MPa before testing)	14
Squid ink/PAN	DMF	260 °C (air) / add KOH, 800 °C (Ar)	6 M KOH / 3	422.7 at 1 A/g	n.d.	15
Alkali lignin/PAN	DMF	280 °C (air) / 1000 °C (N_2)	1 M H_2SO_4 / 3	137 at 0.5 A/g	n.d.	16
Alkali lignin/PVP/$Zn(NO_3)_2$	DMF	150 °C (air) / 350 °C (air) / 800 °C (N_2)	6 M KOH / 3	289 at 0.1 A/g	n.d.	17
Alkali lignin/PVA	H_2O	220 °C (air) / 1000 °C (N_2)	6 M KOH / 2	241.4 (from CV at 5 mV/s)	n.d.	18
Alkali lignin/PVA	H_2O	180 °C (air) / 220 °C (air) / 1200 °C (Ar)	6 M KOH / 2	64 at 0.5 A/g	n.d.	19
Softwood Kraft lignin	DMF	250 °C (air) / 1000 °C (N_2) / Plasma treatment	6 M KOH / 2	103 at 0.25 A/g	n.d.	20

Organosolv beech lignin/PEO	0.5 M NaOH	800 °C (N_2)	6 M KOH / 2	180 at 0.1 A/g	n.d.	21
Organosolv beech lignin/PEO	0.5 M NaOH	200 °C (air) / 800 °C (N_2)	6 M KOH / 2	210 at 0.1 A/g	n.d.	22
Organosolv hardwood lignin/PEO	0.5 M NaOH	200 °C (air) / 800 °C (N_2)	6 M KOH / 2	200 at 0.1 A/g	130 F/cm^3 at 0.1 A/g (after densification pre-carbonisation)	23
Kraft eucalyptus lignin	DMF	250 °C (air) / 900 °C (N_2) / introduce CO_2 at 800 °C	6 M KOH / 2	155 at 0.1 A/g	0.225 F/cm^3 at 0.1 A/g	24
Kraft eucalyptus lignin	DMF	310 °C (air) / 800 °C (N_2)	6 M KOH / 2	164 at 0.1 A/g	n.d.	25
Kraft pine/spruce lignin	DMF	340 °C (air) / 800 °C (N_2)	6 M KOH / 2	150 at 0.1 A/g	n.d.	25
Kraft eucalyptus lignin/PEO/$NaNO_3$	1 M NaOH	800 °C (5% H_2 in N_2)	6 M KOH / 2	192 at 0.1 A/g	0.350 F/cm^2 at 0.1 A/g	26

Table S2: Details of electrode masses and mass loadings used in all Swagelok cells.

Lignin type	Carbonisation temperature (°C)	Electrolyte used	Electrode mass in cell 1 (mg)	Electrode mass in cell 2 (mg)	Mass loading (mg/cm^2)
**Organosolv beech**	700	6 M KOH	1.41	1.40	1.2
	1000	6 M KOH	1.55	1.50	1.4
	1000 (12% solid loading)	6 M KOH	1.17	1.13	1.0
	1300	6 M KOH	0.74	0.70	0.6
**Kraft eucalyptus**	1000	6 M KOH	0.63	0.60	0.5
	1000	12 mol/kg $NaNO_3$	0.51	0.62	0.7
**Kraft pine/spruce**	1000	6 M KOH	0.79	0.94	0.8
**Ionosolv eucalyptus**	1000	6 M KOH	0.93	1.03	0.9
**Ionosolv spruce**	1000	6 M KOH	0.70	0.74	0.6
**Ionosolv miscanthus**	1000	6 M KOH	0.71	0.76	0.7

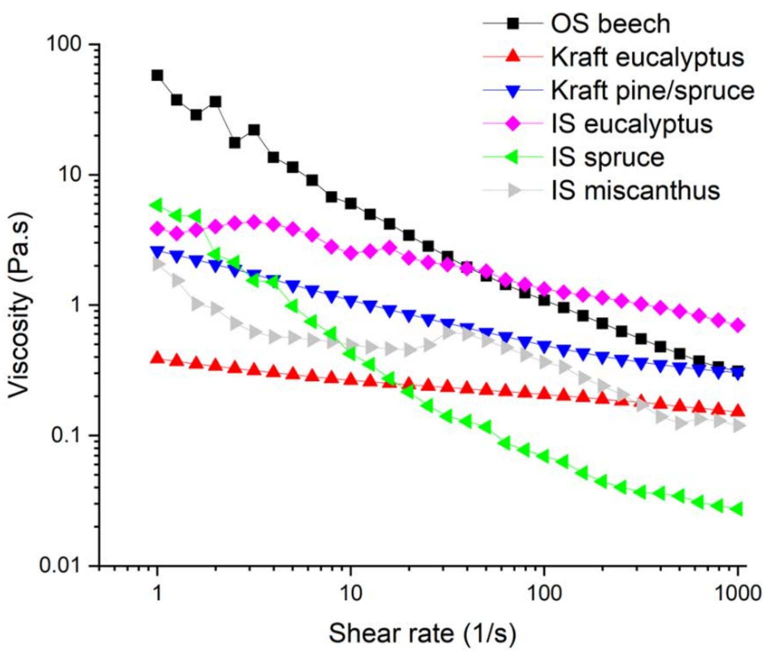

Figure S1: Viscosity of the lignin/PEO/NaOH spinning dopes listed in Table 1 at shear rates of 1-1000/s.

Table S3: Viscosity of aqueous lignin/PEO/NaOH spinning dopes at a shear rate of 10/s.

**Lignin type**	**Viscosity at 10/s (Pa.s)**
OS beech	6.01
Kraft eucalyptus	0.27
Kraft pine/spruce	1.09
IS eucalyptus	2.51
IS spruce	0.42
IS miscanthus	0.49

Table S4: Diameter of LCNFs after carbonization at 1000 °C.

**Lignin type**	**Average fibre diameter (nm)**
OS beech	613 ± 56
Kraft eucalyptus	251 ± 47
Kraft pine/spruce	488 ± 57
IS eucalyptus	352 ± 51
IS spruce	496 ± 78
IS miscanthus	686 ± 92

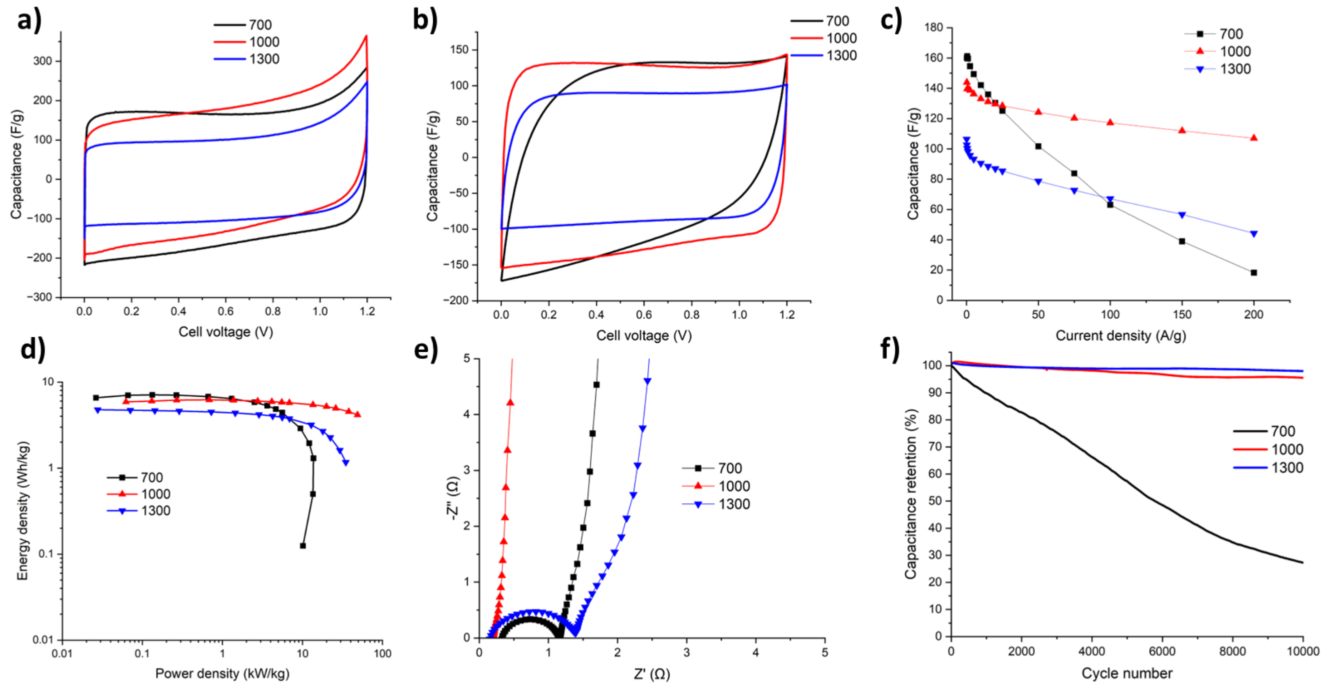

Figure S2: Electrochemical performance of organosolv beech-derived LCNFs carbonized at 700, 1000 and 1300 °C in a two-electrode cell. CV curves at a) 5 mV/s and b) 500 mV/s, c) gravimetric capacitance as a function of current density, d) Ragone plot, e) Nyquist plot from low frequency region of the EIS spectra and f) capacitance retention over 10,000 GCD cycles at 10 A/g.

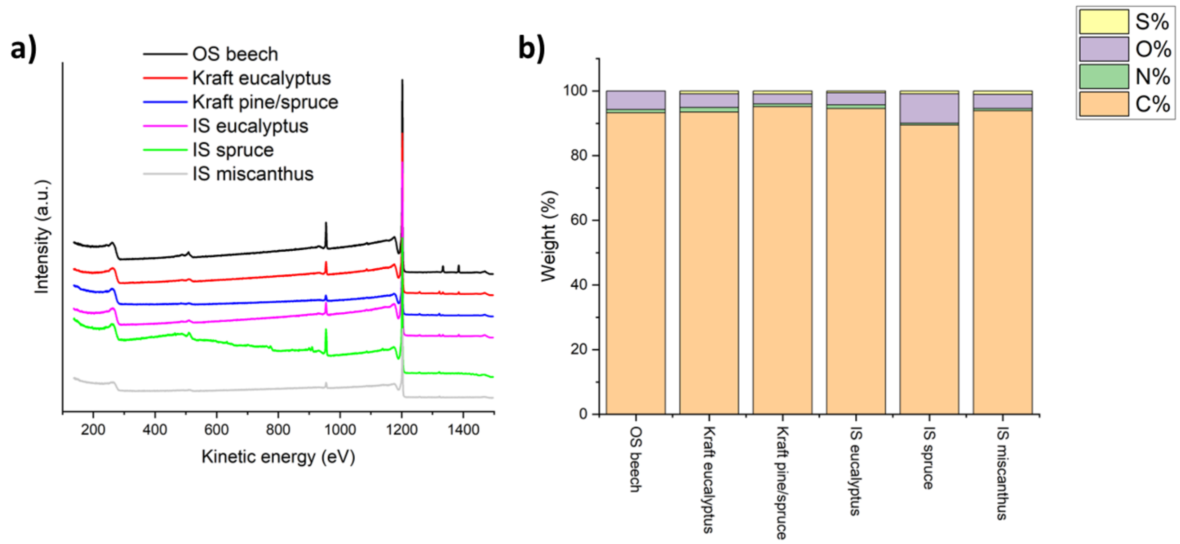

Figure S3: a) XPS survey spectra and b) elemental composition of LCNFs carbonised at 1000 °C.

Table S5: Elemental composition of LCNFs carbonised at 1000 °C, from XPS.

Lignin type	C%	N%	S%	O%
OS beech	93.28	1.01	<0.01	5.71
Kraft eucalyptus	93.47	1.42	0.84	4.26
Kraft pine/spruce	95.13	0.89	0.91	3.07
IS eucalyptus	94.60	1.09	0.57	3.74
IS spruce	89.47	0.57	0.89	9.06
IS miscanthus	93.92	0.69	1.04	4.35

Table S6: Parameters extracted from fitting of XRD data in Fig. 2a and deconvolution of Raman spectra in figure S4.

**Lignin type**	**d_{002} (nm)**	**L_c (nm)**	**I_D/I_G**	**A_D/A_G**	**G peak position (cm^{-1})**	**G FWHM (cm^{-1})**	**D peak position (cm^{-1})**	**D FWHM (cm^{-1})**
OS beech	0.389	1.77	1.21 ± 0.02	3.32 ± 0.12	1597 ± 1	65 ± 1	1345 ± 1	179 ± 3
Kraft eucalyptus	0.366	1.48	1.22 ± 0.06	3.47 ± 0.36	1595 ± 1	62 ± 2	1339 ± 1	174 ± 4
Kraft pine/spruce	0.400	2.23	1.25 ± 0.05	3.43 ± 0.33	1594 ± 1	62 ± 2	1339 ± 1	169 ± 5
IS eucalyptus	0.396	1.88	1.43 ± 0.06	4.58 ± 0.43	1599 ± 1	57 ± 3	1337 ± 1	185 ± 7
IS spruce	0.408	3.17	1.46 ± 0.10	4.48 ± 0.65	1596 ± 2	62 ± 6	1339 ± 2	194 ± 7
IS miscanthus	0.391	1.60	1.43 ± 0.07	4.64 ± 0.46	1594 ± 1	61 ± 2	1339 ± 1	197 ± 7

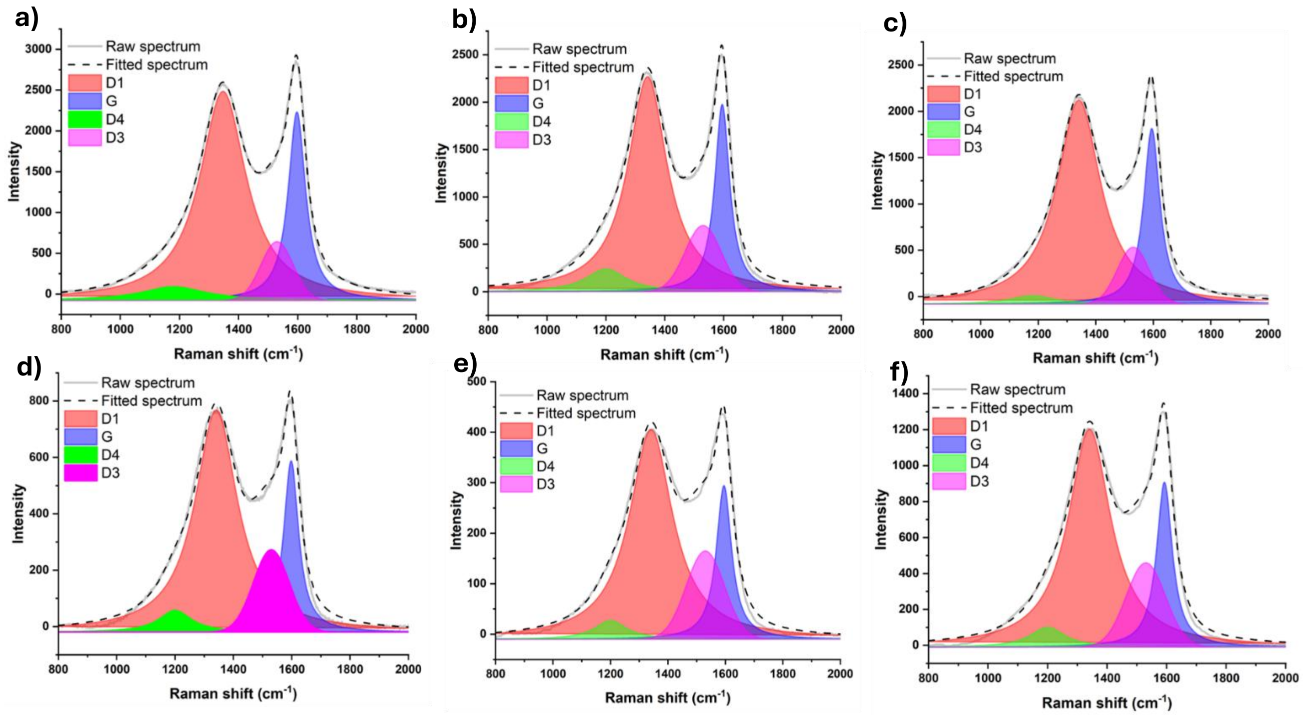

Figure S4: Example of deconvoluted Raman spectrum for a) organosolv beech, b) Kraft eucalyptus, c) Kraft pine/spruce, d) ionosolv eucalyptus, e) ionosolv spruce and f) ionosolv miscanthus-derived carbon nanofibres after carbonisation at 1000 °C.

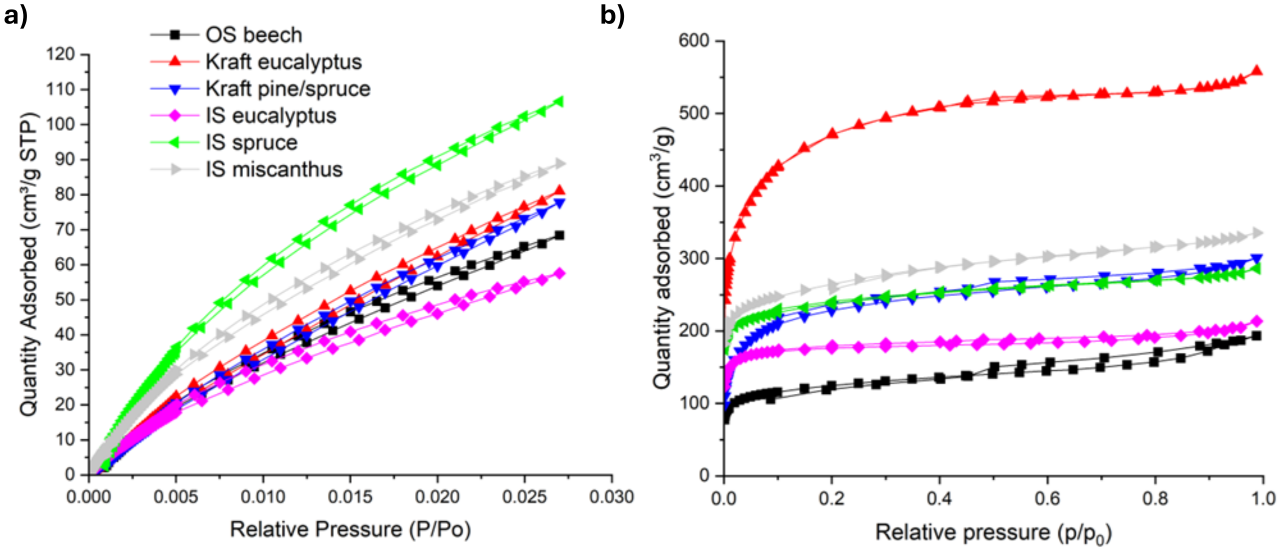

Figure S5: a) CO_2 and b) N_2 sorption isotherms of different LCNFs.

Table S7: Packing and skeletal density values of LCNFs.

**Lignin type**	**Packing density (g/cm³)**	**Skeletal density (g/cm³)**	**Volume packing fraction (packing/skeletal density)**
OS beech	0.135 ± 0.004	1.25 ± 0.07	0.108 ± 0.009
Kraft eucalyptus	0.054 ± 0.002	0.76 ± 0.02	0.071 ± 0.004
Kraft pine/spruce	0.077 ± 0.003	0.91 ± 0.03	0 085 ± 0.006
IS eucalyptus	0.087 ± 0.003	0.61 ± 0.03	0 143 ± 0.012
IS spruce	0.064 ± 0.002	1.06 ± 0.04	0.061 ± 0.004
IS miscanthus	0.065 ± 0.002	0.98 ± 0.04	0.066 ± 0.005

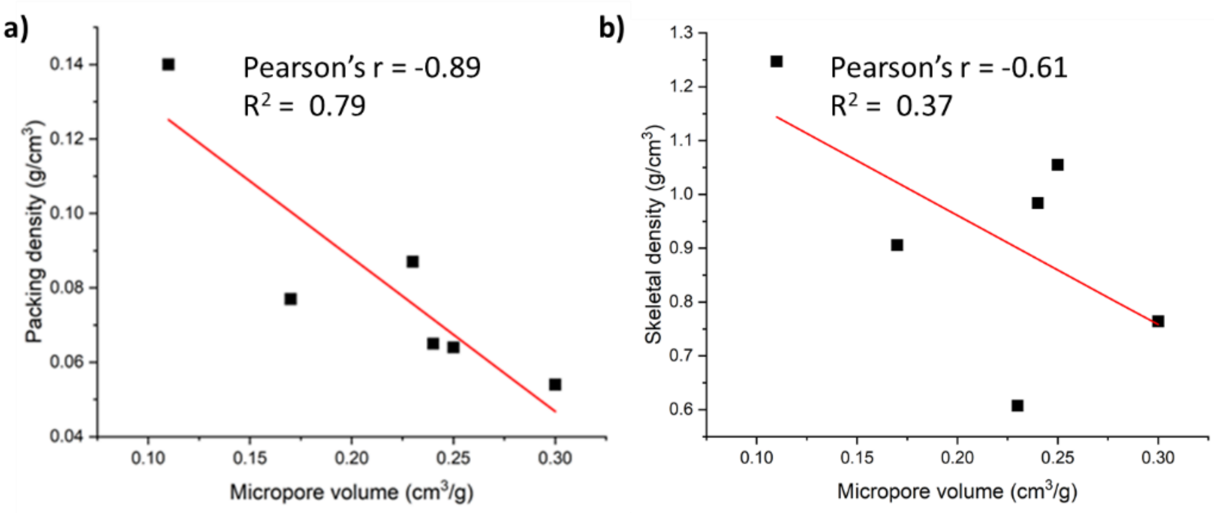

Figure S6: Plot of micropore volume, measured by N_2 sorption, and packing and skeletal density of LCNFs after carbonization at 1000 °C.

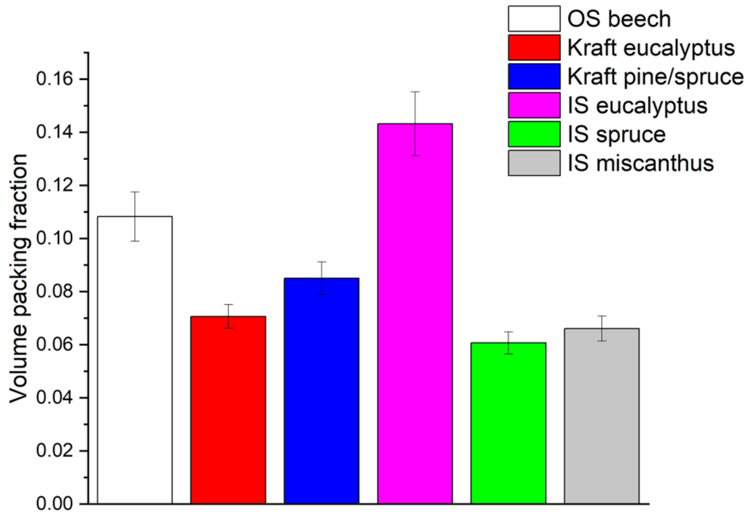

Figure S7: Volume packing fractions (packing density/skeletal density) of LCNFs after carbonization at 1000 °C

Table S8: Electronic conductivity values calculated from 4-point probe measurements. All measurements were collected in 5 different locations, and the average values are reported with uncertainly values.

**Lignin type**	**Electronic conductivity (S/cm)**	**Packing density normalized electronic conductivity (Scm^2/g)**
OS beech	15.98 ± 0.46	118.4 ± 6.9
Kraft eucalyptus	18.57 ± 4.46	343.9 ± 95.3
Kraft pine/spruce	10.27 ± 3.22	133.4 ± 47.0
IS eucalyptus	18.02 ± 5.14	207.1 ± 66.2
IS spruce	8.49 ± 2.55	132.7 ± 44.0
IS miscanthus	7.62 ± 1.91	117.2 ± 33.0

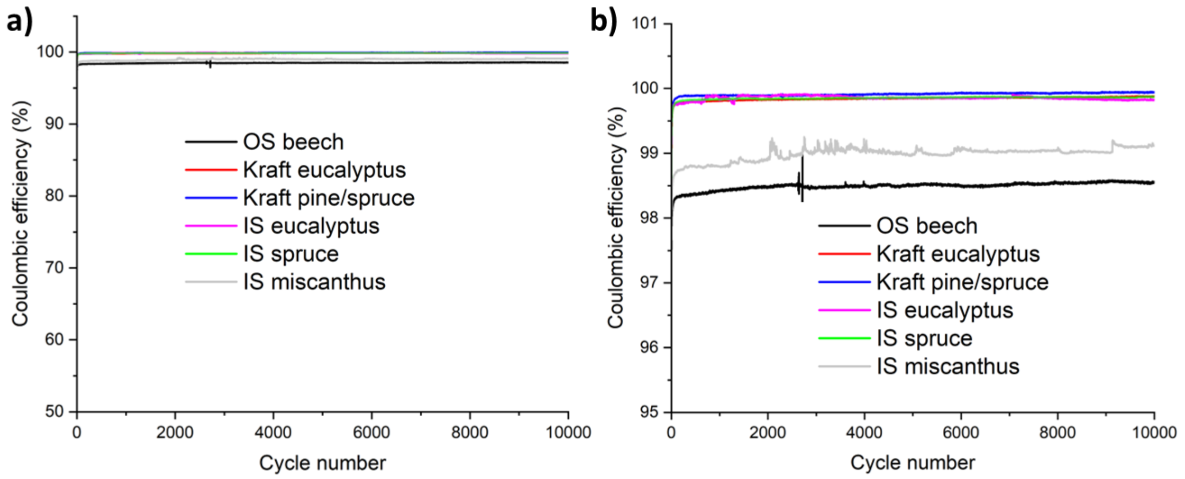

Figure S8: Coulombic efficiencies over 10,000 GCD cycles with different scales, (a) zoomed out, and (b) zoomed in.

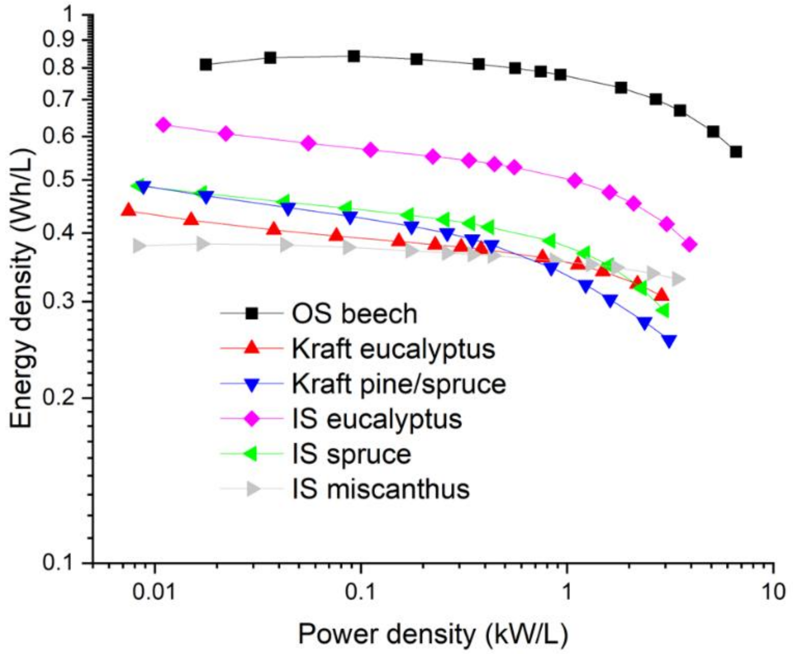

Figure S9: Volumetric Ragone plot of LCNFs.

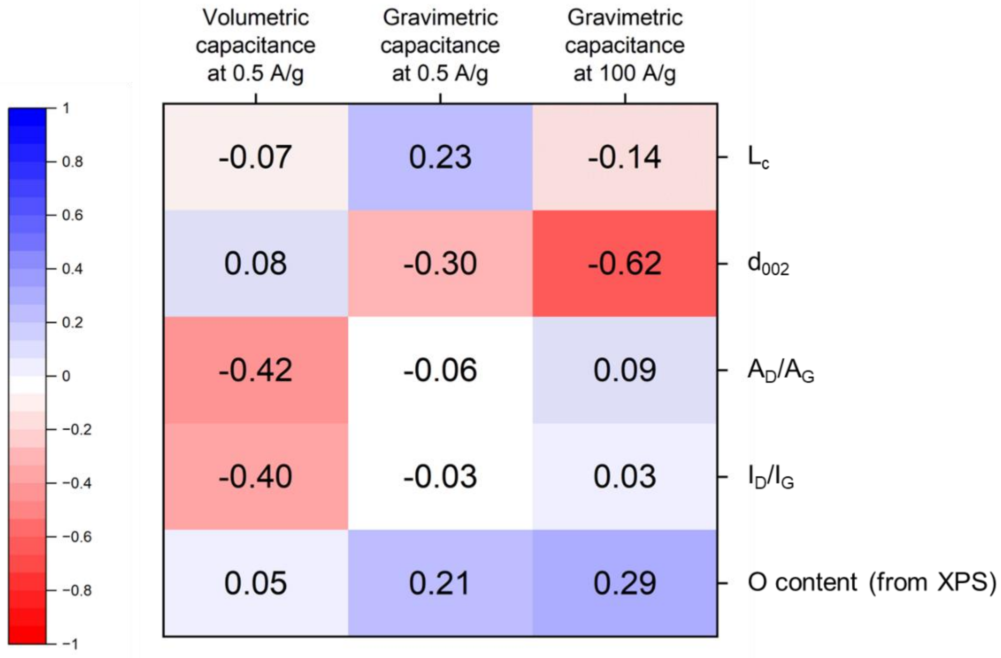

Figure S10: Heatmap indicating Pearson correlations between carbon structure characteristics of LCNF electrodes generated from different lignins and the volumetric and gravimetric capacitances at a current density of 0.5 A/g, and the gravimetric capacitance at 100 A/g. A Pearson coefficient ≥ 0.7 indicates a strong positive linear correlation, and a coefficient ≤ -0.7 indicates a strongly negative linear correlation.

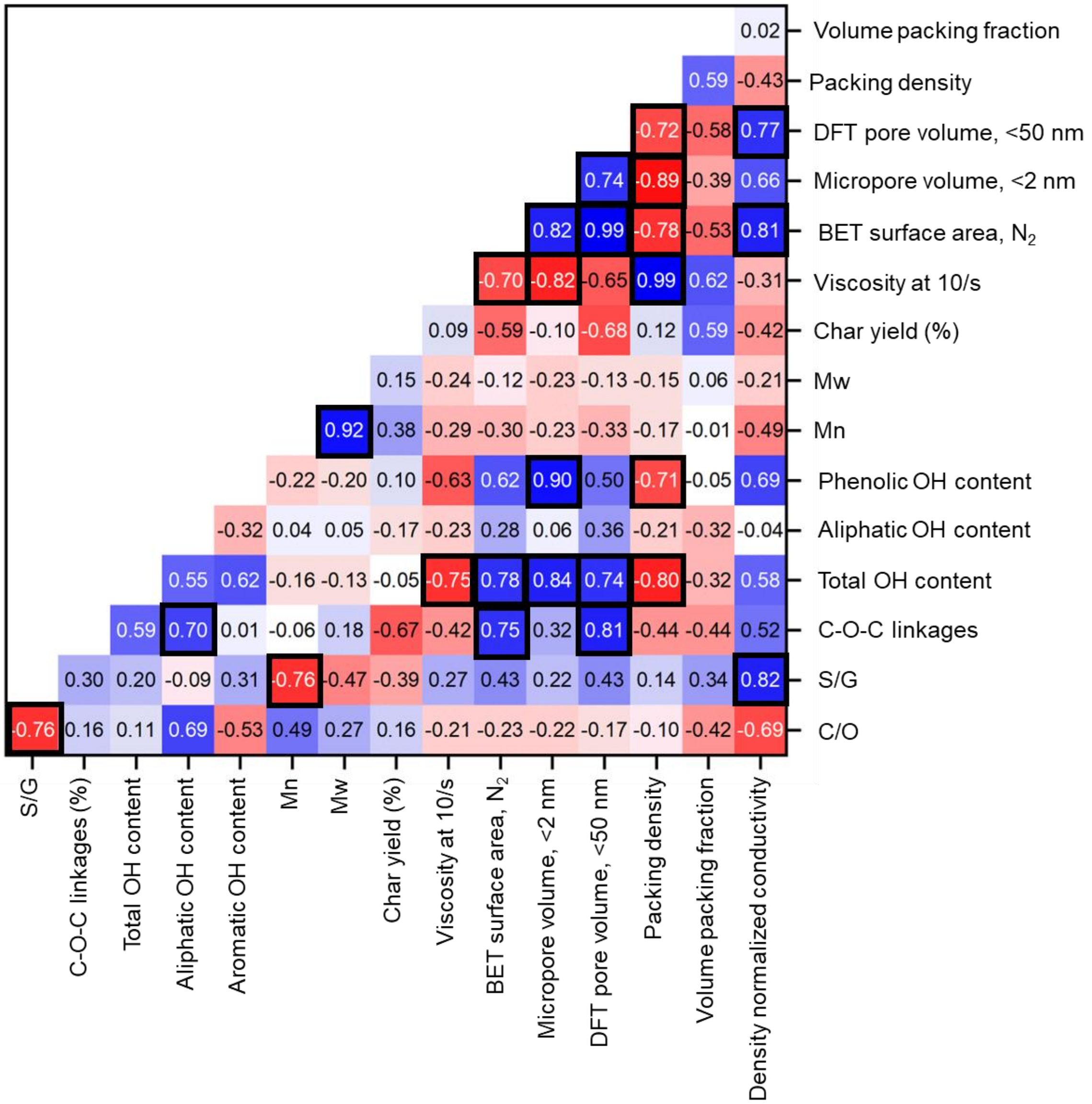

Figure S11: Heatmap indicating Pearson correlations between structural characteristics of the lignin precursor and properties of the resulting LCNFs. A Pearson correlation coefficient ≥ 0.7 indicates a strong positive correlation, and a coefficient ≤ -0.7 indicates a strongly negative correlation. Strong correlations are outlined in black.

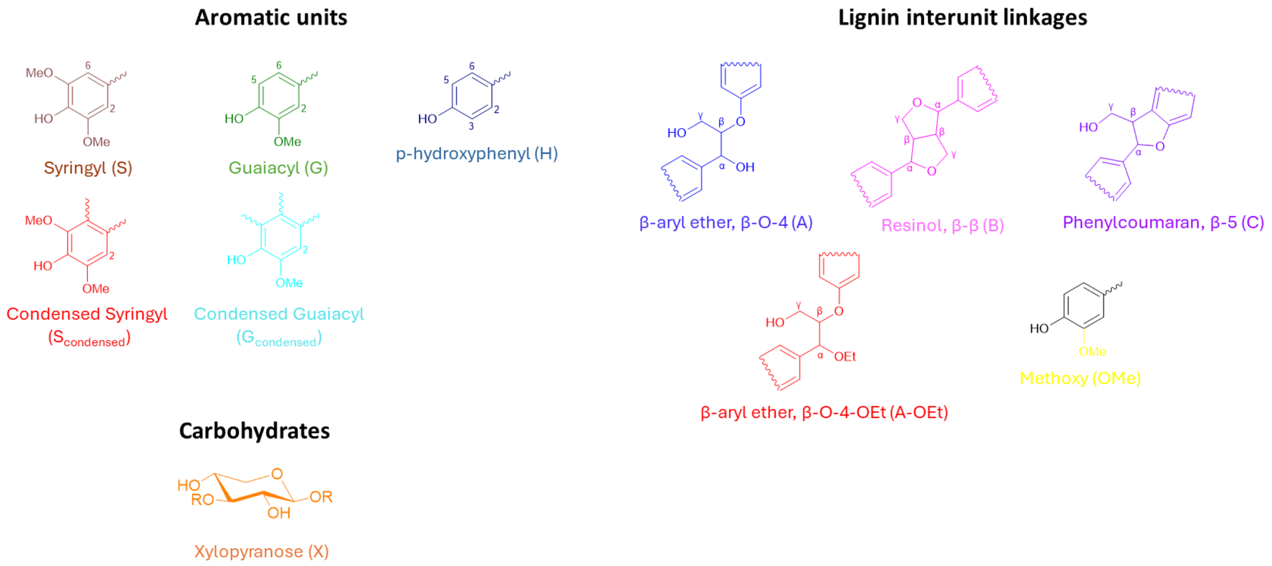

Figure S12: Structural motifs identified in the HSQC NMR spectra of the lignins.

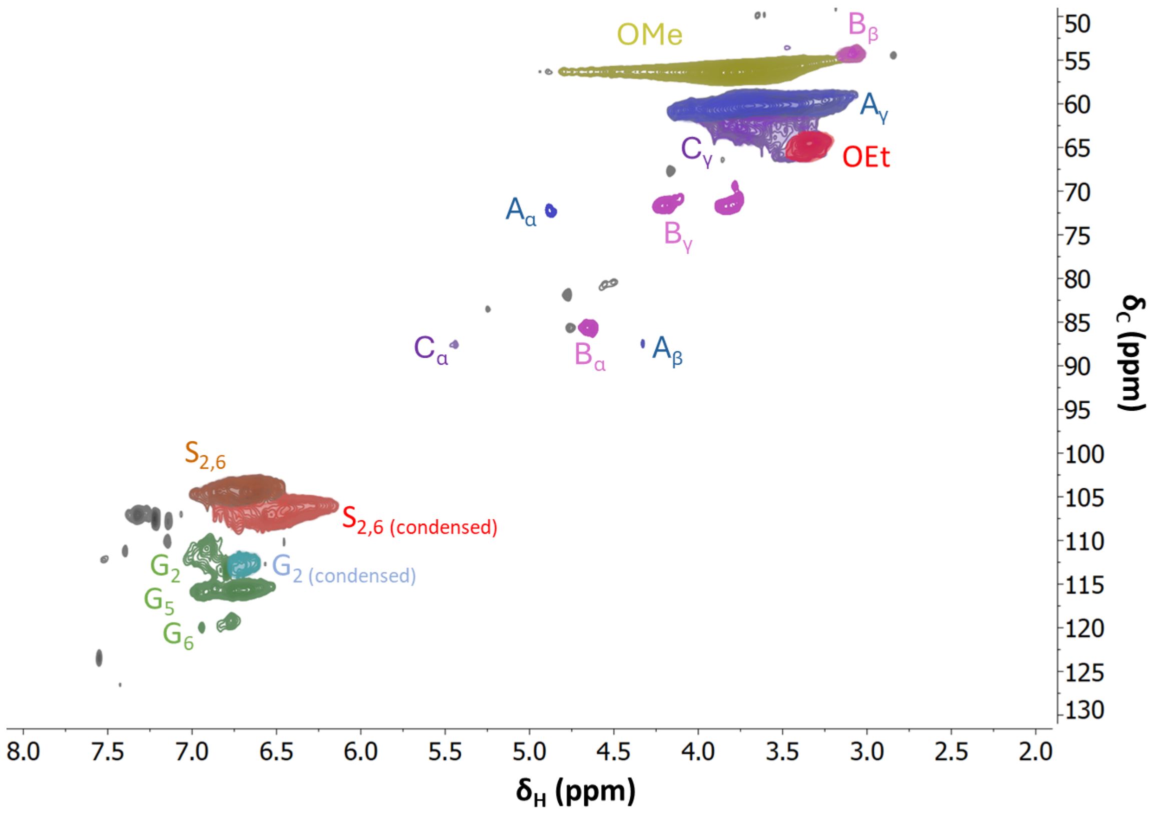

Figure S13: ^{1}H-^{13}C HSQC NMR spectrum of organosolv beech lignin.

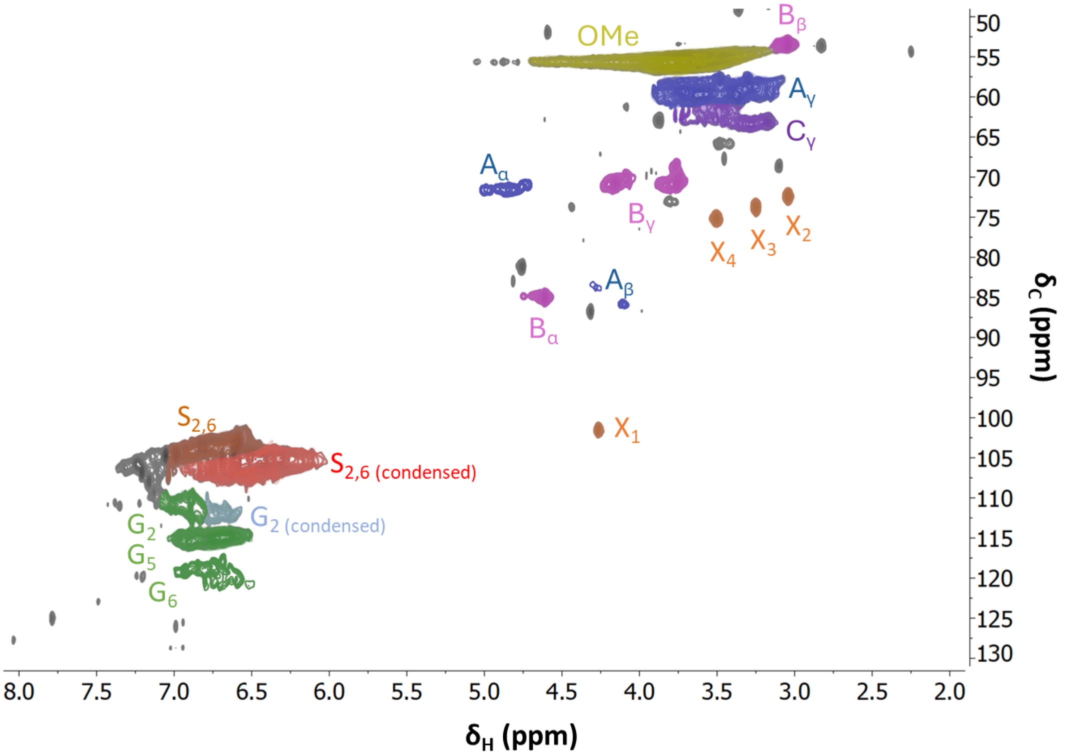

Figure S14: ^{1}H-^{13}C HSQC NMR spectrum of Kraft eucalyptus lignin.

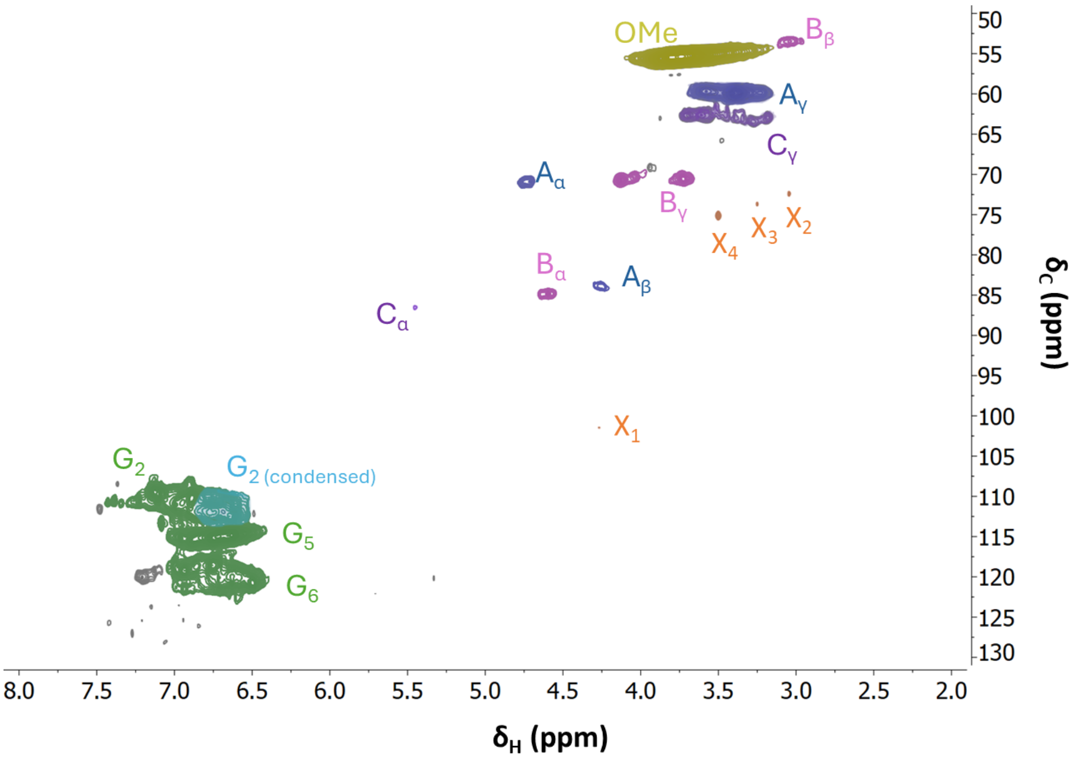

Figure S15: ^{1}H-^{13}C HSQC NMR spectrum of Kraft pine/spruce lignin.

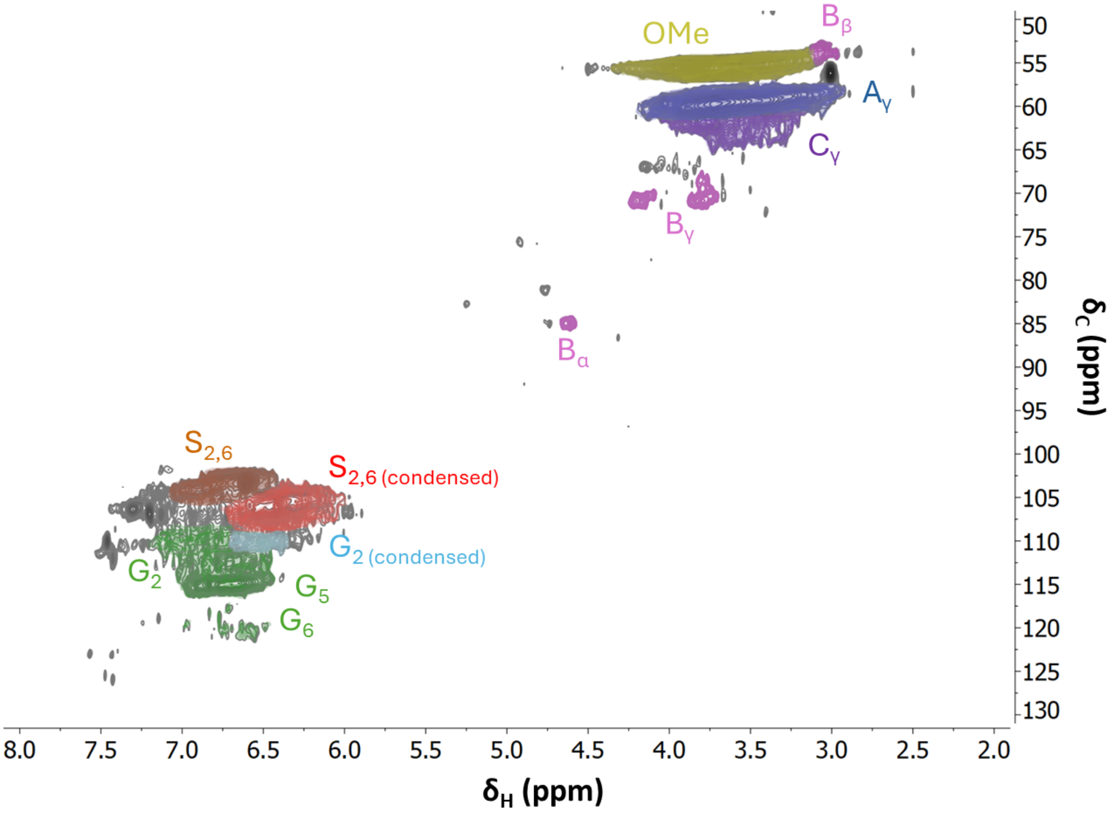

Figure S16: ^{1}H-^{13}C HSQC NMR spectrum of ionosolv eucalyptus lignin.

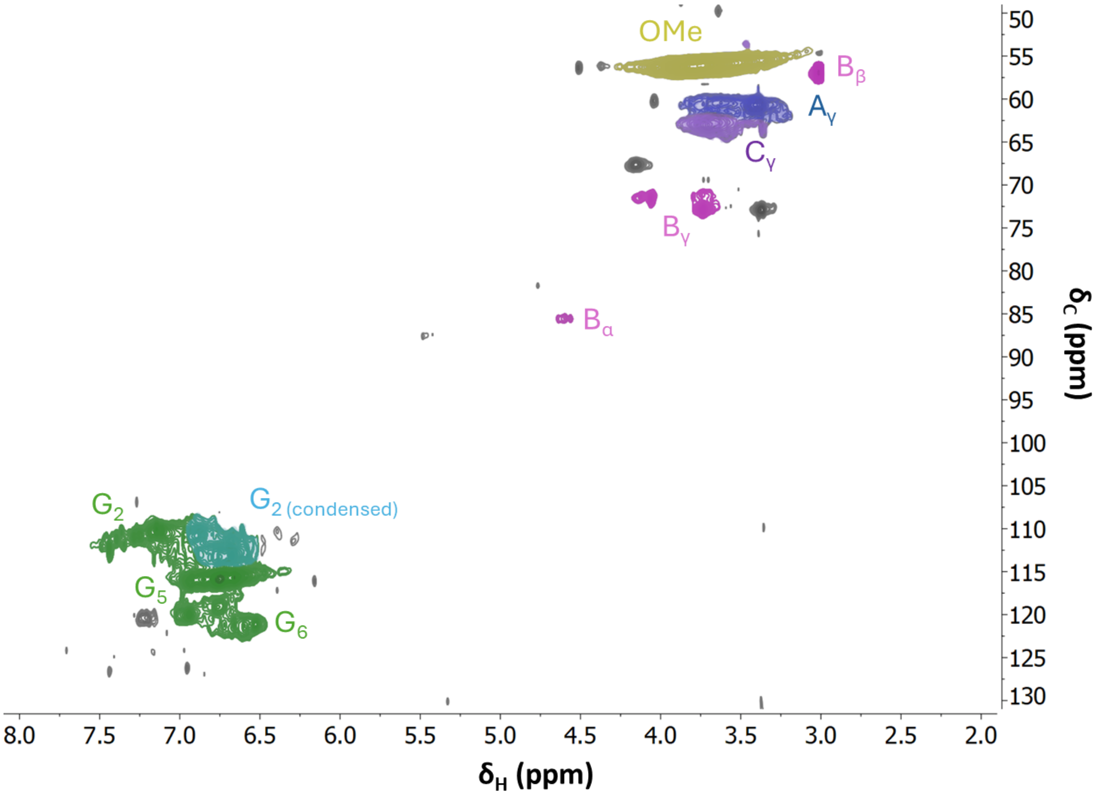

Figure S17: ^{1}H-^{13}C HSQC NMR spectrum of ionosolv spruce lignin.

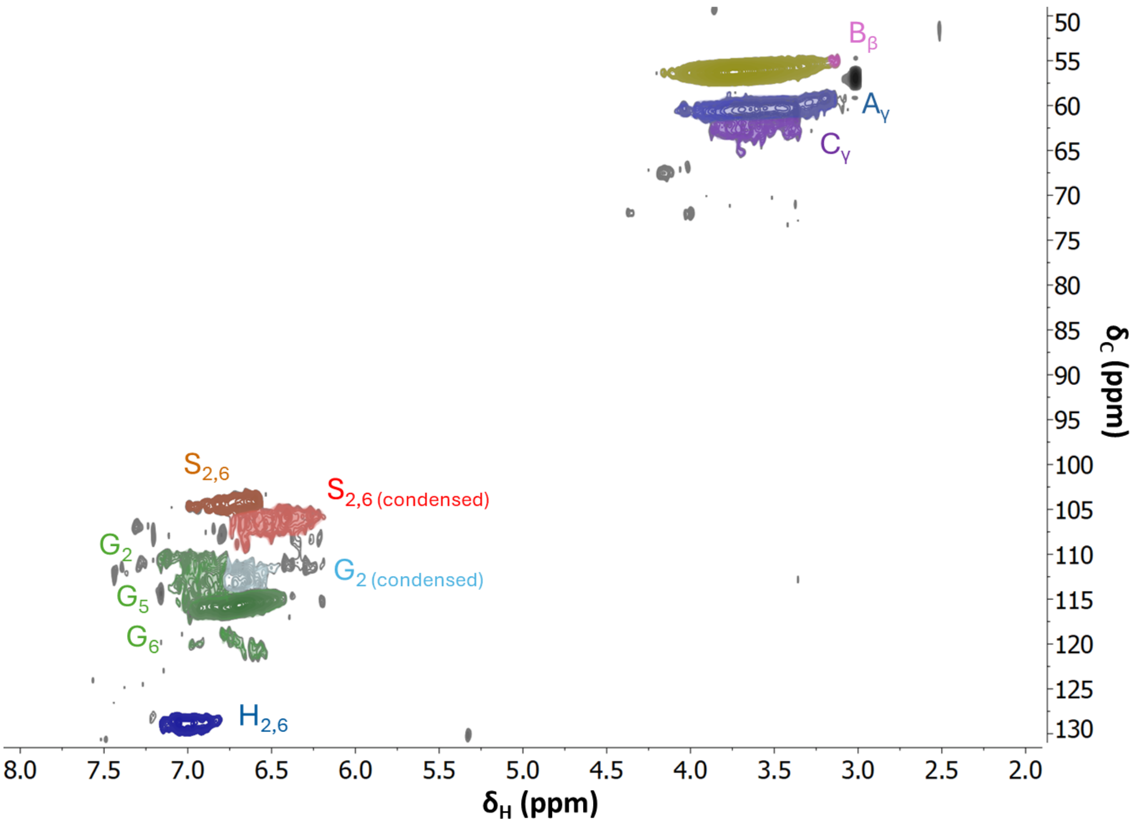

Figure S18: ^{1}H-^{13}C HSQC NMR spectrum of ionosolv miscanthus lignin.

Table S9: Estimated S-to-G subunit ratio and carbohydrate content (specifically xylose) of lignins calculated from integration of signals in HSQC NMR spectra.

**Lignin type**	**S/G**	**Xylose content (per 100 aromatic units)**
OS beech	1.65	0.31
Kraft eucalyptus	2.80	2.65
Kraft pine/spruce	0	1.13
IS eucalyptus	1.57	0.24
IS spruce	0	0.22
IS miscanthus	0.15	0.46

Table S10: Relative abundance of linkages based on integration of HSQC NMR spectra.

**Lignin type**	**C-O-C linkages (%)**	**C-C linkages (%)**
OS beech	34.8	65.2
Kraft eucalyptus	55.9	44.1
Kraft pine/spruce	47.6	52.4
IS eucalyptus	30.1	69.9
IS spruce	28.8	71.2
IS miscanthus	45.2	54.8

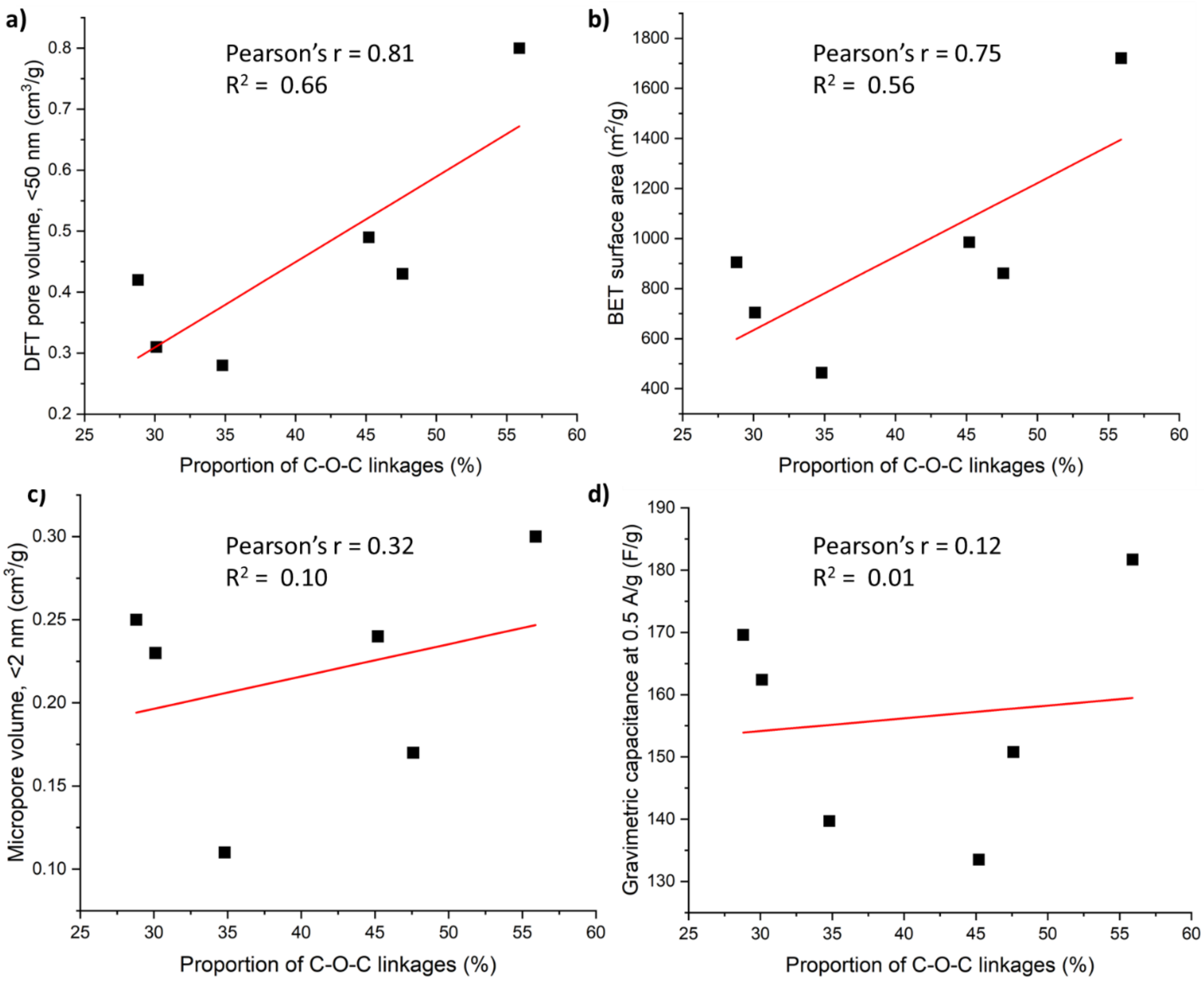

Figure S19: Plots of the proportion of beta-O-4 (C-O-C) linkages in lignin structure (from HSQC NMR spectroscopy) versus a) DFT pore volume, <50 nm, b) BET surface area, c) micropore volume, <2 nm and gravimetric capacitance at 0.5 A/g of the resulting LCNF electrodes.

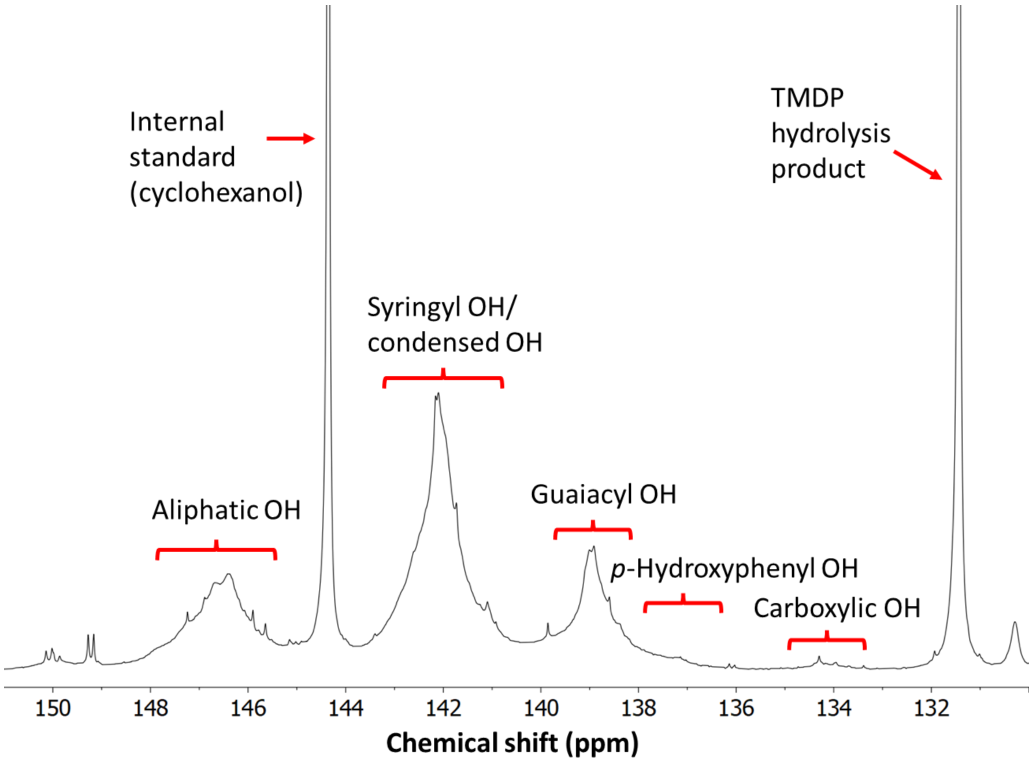

Figure S20: ^{31}P NMR spectrum of organosolv beech lignin.

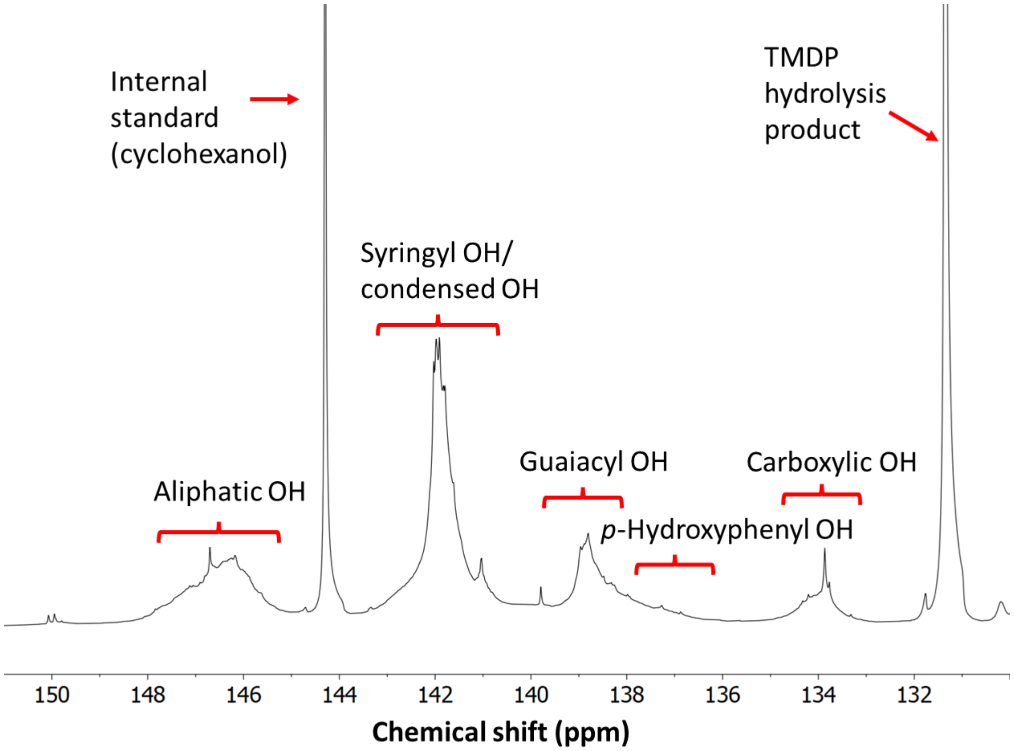

Figure S21: ^{31}P NMR spectrum of Kraft eucalyptus lignin.

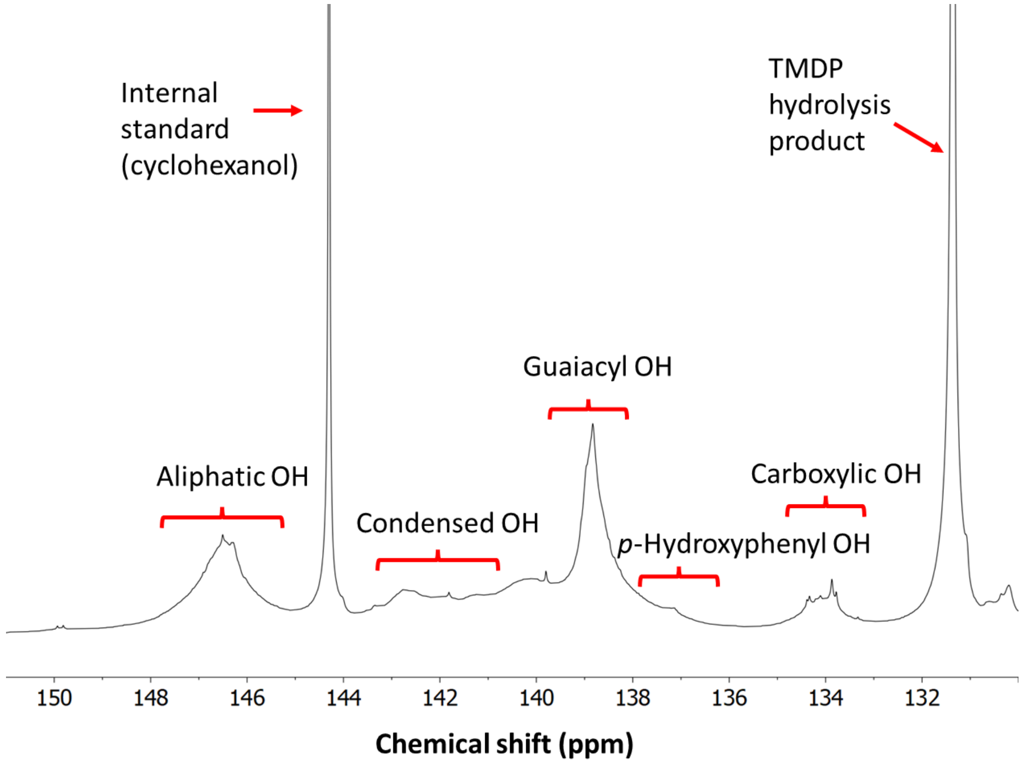

Figure S22: ^{31}P NMR spectrum of Kraft pine/spruce lignin.

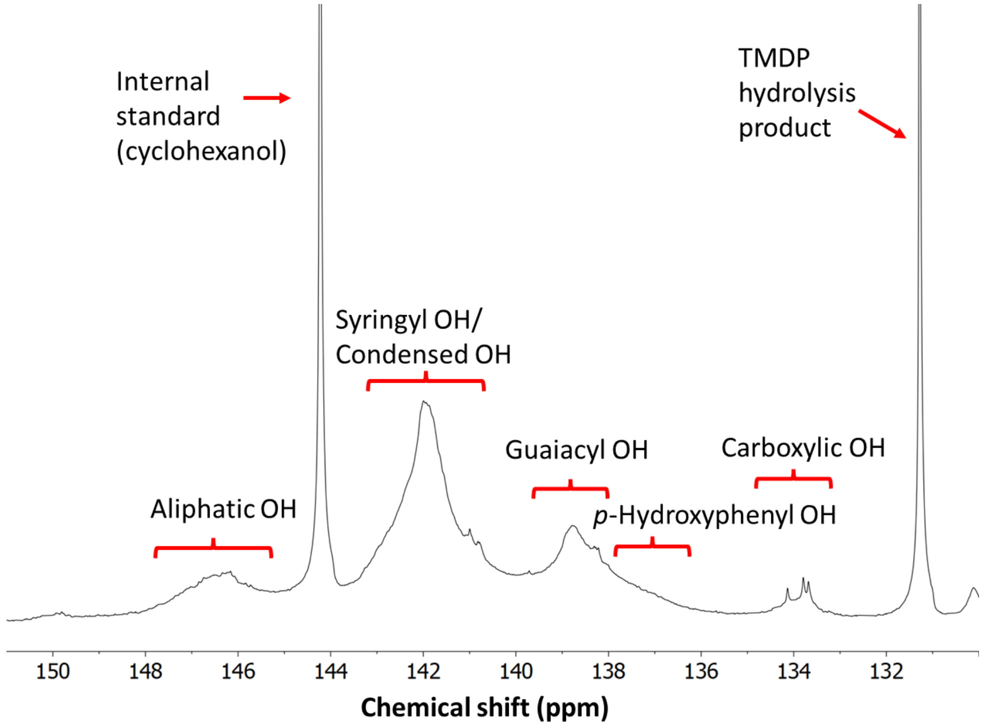

Figure S23: ^{31}P NMR spectrum of ionosolv eucalyptus lignin.

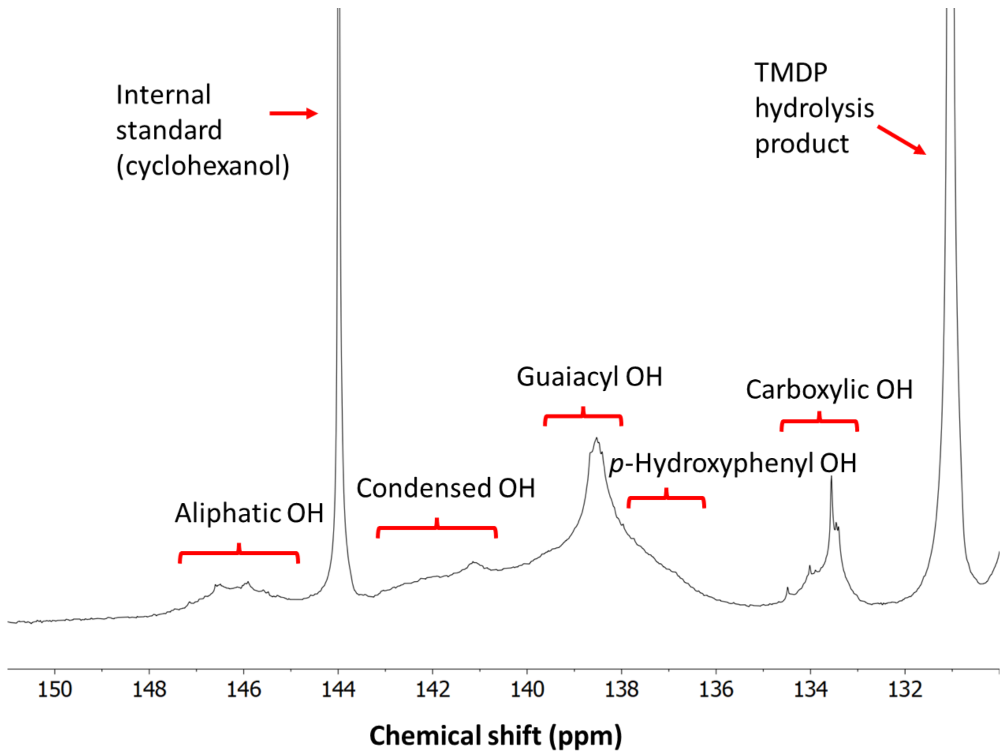

Figure S24: ^{31}P NMR spectrum of ionosolv spruce lignin.

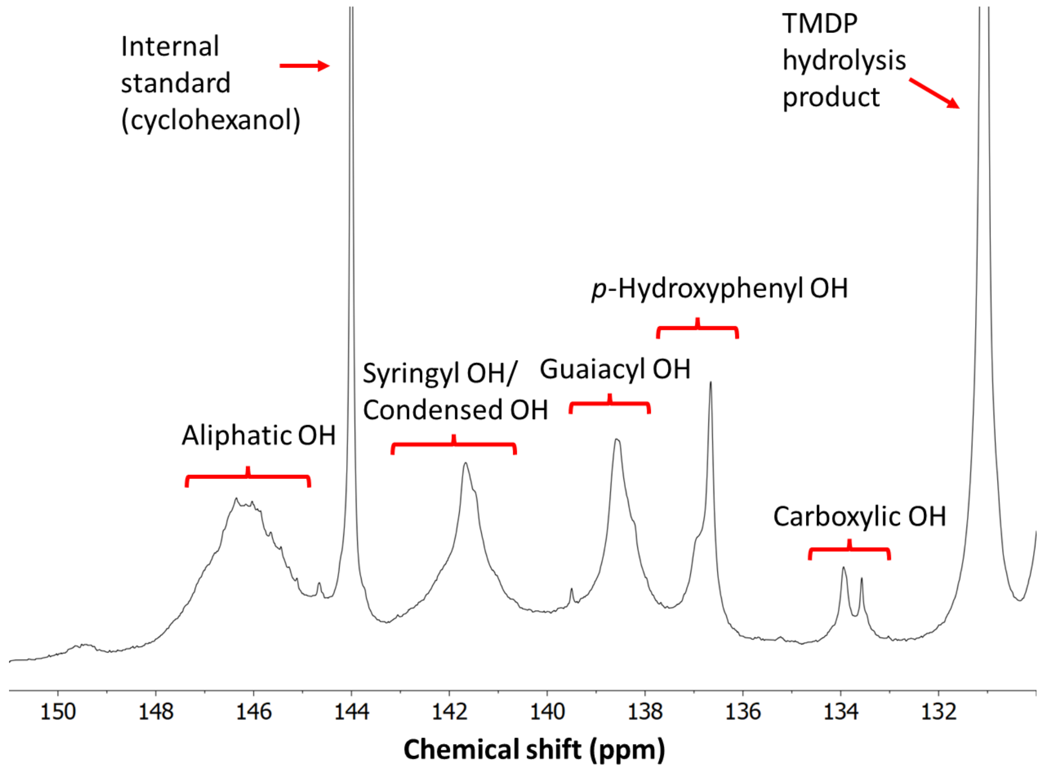

Figure S25: ^{31}P NMR spectrum of ionosolv miscanthus lignin.

Table S11: Hydroxyl group contents in different lignins determined by quantitative ^{31}P NMR spectroscopy.

**Lignin type**	**Total OH content (mmol/g)**	**Aliphatic OH content (mmol/g)**	**Phenolic OH content (mmol/g)**	**Aliphatic/phenolic OH**
OS beech	4.16	1.03	3.08	0.33
Kraft eucalyptus	6.00	1.38	4.45	0.31
Kraft pine/spruce	4.29	1.30	2.88	0.45
IS eucalyptus	5.09	0.82	4.00	0.21
IS spruce	4.88	0.35	3.77	0.10
IS miscanthus	6.70	2.11	4.08	0.52

Table S12: Distribution of hydroxyl functional groups in lignins determined by quantitative ^{31}P NMR spectroscopy.

**Lignin type**	**Aliphatic OH (mmol/g)**	**Syringyl OH /condensed phenolic OH (mmol/g)**	**Guaiacyl OH (mmol/g)**	**p-Hydroxyphenyl OH (mmol/g)**	**Carboxylic OH (mmol/g)**
OS beech	1.03	2.14	0.85	0.09	0.06
Kraft eucalyptus	1.38	3.35	1.10	0.00	0.17
Kraft pine/spruce	1.30	0.72	2.15	0.01	0.11
IS eucalyptus	0.82	2.52	1.20	0.28	0.27
IS spruce	0.35	0.86	2.32	0.58	0.77
IS miscanthus	2.11	1.58	1.56	0.94	0.51

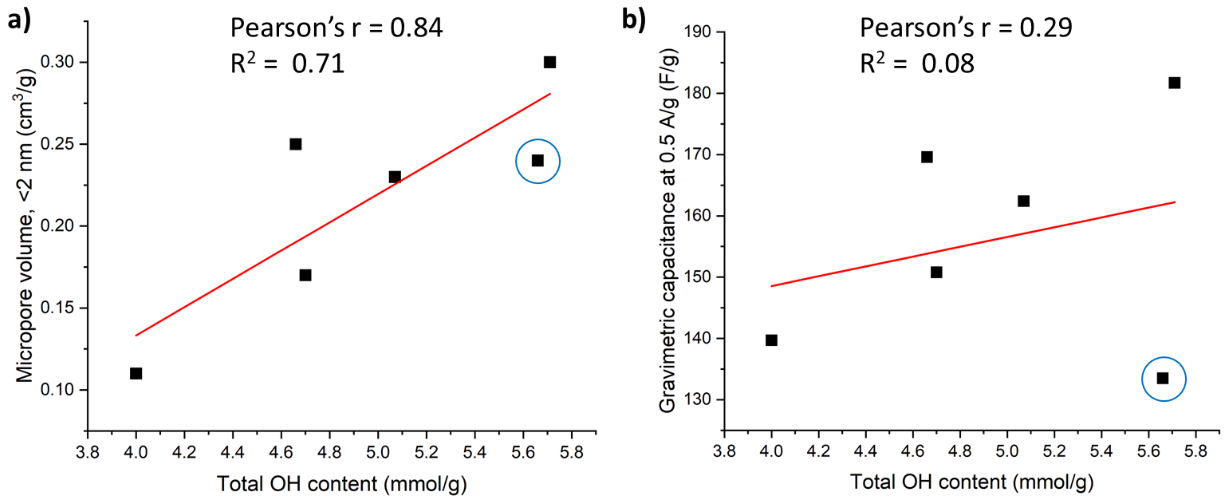

Figure S26: Plots of total hydroxyl group content in lignins, measured by quantitative ^{31}P NMR spectroscopy versus a) micropore volume and b) gravimetric capacitance at 0.5 A/g of resulting LCNF electrodes with linear fit. Data points corresponding to ionosolv miscanthus LCNF circled.

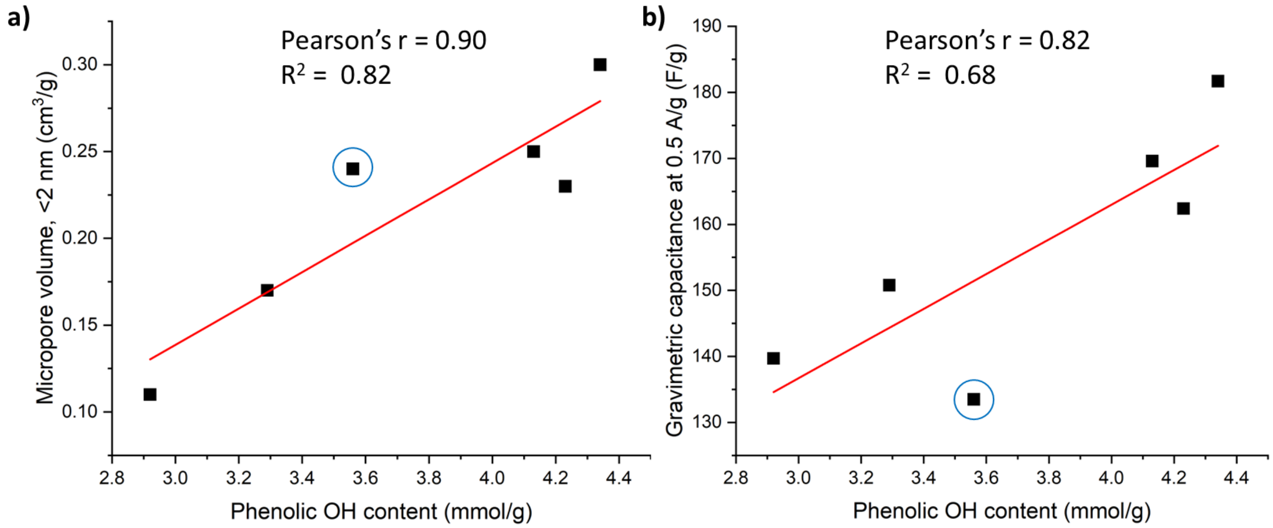

Figure S27: Plots of phenolic hydroxyl group content in lignins, measured by quantitative ^{31}P NMR spectroscopy versus a) micropore volume and b) gravimetric capacitance at 0.5 A/g of resulting LCNF electrodes with linear fit. Data points corresponding to ionosolv miscanthus LCNF circled.

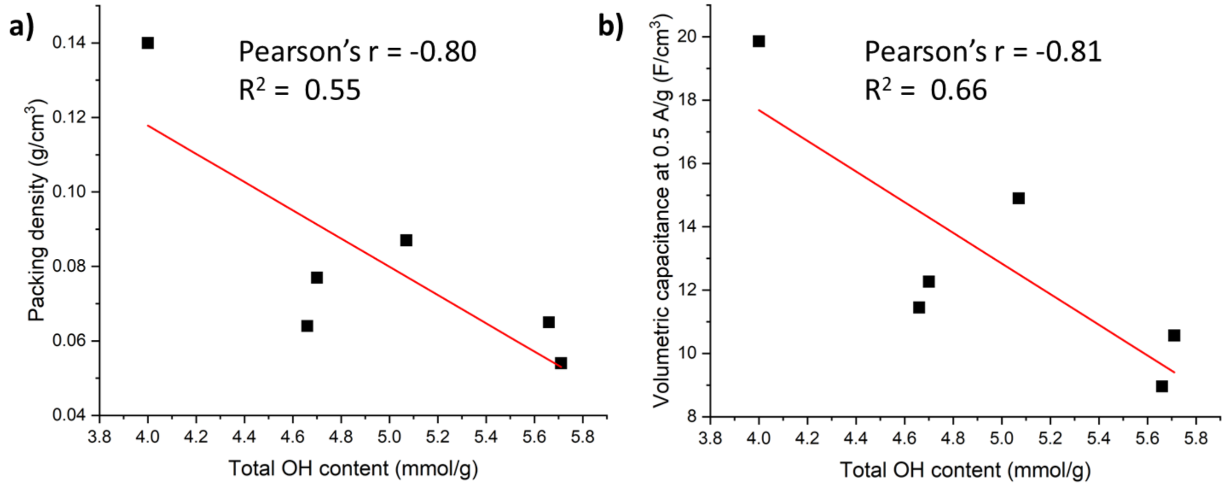

Figure S28: Plots of total hydroxyl group content in lignins versus a) packing density and b) volumetric capacitance at 0.5 A/g of resulting LCNF electrodes with linear fit.

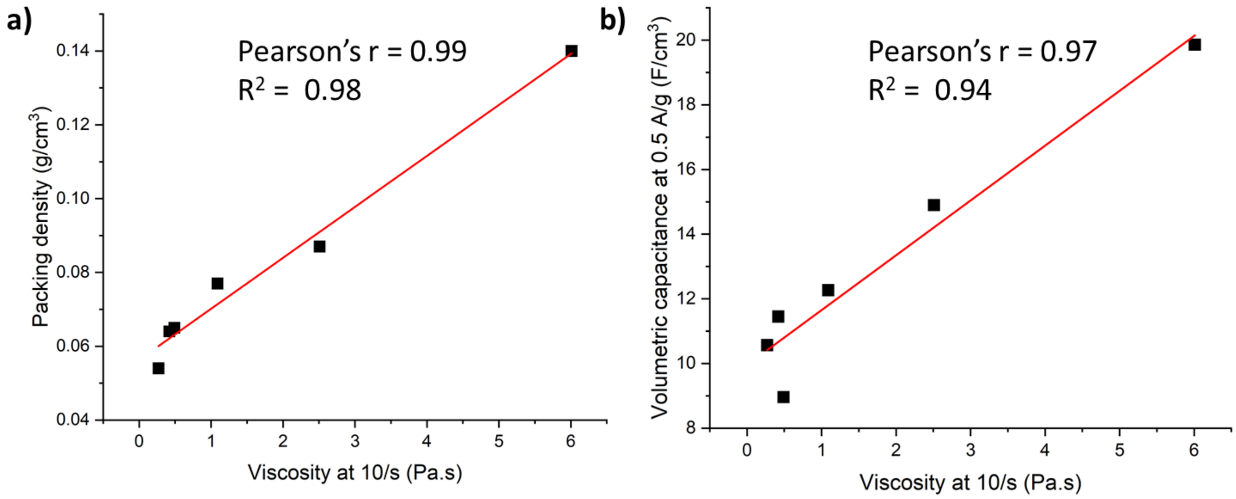

Figure S29: Plots of viscosity of lignin/PEO/NaOH spinning dope solutions at a shear rate of 10/s versus a) packing density and b) volumetric capacitance at 0.5 A/g of resulting LCNF electrodes with linear fit.

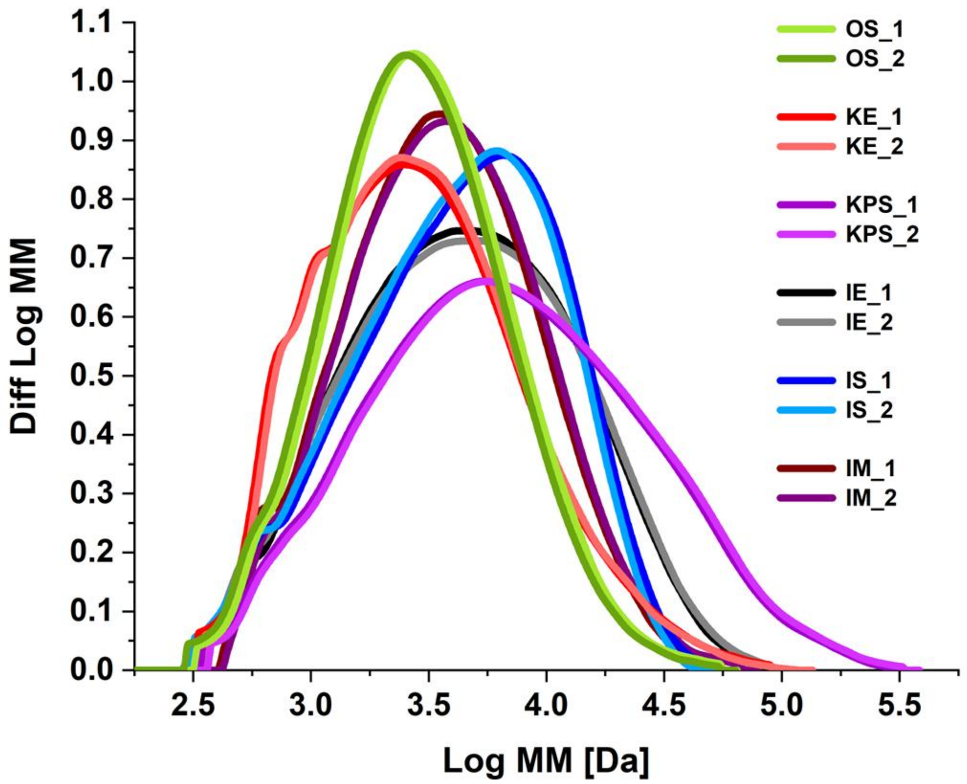

Figure S30: Molecular weight distributions for the extracted lignins (OS = Organosolv beech, KE = Kraft eucalyptus, KPS = Kraft pine/spruce, IE = Ionosolv eucalyptus, IS = Ionosolv spruce, IM = Ionosolv miscanthus).

Table S13: Number average molecular weight (M_n), weight average molecular weight (M_w) and dispersity (D) extracted from the GPC data.

**Lignin type**	**M_n (Da)**	**M_w (Da)**	**D**
OS beech	2023 ± 76	4275 ± 152	2.11 ± 0.01
Kraft eucalyptus	1891 ± 36	5134 ± 19	2.72 ± 0.04
Kraft pine/spruce	3263 ± 85	15717 ± 448	4.82 ± 0.02
IS eucalyptus	2484 ± 34	7787 ± 89	3.14 ± 0.08
IS spruce	2513 ± 71	6375 ± 213	2.54 ± 0.02
IS miscanthus	2436 ± 69	5499 ± 226	2.26 ± 0.03

Table S14: Elemental composition of lignins determined by CHNS analysis, ash content, and the resulting carbon-to-oxygen ratio (C/O).

**Lignin type**	**C%**	**H%**	**N%**	**S%**	**Ash content (%)**	**C/O**
OS beech	63.23 ± 0.02	5.58 ± 0.02	0.215 ± <0.01	<0.01	1.30 ± 0.20	2.04
Kraft eucalyptus	59.59 ± 0.02	5.75 ± 0.02	0.225 ± <0.01	2.815 ± 0.015	1.14 ± 0.10	1.88
Kraft pine/spruce	63.66 ± 0.02	5.715 ± <0.01	0.14 ± <0.01	1.83 ± 0.09	1.23 ± 0.12	2.22
IS eucalyptus	61.195 ± 0.175	5.38 ± 0.15	0.455 ± 0.055	1.105 ± 0.175	2.85 ± 0.02	1.92
IS spruce	60.5 ± 0.05	5.805 ± 0.205	1.51 ± 0.02	2.7 ± 0.11	2.87 ± 0.22	2.05
IS miscanthus	63.235 ± 0.115	7.205 ± 0.175	0.78 ± 0.03	2.525 ± 0.175	1.59 ± 0.09	2.4

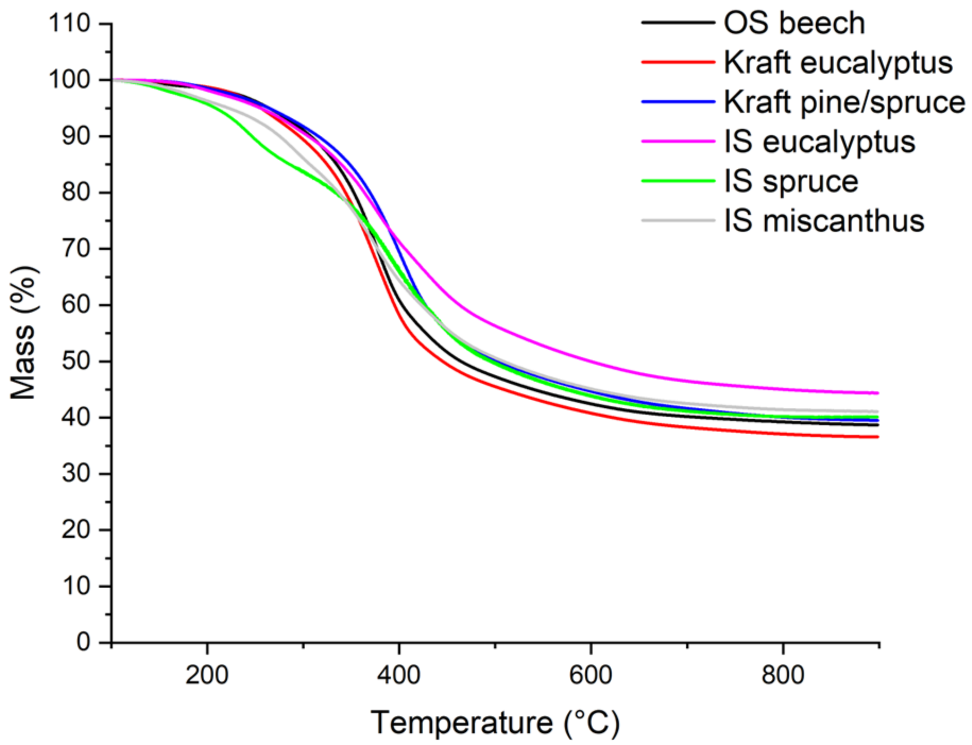

Figure S31: Char yield determined using TGA (under N_2) for different lignins, which can be used to estimate the carbon fibre mat yield.

Table S15: Carbonization yield for lignins at 900 °C, as determined by TGA (under N_2).

**Lignin type**	**Char yield (%)**
OS beech	38.8 ± 0.1
Kraft eucalyptus	35.9 ± 0.7
Kraft pine/spruce	37.6 ± 1.9
IS eucalyptus	43.8 ± 0.6
IS spruce	39.6 ± 0.6
IS miscanthus	41.1 ± 1.5

Table S116: Compositions of organosolv beech lignin spinning dope solutions with 15 and 12% solid loadings.

Lignin type	Solid loading (Lignin + PEO) (%)	Mass of lignin (g)	Mass of PEO (g)	M_w of PEO (kDa)	Mass of 0.5M NaOH (aq) solution (g)	Viscosity at 10/s (Pa.s)
Organosolv beech	15	1.2	0.15	400	7.8	6.01
Organosolv beech	12	1.2	0.15	400	10	0.16

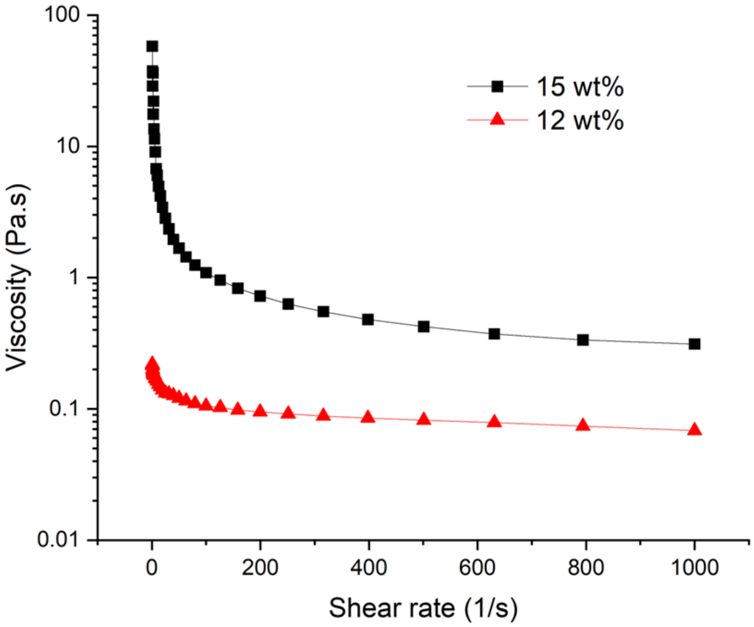

Figure S32: Viscosity measurements of organosolv beech lignin spinning dope solutions with 15 and 12% solid loadings.

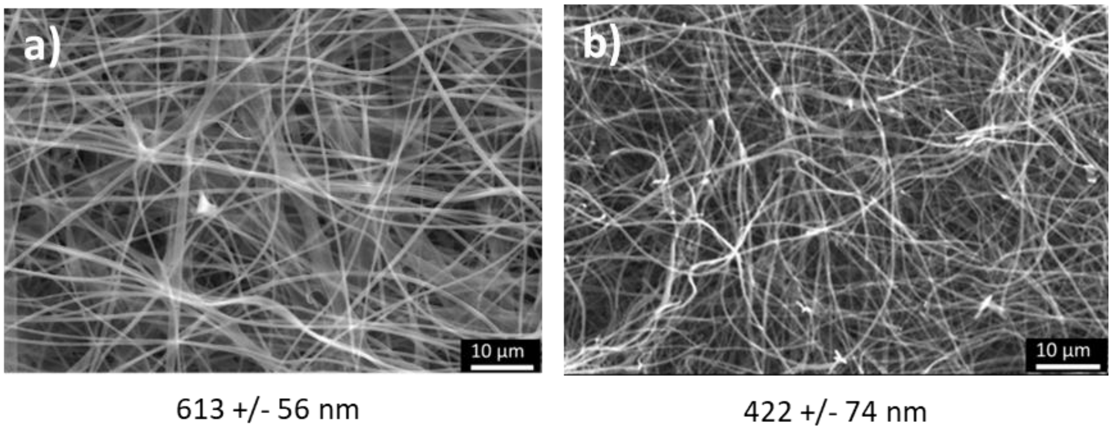

Figure S33: SEM images, with average nanofibre diameters, of organosolv beech lignin-derived CNFs electrospun from dope solutions containing a) 15% and b) 12% solid loadings after carbonization at 1000 °C.

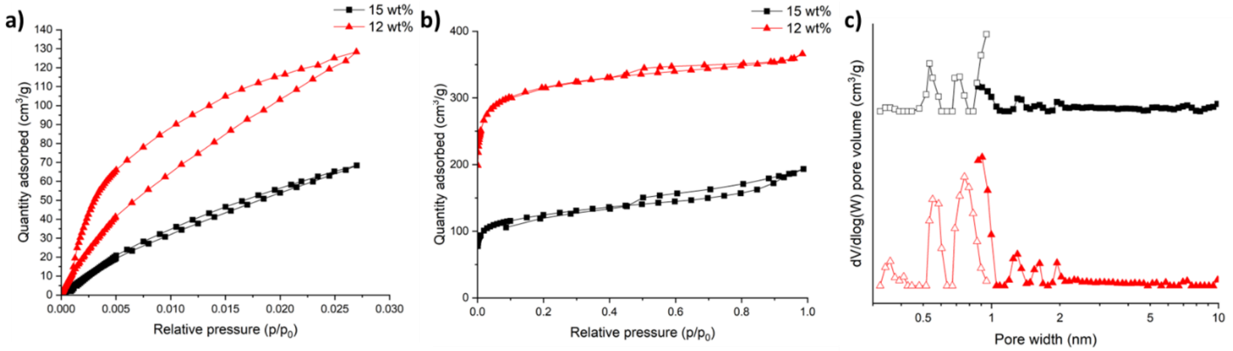

Figure S34: a) CO_2 and b) N_2 sorption isotherms of organosolv beech LCNFs prepared using 15 and 12% solid loadings and c) calculated pore size distributions (unfilled symbols are derived from CO_2 sorption and filled symbols from N_2 sorption measurements).

Table S1127: Porosity characteristics of organosolv beech LCNFs prepared using 15 and 12% solid loadings.

**Lignin type/solid content**	**Total pore volume, N_2 (cm^3/g)**	**BET surface area, N_2 (m^2/g)**	**Micropore volume, N_2, t-plot (cm^3/g)**	**DFT pore volume, N_2, t-plot (cm^3/g)**	**DFT surface area, N_2 (m^2/g)**	**DFT pore volume, CO_2 (cm^3/g)**	**DFT surface area, CO_2 (m^2/g)**
OS beech / 15%	0.30	464	0.11	0.28	207	0.21	605
OS beech / 12%	0.57	1212	0.36	0.53	638	0.45	1424

Table S18: Density characteristics of organosolv beech LCNFs prepared using 15 and 12% solid loadings.

Lignin type/solid content	Packing density (g/cm³)	Skeletal density (g/cm³)	Volume packing fraction
OS beech / 15%	0.135 ± 0.004	1.25 ± 0.07	0.108 ± 0.009
OS beech / 12%	0.102 ± 0.003	0.71 ± 0.01	0.144 ± 0.008

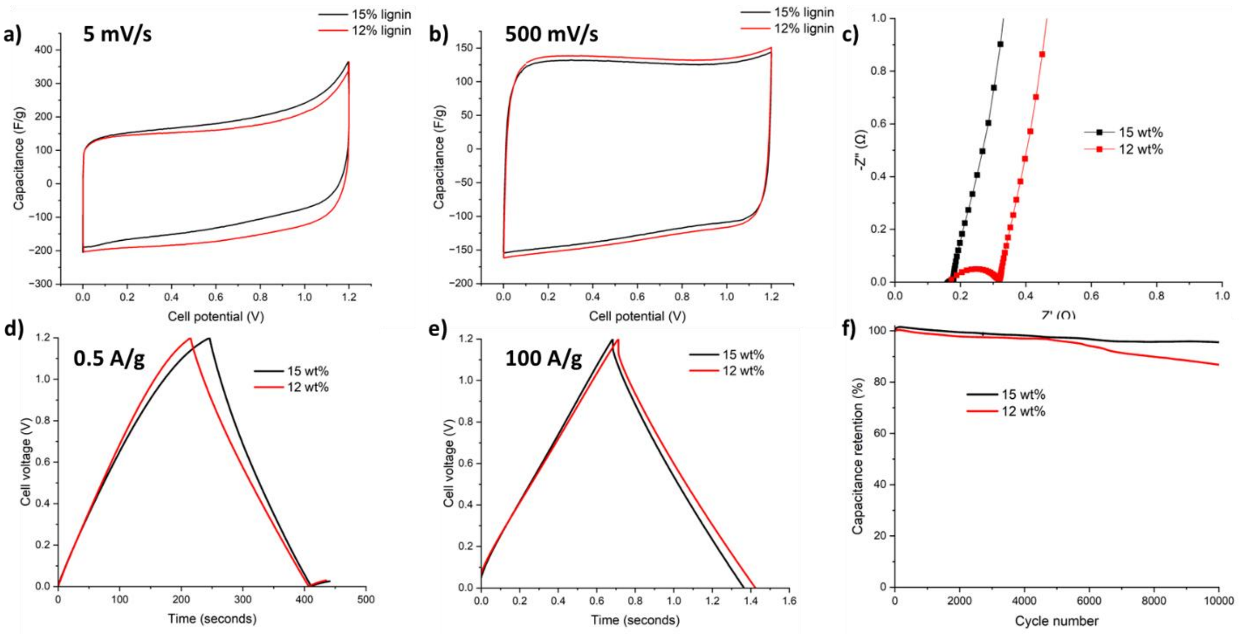

Figure S35: Electrochemical performance of organosolv beech LCNFs prepared using a 15 and 12% solid content in a two-electrode cell. CV curves at a) 5 mV/s and b) 500 mV/s, c) Nyquist plot from low frequency region of the EIS spectra, GCD curves at current densities of d) 0.5 A/g and e) 100 A/g, and f) capacitance retention over 10,000 GCD cycles at 10 A/g.

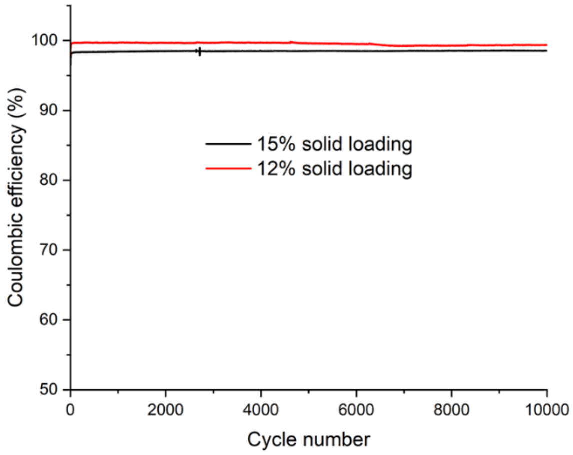

Figure S36: Coulombic efficiency over 10,000 GCD cycles at 10 A/g.

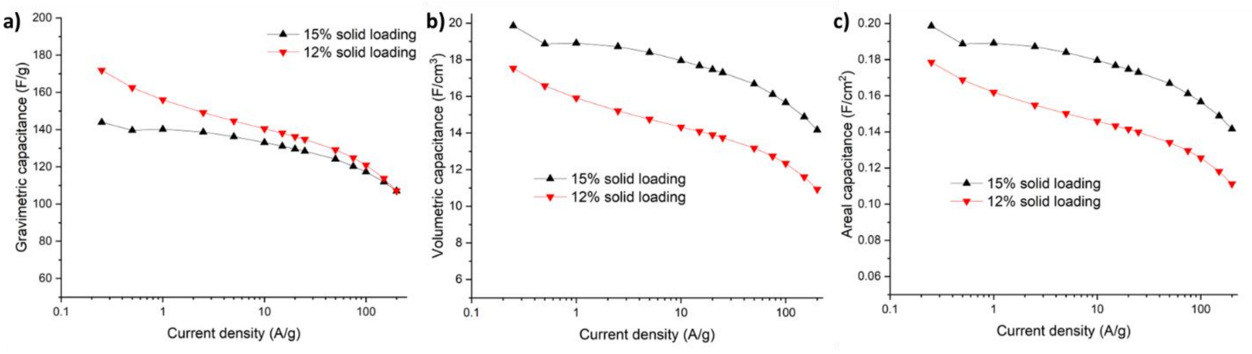

Figure S37: a) Gravimetric capacitance as a function of current density, b) volumetric capacitance and c) areal capacitance as a function of current density, calculated from GCD curves, for organosolv beech LCNFs prepared using 15 and 12% solid loadings.

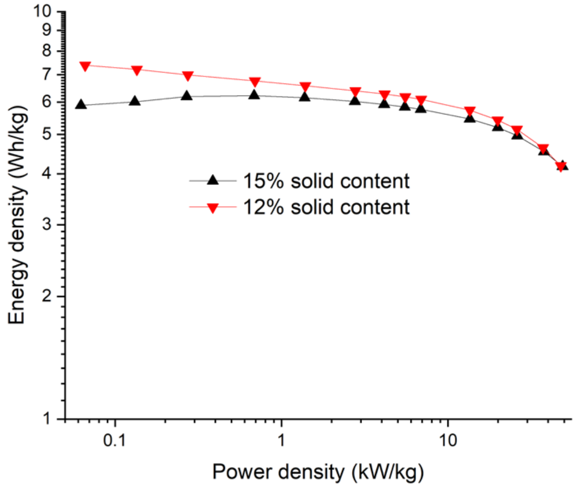

Figure S38: Ragone plot for organosolv beech LCNFs prepared using 15 and 12% solid loadings.

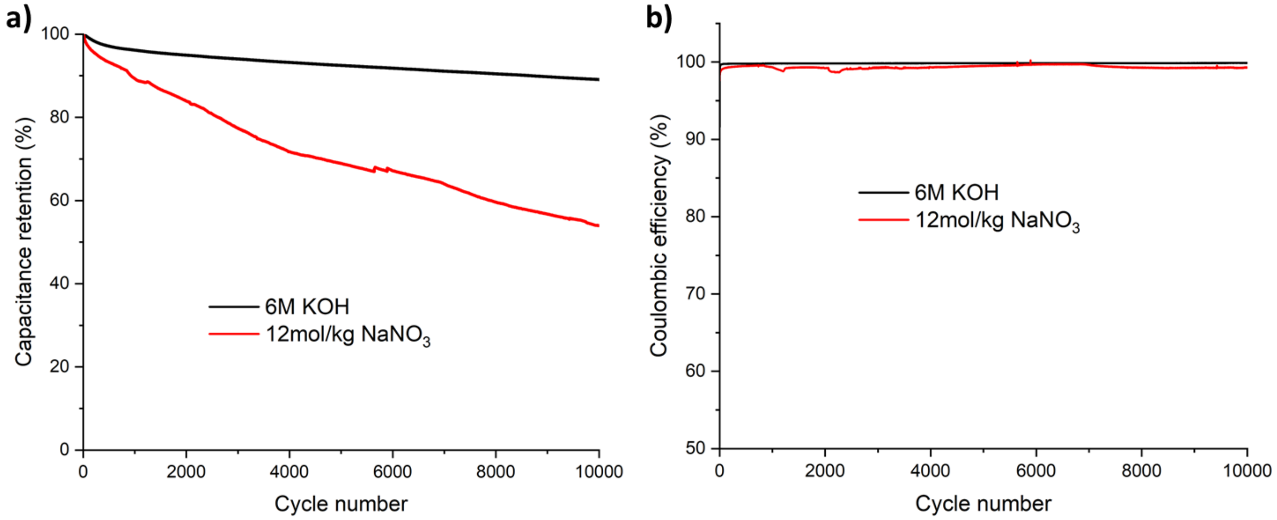

Figure S39: a) Capacitance retention and b) coulombic efficiency of Kraft eucalyptus-derived CNFs in symmetric cell with 6M KOH and 12 mol/kg over 10,000 charge-discharge cycles.

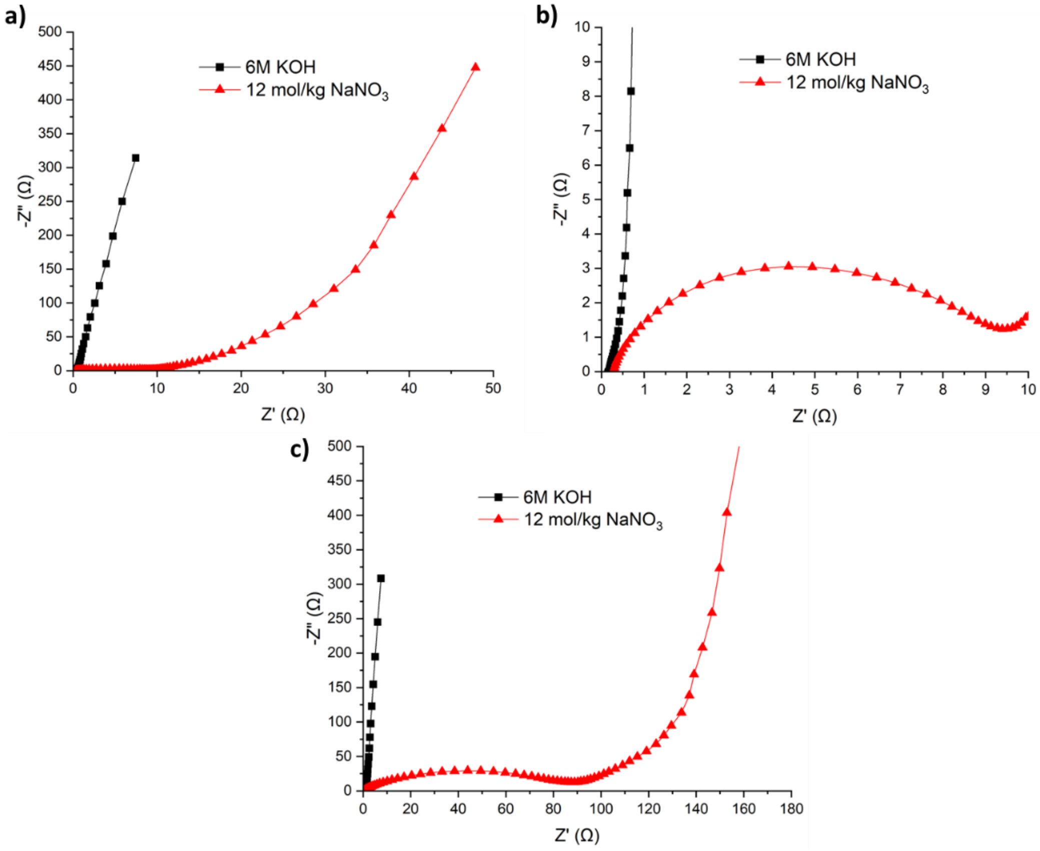

Figure S40: a) Nyquist plot and b) zoomed in Nyquist plot of Kraft eucalyptus-derived CNFs in symmetric cell with 6 M KOH and 12 $molkg^{-1}$ $NaNO_3$ as electrolyte. c) Nyquist plot of same cells after 10,000 GCD cycles.

References

1. C. Che, Y. Lv, X. Wu, P. Dong, N. Liang, H. Gao and J. Guo, *Diamond Relat. Mater.*, 2023, **137**, 110140.
2. L. Gao, Y. Wang, Y. Liu and L. Xu, *Colloids Surf., A,* 2023, **663**, 131056.
3. X. Gan, R. Yuan, J. Zhu, Q. Li, T. Tang, F. Qin, L. Zhu, J. Zhang, L. Wang, S. Zhang, H. Song and D. Jia, *Carbon*, 2023, **201**, 381-389.
4. C. Zhao, N. Sun, M. Li, W. Cai, W. Jiang and C. Zhao, *Energy Fuels*, 2023, **37**, 13467-13475.
5. B. Üstün, H. Aydın, S.N. Koç, A. Uluslu and Ü. Kurtan, *Journal of Energy Storage*, 2023, **73**, 108970.
6. S. Lv, X. Wu, Y. Lv, Z. Wang, N. Liang, Y. Pei, A. Xuhrat and J. Guo, *Energy Fuels,* 2023, **37**, 12427-12435.
7. J. Zhao, R. Wang, L. Ye, J. Wang, J. Mi, A. Boymirzayev, J. Shodmanov and Y. Feng, *Carbon*, 2026, **247**, 120915
8. T. G. Lim, B. H. Seo, S. J. Kim, S. Han, W. Lee and J. W. Suk, *ACS Omega*, 2024, **9**, 8247-8254.
9. Q. Xu, X. Yu, Q. Liang, Y. Bai, Z. H. Huang and F. Kang, *J. Electroanal. Chem.,* 2015, **739**, 84-88.
10. Shilpa and A. Sharma, *RSC Adv.,* 2016, **6**, 78528-78537.
11. X. Li, Y. Zhao, Y. Bai, X. Zhao, R. Wang, Y. Huang, Q. Liang and Z. Huang, *Electrochim. Acta,* 2017, **230**, 445-453.
12. Y. Chen, A. Amiri, J. G. Boyd and M. Naraghi, *Adv. Funct. Mater.,* 2019, **29**, 1901425.
13. M. Zhi, S. Liu, Z. Hong and N. Wu, *RSC Adv.,* 2014, **4**, 43619-43623.
14. J. G. Kim, H. C. Kim, N. D. Kim and M. S. Khil, *Composites, Part B,* 2020, **186**, 107825.
15. Y. Gao, J. Wang, Y. Huang, S. Zhang, S. Zhang and J. Zou, *Appl. Surf. Sci.,* 2023, **638**, 158137.
16. D. Y. Kim, S. Radhakrishnan, S. Yu and B. S. Kim, *Mater. Adv.,* 2023, **4**, 3215-3223.
17. C. Ma, L. Wu, M. Dirican, H. Cheng, J. Li, Y. Song, J. Shi and X. Zhang, *J. Colloid Interface Sci.,* 2021, **586**, 412-422.
18. J. Wei, S. Geng, O. Pitkänen, T. Järvinen, K. Kordas and K. Oksman, *ACS Appl. Energy Mater.,* 2020, **3**, 3530-3540.
19. C. Lai, Z. Zhou, L. Zhang, X. Wang, Q. Zhou, Y. Zhao, Y. Wang, X. F. Wu, Z. Zhu and H. Fong, *Journal of Power Sources,* 2014, **247**, 134-141.
20. M. W. Thielke, S. L. Guzman, J. P. V. Tafoya, E. G. Tamayo, C. I. C. Herazo, O. Hosseinaei and A. J. Sobrido, *Frontiers in Materials,* 2022, **9**, 859872.
21. S. Hérou, M. Crespo and M. Titirici, *CrystEngComm,* 2020, **22**, 1560-1567.
22. S. Hérou, M. Crespo-Ribadeneyra, P. Schlee, H. Luo, L. C. Tanase, C. Roßberg and M. Titirici, *J. Energy Chem.,* 2021, **53**, 36-48.
23. S. Hérou, J. J. Bailey, M. Kok, P. Schlee, R. Jervis, D. J. L. Brett, P. R. Shearing, M. Crespo-Ribadeneyra and M. Titirici, *Adv. Sci.,* 2021, **8**, 2100016.
24. P. Schlee, O. Hosseinaei, D. Baker, A. Landmér, P. Tomani, M. J. Mostazo-López, S. Herou and M. M. Titirici, *Carbon*, 2019, **145**, 470-480.
25. P. Schlee, O. Hosseinaei, C. A. O'Keefe, M. J. Mostazo-López, D. Cazorla-Amorós, S. Herou, P. Tomani, C. P. Grey and M. M. Titirici, *J. Mater. Chem. A,* 2020, **8**, 23543-23554.
26. P. Schlee, S. Herou, R. Jervis, P. R. Shearing, D. J. L. Brett, D. Baker, O. Hosseinaei, P. Tomani, M. M. Murshed, Y. Li, M. J. Mostazo-López, D. Cazorla-Amorós, A. B. J. Sobrido and M. M. Titirici, *Chem. Sci.,* 2019, **10**, 2980-2988.
